# Comparison of sequencing methods and data processing pipelines for whole genome sequencing and minority single nucleotide variant (mSNV) analysis during an influenza A/H5N8 outbreak

**DOI:** 10.1371/journal.pone.0229326

**Published:** 2020-02-20

**Authors:** Marjolein J. Poen, Anne Pohlmann, Clara Amid, Theo M. Bestebroer, Sharon M. Brookes, Ian H. Brown, Helen Everett, Claudia M. E. Schapendonk, Rachel D. Scheuer, Saskia L. Smits, Martin Beer, Ron A. M. Fouchier, Richard J. Ellis

**Affiliations:** 1 Erasmus MC, Department of Viroscience, Rotterdam, the Netherlands; 2 Institute of Diagnostic Virology, Friedrich-Loeffler-Institute, Insel Riems, Germany; 3 European Molecular Biology Laboratory (EMBL), European Bioinformatics Institute (EBI), Wellcome Genome Campus, Hinxton, Cambridge, United Kingdom; 4 Animal and Plant Health Agency (APHA) - Weybridge, Addlestone, Surrey, United Kingdom; University of Georgia, UNITED STATES

## Abstract

As high-throughput sequencing technologies are becoming more widely adopted for analysing pathogens in disease outbreaks there needs to be assurance that the different sequencing technologies and approaches to data analysis will yield reliable and comparable results. Conversely, understanding where agreement cannot be achieved provides insight into the limitations of these approaches and also allows efforts to be focused on areas of the process that need improvement. This manuscript describes the next-generation sequencing of three closely related viruses, each analysed using different sequencing strategies, sequencing instruments and data processing pipelines. In order to determine the comparability of consensus sequences and minority (sub-consensus) single nucleotide variant (mSNV) identification, the biological samples, the sequence data from 3 sequencing platforms and the *.bam quality-trimmed alignment files of raw data of 3 influenza A/H5N8 viruses were shared. This analysis demonstrated that variation in the final result could be attributed to all stages in the process, but the most critical were the well-known homopolymer errors introduced by 454 sequencing, and the alignment processes in the different data processing pipelines which affected the consistency of mSNV detection. However, homopolymer errors aside, there was generally a good agreement between consensus sequences that were obtained for all combinations of sequencing platforms and data processing pipelines. Nevertheless, minority variant analysis will need a different level of careful standardization and awareness about the possible limitations, as shown in this study.

## Introduction

Over the past decade, high-throughput sequencing technologies have evolved, providing faster, cheaper, and less laborious alternatives to obtain (whole genome) DNA and RNA sequences compared to traditional Sanger sequencing [1, 2]. The use of next-generation sequencing (NGS) technologies is continuously expanding and has revolutionized the field of genomics and molecular biology.

In many fields of infectious disease research, nucleotide changes in DNA or RNA sequences are used to monitor genetic adaptions indicative of evolution, the emergence of drug resistance, immune evasion or as a tool in epidemiological tracing [3]. In clinical settings, sequencing information is used to improve diagnostics and prognosis. NGS technologies play an increasingly important role in these processes as clinically or epidemiologically important nucleotide changes can be present in the minority of DNA or RNA sequences only, which might be missed with more traditional (consensus) sequencing methods which determine the most abundant sequence variants in a population. Nucleotide variants that are present in only a minority of the sequenced virus population are referred to as minority Single Nucleotide Variants (mSNVs). These variants, initially occurring due to replication errors, can become fixed in the population when they have some sort of evolutionary advantage, for instance, mutations related to drug resistance. Furthermore, mSNVs can be also used for high-resolution molecular epidemiology, which becomes more and more important for outbreak assessment [4, 5]. Traditional Sanger sequencing for instance has been described to detect minority variants provided they are present in at least 10% of the analysed DNA or RNA strands within a sample [6, 7]. Hence, the use of traditional sequencing methods is usually restricted to obtaining consensus sequences or to determine heterozygosity in diploid organisms. In contrast, NGS technologies are able to detect low frequency mSNVs in sequence fragments or even whole genomes. Typically, NGS sensitivity for minority sequence variant identification is restricted to a level of variation of 0.1–1%, mainly due to sequencing related background errors [8–10], but sensitivity can be increased using sophisticated approaches like circle sequencing [11] or improved bioinformatic analysis workflows [10]. The reliability of mSNV analysis using NGS methods is influenced by many factors, like the quantity and quality of the input sample, the laboratory procedures, the type of sequencing platform and the software and settings used to analyse the raw sequence data.

Due to the technical improvements, NGS technologies have become more important as diagnostic tools to characterize pathogens in outbreak situations. However, the increasing use of these technologies to address new and important (outbreak related) research and surveillance questions emphasizes the need to determine the reproducibility of, and the important technical considerations affecting, outcomes obtained by different laboratories following different protocols. Given this, comparative studies focusing on different platforms and data analysis methods are essential to cross-validate different methodologies and determine the reliability of newly obtained data. In addition, there is a growing need (as exemplified by the recent Ebola and Zika virus outbreaks) to share also comprehensive sequencing data as quickly as possible to help with source attribution and developing control strategies. However, the underlying technologies and methods used for NGS are still diverse and there is a strong demand for harmonization of laboratory procedures and approaches for a reliable and optimized analysis of the data.

This study is part of the European Union’s HORIZON 2020 project “COMPARE” (http://www.compare-europe.eu/), aiming to improve the analytical tools for emerging zoonotic pathogens and its underpinning research. Here, the comparability of NGS output data obtained from different sequence approaches were evaluated and demonstrated suitable sharing strategies for comprehensive NGS data sets. In November 2014, a newly emerging strain of highly pathogenic avian influenza (HPAI) virus was detected in several European countries [12, 13]. In the United Kingdom [14], Germany [15], and The Netherlands [16–18] this subtype was detected in commercial poultry farms within a few days of one another. In each of those countries, NGS was used to generate whole-genome sequences rapidly after detection, but as the laboratories in each country were working independently, different approaches were used for both sequencing and data analysis, and the data were shared as part of a wider study to determine the likely source of the outbreak [19]. It is important to determine whether the different analytical approaches have any impact on the outcome. Therefore, the aim of this study was to determine how comparable consensus and minority variant results were between laboratories performing their standard analyses, and whether discrepancies could be attributed to the sequence platform (SP), the data processing platform (DPP) or a combination of both. With the lack of a ground truth/gold standard, all datasets obtained were compared amongst each other. The hypothesis we test in this study is that outputs from NGS analysis of viruses will be comparable irrespective of laboratory, sequencing platform and data analysis platform.

Therefore, virus isolates obtained in each of the three countries (United Kingdom, Germany and the Netherlands) were shared between these three partners and subsequently sequenced and analysed in each of the three laboratories according to local procedures. In addition, the use of a specially designed data sharing platform, a COMPARE “Data Hub” at EMBL-EBI, Hinxton UK, was evaluated. This study presents genome coverage data, consensus sequences, the analysis of the comparability of mSNV identifications of the different SPs, and DPPs.

Our hypothesis was confirmed at the consensus sequence level, since consensus sequences could be reproduced independent of the combination of SP and DPP used. However, the identification of minority variants appeared to be poorly reproducible, primarily due to the well-known errors in 454 sequencing, and due to differences induced by the alignment processes in the different DPPs. The interpretation of minority variant analysis thus needs a different level of careful standardization and awareness about the possible limitations as shown in this study.

## Materials and methods

### Experimental design

**Fig 1:**
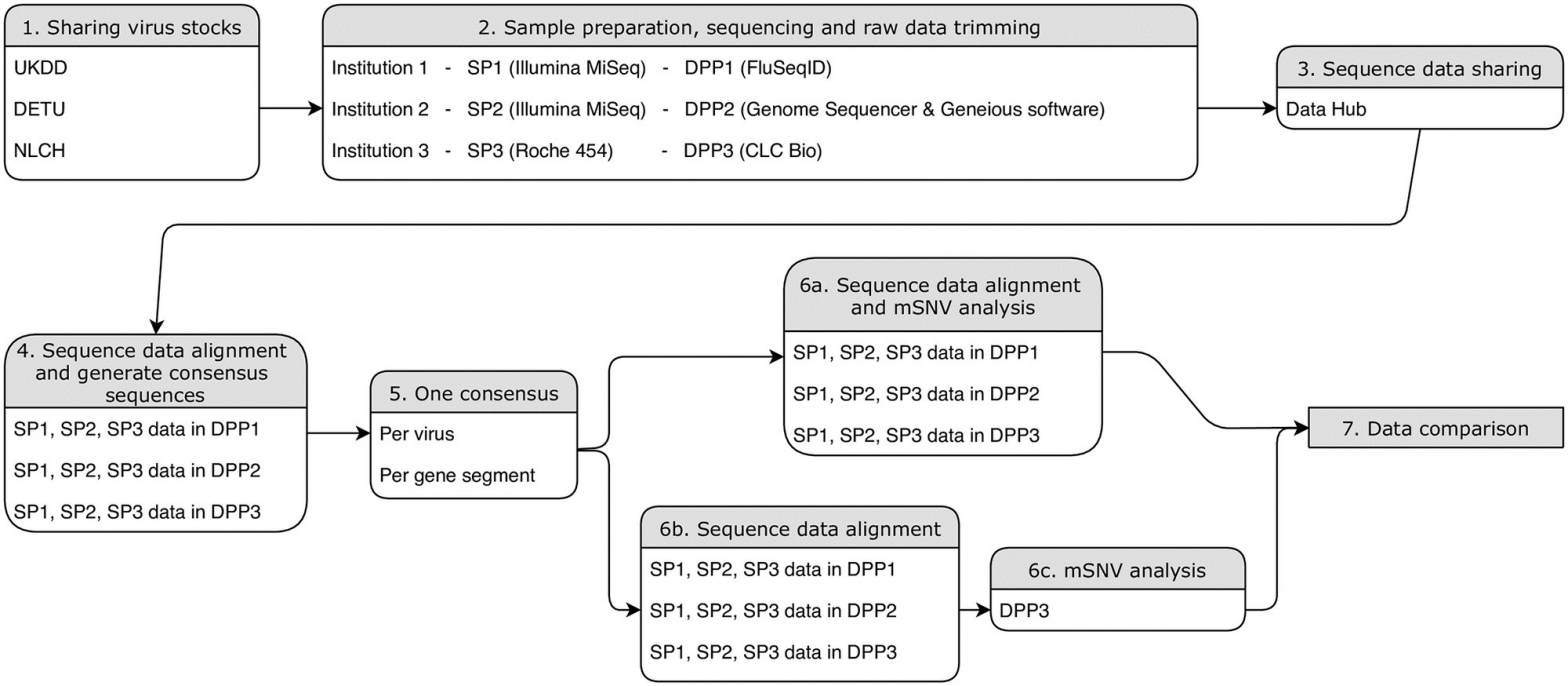
Flowchart of the experimental design. SP: sequence platform; DPP: data processing pipeline.

Three avian influenza A virus isolates that were obtained from three different avian species during the 2014/15 outbreak of HPAI H5N8 virus in Europe were shared among three institutions in the United Kingdom (Animal Plant and Health Agency [APHA]), Germany (Friedrich-Loeffler-Institut [FLI]) and the Netherlands (Erasmus Medical Center [EMC]), later referred to as anonymized institutions I, II and III (Fig 1). All three institutions sequenced all three virus isolates according to their own standard procedures. Adaptors used in the sequencing processes were trimmed off before the raw sequence data files were shared. The sequence data files (*.fastq files), alignment files (*.bam files), sample metadata and experimental metadata were shared between the three laboratories and analysed in their own DPPs yielding sequence datasets for each virus (Table 1). This approach enabled to separate the biological features of the viruses from variation introduced by technical methodology. Data sharing was facilitated via a “Data Hub” provided by the EMBL-EBI’s European Nucleotide Archive (ENA) in the framework of the COMPARE collaborative project; all data were stored and subsequently published in ENA [20] (https://www.ebi.ac.uk/ena, for the accession numbers, see Table 1). ENA is an open repository for sequence and related data and a member of the International Nucleotide Sequence Database Collaboration (INSDC; http://www.insdc.org/) [21]. A full description of the COMPARE Data Hub system is provided in a preprint version of Amid et al. [22]. First, consensus sequences derived from a preliminary analysis were compared and one overarching consensus sequence was determined for each gene segment for each virus. This custom-made consensus was used by all three institutions as the reference genome for undertaking mSNV analysis. The resulting nine mSNV reports (originating from three whole-genome raw data sequences times three DPPs) were combined for all three viruses in one spreadsheet file per virus to check the reproducibility of mSNV identification when using different combinations of SP and DPP. The experimental design is summarized in Fig 1.

**Table 1:** Sample characteristics and accession details.

	UKDD				DETU				NLCH			
**Virus strain**	A/duck/England/36254/2014				A/turkey/Germany/AR2485-L01478/2014				A/chicken/Netherlands/EMC-3/2014			
**Isolation source**	Pooled intestines				Lung tissue				Lung tissue			
**Host Scientific Name**	Anas platyrhynchos				Meleagris gallopavo				Gallus gallus domesticus			
**Host Common Name**	Domestic duck				Turkey				Chicken			
**Collection Date**	14 November 2014				04 November 2014				23 November 2014			
**Collection Country**	United Kingdom				Germany				Netherlands			
**Collection Region**	East Yorkshire				Mecklenburg-Western Pomerania				Ter Aar			
**Influenza Test Method**	MP gene RRT-PCR, H5 RRT-PCR				MP gene RRT-PCR, H5 RRT-PCR				MP gene RRT-PCR, H5 RRT-PCR			
**Culture Status Sample**	Egg passage 1				Egg passage 1				MDCK passage 2			
	**Institution I**	**Institution II**	**Institution III**		**Institution I**	**Institition II**	**Institution III**		**Institution I**	**Institition II**	**Institution III**	
Study Accession*	PRJEB9846	PRJEB12582	PRJEB9687		PRJEB9846	PRJEB12582	PRJEB9687		PRJEB9846	PRJEB12582	PRJEB9687	
Run Accession*	ERR972805	ERR1293054	ERR926712	ERR926713	ERR1354020	ERR1293053	ERR926714	ERR926715	ERR1354021	ERR1293055	ERR926717	ERR926718
**DPP1** *.bam file run accession*	ERR3093746	ERR3093752	ERR9033756		ERR3093744	ERR3093753	ERR3093757		ERR3093745	ERR3093754	ERR3093758	
**DPP2** *.bam file run accession*	ERR2992676	ERR2992677	ERR2992675		ERR2992679	ERR2992680	ERR2992678		ERR2992682	ERR2992683	ERR2992681	
**DPP3** *.bam file run accession*	ERR2985803	ERR2985804	ERR2985802		ERR2985806	ERR2985807	ERR2985805		ERR2985809	ERR2985810	ERR2985808	
Experiment Accession 100k*	ERX315615	ERX2986848	NA	NA	ERX315616	ERX2986847	NA	NA	ERX315617	ERX2986849	NA	NA
**Run Accession 100k** *	ERR3090788	ERR2984276	NA	NA	ERR3090789	ERR2984275	NA	NA	ERR3090790	ERR2984277	NA	NA

* Using the Study Accession numbers in the European Nucleotide Archive all related data files can be accessed, or accessed directly from https://www.ebi.ac.uk/ena/data/view/accession, e.g.: https://www.ebi.ac.uk/ena/data/view/PRJEB9846 (Study Accession Institution I), https://www.ebi.ac.uk/ena/data/view/ERR972805 (Run Accession UKDD Institution I).

### Samples

All samples were obtained from outbreaks in commercial poultry holdings. Isolate A/duck/England/36254/2014 was obtained from pooled intestinal material from index case ducks (*Anas platyrhynchos domesticus*). Tissue homogenate material was inoculated into embryonated chicken eggs and allantoic fluid was harvested at 1 day post-inoculation [14]. The Dutch isolate (A/chicken/Netherlands/EMC-3/2014) was obtained by passaging lung material of a dead commercial layer hen *(Gallus gallus domesticus)* in MDCK cells twice and harvesting the supernatant after approximately 40 hours post-inoculation [23]. The German isolate (A/turkey/Germany/AR2485/2014) originated from lung tissue of a commercially kept turkey (*Meleagris gallopavo)* and was passaged in embryonated chicken eggs [15]. (Table 1).

### Sequencing

#### Institution I: SP1

RNA was extracted using a Qiagen QIAamp viral RNA mini kit (Qiagen, Germany) according to the manufacturers’ instructions except that carrier RNA was omitted from the AVL lysis buffer and the sample was eluted in 50μl RNAse-free water. RNA was then processed to double-stranded cDNA (cDNA Synthesis System, Roche) using random hexamers and purified using magnetic beads (AmpureXP, Beckman Coulter, USA). The double-stranded cDNA was diluted to 0.2 ng/μl and used to produce a sequencing library using the NexteraXT kit (Illumina, USA). Libraries were then sequenced in paired-end mode on an Illumina MiSeq (Illumina, USA), with run lengths varying from 2 x 75 bases (UKDD virus) to 2 x 150 bases (NLCH and DETU viruses) depending on whether time-constraints were implemented to provide a rapid response to an outbreak. Demultiplexing and removal of sequencing adapters was done by the MiSeq RTA software to generate raw fastq files. SP1 process included a limited 12-cycle PCR enrichment of the library. Post-hoc analysis showed that duplication levels were less than 0.02% of the total reads which were considered to have negligible impact on the results.

#### Institution II: SP2

RNA was extracted using a combined approach with TRIzol (Thermo Fisher Scientific, USA) and an RNeasy Kit (Qiagen, Germany). Further concentration and cleaning was done with Agencourt RNAClean XP magnetic beads (Beckman Coulter, USA). RNA was quantified using a Nanodrop UV spectrometer ND-1000 (Peqlab, Germany) and used as template for cDNA synthesis with a cDNA Synthesis System (Roche, Germany) with random hexamers. Fragmentation of the cDNA applying a target size of 300 bp was done with a Covaris M220 ultrasonicator. The sonicated cDNA was used for library preparation using Illumina indices (Illumina, USA) on a SPRI-TE library system (Beckman Coulter, USA) using a SPRIworks Fragment Library Cartridge II (for Roche FLX DNA sequencer; Beckman Coulter, USA) without automatic size selection. Subsequently, upper and lower size exclusion of the library was done with Ampure XP magnetic beads (Beckman Coulter, USA). The libraries were quality checked using High Sensitivity DNA Chips and reagents on a Bioanalyzer 2100 (Agilent Technologies, Germany) and quantified via qPCR with a Kapa Library Quantification Kit (Kapa Biosystems, USA) on a Bio-Rad CFX96 Real-Time System (Bio-Rad Laboratories, USA). SP2 did not amplify sample nor library. Sequencing was done on an Illumina MiSeq using MiSeq reagent kit v3 (Illumina, USA) resulting in paired end sequences with a read length of 300. Demultiplexed and adapter-trimmed reads were used to generate raw fastq files.

#### Institution III: SP3

RNA was extracted using the High Pure RNA isolation kit (Roche Diagnostics, Germany) according to manufacturer’s instructions. RNA was converted to cDNA using the SuperScript III Reverse Transcriptase kit (Invitrogen, Thermo Fisher, USA) as described previously [24], and amplified by PCR using primers covering the full viral genome (S1 Table). All 32 PCR fragments from approximately 400–600 nucleotides in length, were sequenced using the 454/Roche GS-FLX sequencing platform. The PCR fragments were pooled in equimolar ratio and purified using the MinElute PCR Purification kit (Qiagen, Germany) according to the manufacturer’s instructions. Rapid Library preparation, Emulsion PCR and Next Generation 454-sequencing were performed according to instructions of the manufacturer (Roche Diagnostics, Germany). Protocols are described in the following manuals: Rapid Library Preparation Method Manual (Roche; May 2010), emPCR Amplification Method Manual–Lib-L (Roche; May 2010) and Sequencing Method Manual (Roche; May 2010). All three samples were sequenced in one run. Samples were pooled using MID adaptors to determine which sequences came from which sample, each sample was assigned two different MID’s. Demultiplexing and basic trimming was done by CLC-bio software to generate raw fastq files (S1 File).

### Data processing

#### Institution I: DPP1

In the FluSeqID script (https://github.com/ellisrichardj/FluSeqID) the following steps are run automatically: the mapping of raw sequence data to the host genome (BWA v0.7.12-r1039 [25]), extracting reads that do not map to the host (Samtools v1.2 [26]), assembling non-host reads (Velvet v1.2.10 [27]), identification of the closest match for each genome segment (BLAST v2.2.28 [28] using the custom databases generated from the Influenza Research Database as indicated in the GitHub repository), mapping original data to the top reference segments (BWA), calling new consensus sequences (vcf2consensus.pl), performing further iterations of the last two steps to improve new consensus (IterMap), and finally outputting the genome consensus sequence. The data processing pipeline has in-build defaults for k-mer and coverage cut-off for de novo assembly, and the e-value cut-off for BLAST, which can be changed via command line options (see https://github.com/ellisrichardj/FluSeqID). Since the aligner (BWA-MEM) used performs soft-clipping and ignores low quality data, quality trimming is unnecessary. For mSNV analysis, the reads were mapped to the unified consensus using BWA. Samtools was used to generate a pileup file which was then analysed using custom python and R scripts to determine the depth of coverage and basecalls at each position (available at https://github.com/ellisrichardj/MinorVar). The combination of BWA-MEM and samtools was shown to be accurate for SNV identification [29]. In order to be included in the final output the minimum basecall quality was 20 and the minimum mapping quality was 50.

#### Institution II: DPP2

Raw sequence data were analysed and mapped using the Genome Sequencer software suite (v. 3.0; Roche, Mannheim, Germany) and the Geneious software suite (v. 9.0.5; Biomatters, Auckland, New Zealand). Raw reads were trimmed and subsets of each trimmed dataset were assembled *de novo* to generate reference sequences for each data set (Newbler Assembler of Genome Sequencer software suite v. 3.0; Roche, Mannheim, Germany). The trimmed raw influenza virus reads were mapped to the reference sequences (Newbler Mapper of Genome Sequencer software suite v. 3.0; Roche, Mannheim, Germany). The output assemblies were imported into the Geneious software suite (v. 9.0.5; Biomatters, Auckland, New Zealand) for further analysis and processing. Regions of low and high coverage (threshold was 2 x standard deviations from the mean for low and high coverage) and regions of low quality (minimum quality/phred score 20) were evaluated and if necessary, excluded from further analyses. Consensus sequences were generated and annotated using annotated reference sequences. Sequences were compared, and annotations that matched with a similarity (> 90%) were copied. This was done on nucleotide sequences and also for translation in all six reading frames. Annotations were manually inspected and curated. Trimmed raw reads of the datasets or subsets thereof were mapped to the consensus, mapping was fine-tuned and mSNVs were determined using generic SNP finder of the Geneious software suite, applying parameters of maximum p-value of 10^−5^ and filter for strand bias. The threshold for SNP identification was set at 1%, and variants were checked manually for accuracy.

#### Institution III: DPP3

Raw sequence data were analysed and mapped using the CLC Genomics software package, workbench 8 (CLC Bio). Reads obtained by 454 sequencing were sorted by MID adaptor, quality-trimmed, and analysed using the parameters as shown in S1 File. In short, after sorting by MID, the sequence reads were trimmed at 30 nucleotides from the 3′ and 5′ ends to remove all primer sequences. Data from the shared Illumina sequence files had already been trimmed and were imported in CLC Bio without additional processing steps (S1 File). Reads were initially aligned to their own reference sequences that were uploaded during the H5N8 outbreak (Gisaid accession numbers EPI-ISL-169282 (NLCH), EPI-ISL-167904 (UKDD) and EPI-ISL-169273 (DETU)). Consensus sequences were automatically generated by CLC after alignment to the reference, for detailed settings see S1 File. For the mSNV analysis the raw data were mapped to the new custom-made consensus sequences per gene segment per sample. Fastq files of these alignments were shared with the other institutions. The threshold for mSNV identification was set at 1%, and registered minority variants were checked manually for accuracy (minimal quality/phred score 20).

#### Determining the influence of the DPP alignment steps versus DPPs mSNV identification methods

Data processing pipelines process raw data in several steps, roughly divided into trimming, aligning data to a reference sequence, and variant calling (the mSNV identification procedure). In order to determine to what extent the trimming and subsequent alignment processes contributed to the observed differences the nucleotide coverage results obtained by the three DPPs when aligning the same SP raw datasets were compared. To study the influence of the mSNV identification process, quality-trimmed alignment files that had been generated by each DPP and shared as *.bam files were subjected to the mSNV identification process used in DPP3 to determine the differences in mSNV detection output when only the alignment processes differed. DPP3 was randomly picked for this analyses, mSNV detection parameters were set to the institutions default settings for mSNV identification using CLC-bio software and can be seen in the S1 File.

### Data sharing

To test the applicability of real-time sequence data sharing within the COMPARE network, all raw sequence data used in this study were uploaded to and shared via a “Data Hub” in the environment of the European Nucleotide Archive (ENA). Each institution received its own study accession in which all raw sequence data files and metadata files were assigned with individual experimental accession numbers (Table 1). In addition to the sequence data, all trimmed alignment files (*.bam) have been uploaded to the ENA. Using these hubs, sharing between institutions was facilitated and immediate access to the data prior to the public release was possible to enable joint evaluation and comparison. All data files have been made publicly available via the ENA (https://www.ebi.ac.uk/ena).

### Designing the custom-made consensus sequences

Each institution produced a consensus sequence for the 8 influenza gene segments (PB2, PB1, PA, HA, NP, NA, MP, NS) for each of the three viruses. The obtained consensus sequences were aligned using the BioEdit sequence alignment editor (version 7.2.0) [30]. Raw sequence data from each SP were initially aligned to their own reference sequences that were uploaded during the H5N8 outbreak (Gisaid accession numbers EPI-ISL-169282 (NLCH), EPI-ISL-167904 (UKDD) and xxx (DETU)).

### mSNV analysis comparison

For the mSNV analyses the custom-made consensus for each virus isolate was used as a reference for mapping, thereby standardizing positions within the genome to make comparison between institutions easier. To avoid unnecessary increases in analytical time and memory, datasets were down-sampled to 100.000 reads per sample when needed. Each DPP produced a report on the identified mSNVs in a tabulated format. The analysis output files were filtered for mSNVs only, thereby ignoring detected nucleotide insertions and deletions (InDels). There is a current lack of data or evidence-based approaches on how to calculate the required sequence depth (i.e. coverage) for mSNV analyses an evidence-based. In this study, a minimum coverage threshold for the identification of mSNVs was applied. This minimum nucleotide coverage (i.e. number of reads per nucleotide after trimming) was determined using a basic sample size calculation method, n = log β / log *p’* [31]. Here β represents the required power (e.g. for 95% chance of detecting something β = 0.05), and *p’* is 1—the proportion of events that you want to detect. For a 95% certainty of detecting a variant at 1%, a minimum coverage of 298 reads per position is needed. For variants that occur in ≥5% of reads, the number of reads required is >58, and for variants that occur in ≥10% of the reads the minimum coverage is >28. For the mSNV identification literature commonly uses the mSNV cut-off frequencies of ≥10%, ≥5% and ≥1%. However, it needs to be noted that these cut-off values are arbitrary. Therefore, where depth of coverage was sufficient, this study will report mSNV detected with a frequency of ≥1%, but initial comparisons started with positions showing mSNVs with frequencies of ≥10% in at least one of the SP/DPP combinations, followed by those with mSNV of ≥5% -<10%, and lastly those ≥1%-<5%. For all those positions identified, the number of reads and number of variant nucleotides in all other SP/DPP combinations for that position will be noted regardless of frequencies.

## Results

In order to determine the comparability of consensus sequences and mSNV identification the biological samples, the sequence data from 3 SPs and the *.bam quality-trimmed alignment files of raw data of 3 influenza A/H5N8 viruses were shared. All data sets were subsequently analysed in 3 different DPPs. The resulting 9 mSNV reports per virus (3 SP data sets each analysed in 3 DPPs) were evaluated for comparability of mSNV identification using different combinations of SP and DPP.

### Data sharing

Data sharing using the COMPARE “Data Hub” provided by ENA proved to be easy, quick and successful. The “Data Hub” enables the File Transport Protocol (FTP) protected upload and download of large data files and facilitates sharing between collaborators with the possibility to evaluate and compare all data prior to their public release by generating and specifically sharing accession numbers using standard ENA procedures. The Data Hub used an influenza virus sample checklist. In addition, data sets are ultimately made publicly and through the INSDC network globally available and accessible in real-time as required without further upload to a different repository. Full details of the COMPARE Data Hub system are available in a submitted manuscript [22]. In summary, this process was suitable for quick data sharing in an outbreak scenario.

### Designing the custom-made consensus sequences

For each of the 8 gene segments of the 3 viruses separately, 9 initial consensus sequences (3 SPs x 3 DPPs) were generated, resulting in 72 consensus sequences per virus. The custom-made consensus sequence per virus and per gene segment was 1) trimmed to a length represented by all 9 initial consensus sequences and 2) nucleotides had to be identical to at least 6/9 consensus sequences to be included. Although some sequences contained insertions or deletions, those could always be corrected for using the other SP sequences following the criteria mentioned previously. This resulted in a unique custom-made consensus for each gene segment for all three viruses.

### Consensus sequences

When ignoring insertions and deletions in the homopolymer regions of the 454 data for most gene segments the identified consensus sequences were identical regardless of the SP and DPP combinations used with the exemption of the differences mentioned in Table 2. However, the number of insertions and deletions in homopolymer regions of the SP3 sequences were considerable in all 3 viruses. There was no clear difference in the number of insertions and deletions related to homopolymer regions between the different DPPs (20, 17 and 18 for DPP 1, 2 and 3 respectively). Nucleotide differences that were not related to homopolymer regions were only observed for sequences obtained in SP3 and SP2 data when processed in DPP1.

**Table 2:** The differences in consensus sequences obtained from each SP/DPP combination, sorted per virus and per gene segment.

Virus	Segment	Start*	End	Number of InDels at homoplymer regions**	Other nucleotide differences***
**NLCH**	PB2	1	2280	2 (DPP1)	C506A (SP3)
				2 (DPP3)	G2101R (SP3)
	PB1	1	2277	1 (DPP1/DPP2/DPP3)	
				1 (DPP1/DPP2)	A949W (SP3)
				1 (DPP2/DPP3)	2272 ins AAG (SP2)
				1 (DPP3)	
	PA	-6^#^	2190	1 (DPP1/DPP2)	ND
				2 (DPP1)	
	HA	7	1704	1 (DPP2/DPP3)	A427W (SP2)
	NP	1	1497	1 (DPP1)	C420Y (SP3)
	NA	4	1419	ND	ND
	MP	-5^#^	982	ND	ND
	NS	1	838	ND	ND
**DETU**	PB2	1	2287	1 (DPP1/DPP2/DPP3)	2272 Del A (SP3)
				3 (DPP1)	
	PB1	1	2277	1 (DPP1/DPP2/DPP3)	T956C (SP3)
				1 (DPP1)	
				1 (DPP2)	
				1 (DPP3)	
	PA	7	2189	1 (DPP1/DPP2)	ND
	HA	1	1728	1 (DPP2/DPP3)	ND
	NP	1	1497	2 (DPP3)	ND
	NA	1	1413	1 (DPP1)	778 ins CCA (SP3)
	MP	-1^#^	982	1 (DPP2)	ND
	NS	2	838	ND	ND
**UKDD**	PB2	1	2298	1 (DPP1/DPP2/DPP3)	C504T (SP3)
				1 (DPP3)	C506M (SP3)
	PB1	1	2277	1 (DPP1/DPP2/DPP3)	T951W (SP3)
				1 (DPP2/DPP3)	
	PA	1	2151	2 (DPP1)	ND
				1 (DPP2)	
	HA	1	1704	1 (DPP2/DPP3)	ND
	NP	1	1497	1 (DPP3)	T1003Y (SP2)
	NA	4	1420	ND	782 del TA (SP3)
	MP	-5^#^	982	1 (DPP2)	ND
	NS	-5^#^	849	ND	ND

The letter in brackets represents the DPP (column 5) or the SP (column 6) where the insertions/deletions or mutations were detected. InDel: insertions or deletion; SP: Sequence platform; DPP: Data processing pipeline; ND: not detected.

* Start is counted from the ATG start codon;

** Exclusively identified in SP3 sequence data, InDels related to homopolymer regions;

*** Exclusively identified in DPP1;

^#^ '-' indicates the number of nucleotides before the ATG start codon included in the consensus

In summary, the homopolymer errors inherent in the 454 dataset caused problems for all DPPs, as expected. Consensus sequences generated by DPP1 from SP3 (454) data showed some unexpected differences, but it performed well with the SP1 data formats it was designed for and reasonably well with SP2 data. Overall, the consensus sequences can be reproduced by all DPPs using Illumina data but that the analysis of the 454 data from SP3 was more problematic, as it would require editing of the sequences at homopolymer regions. Consensus sequences from this study can be found in the S2 Table.

### The mSNV analysis comparison

#### Nucleotide coverage and the influence of DPP-dependent alignment

**Fig 2:**
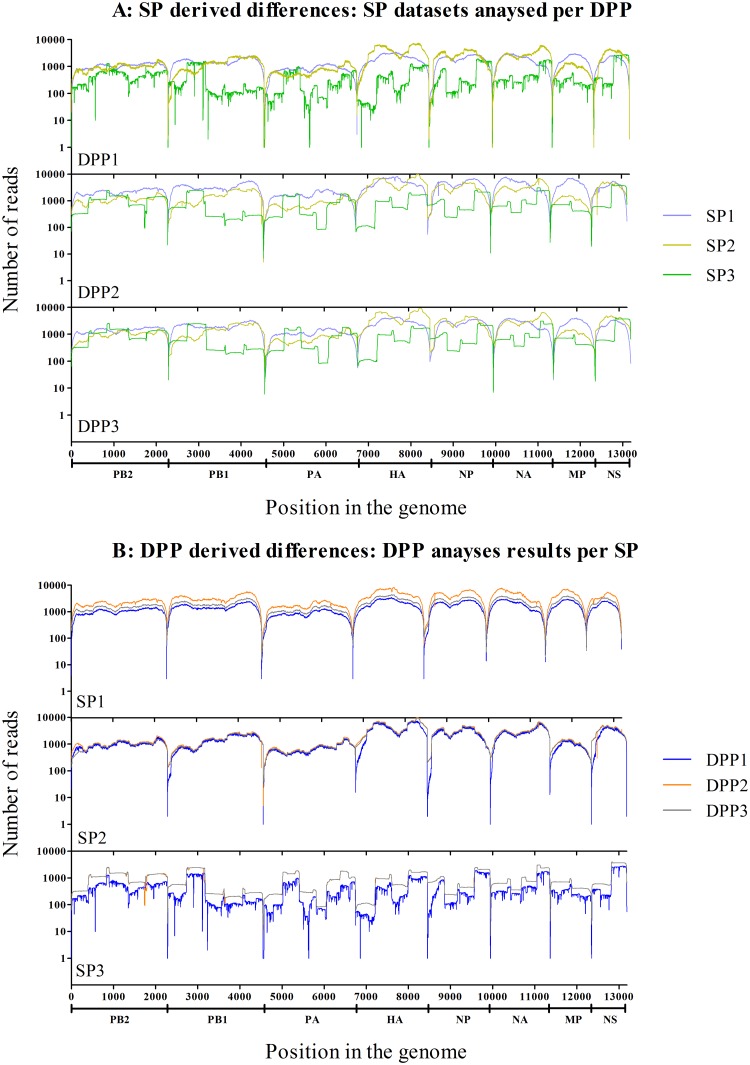
Nucleotide coverage. The non-normalised nucleotide coverage displayed as number of nucleotides per position for full genome sequences of the NLCH virus reads mapped to the NLCH reference sequences. Panel A shows the coverage results for the same SP dataset in the three different DPPs (DPP1: purple; DPP2: orange; DPP3 grey) for each of the SP datasets. Panel B shows the coverage when the same DPP is used to analyse data from the three different SPs (SP1: lilac; SP2: yellow; SP3: green) for each of the DPPs. The X-axis represents the position in the genome, the Y-axis represents the number of sequence reads per position.

The observed number of reads per nucleotide (referred to as nucleotide coverage) differed depending on the SP/DPP combination. All DPPs handled both 454 and Illumina data formats, although some modifications (settings for the bwa mapper to handle single end 454 data) were required for DPP1, which was specifically designed for Illumina paired-end reads. The observed nucleotide coverages showed near to identical profiles for all three viruses. The coverage results obtained from the three different SPs and DPPs for the NLCH virus (Fig 2) and for the other two viruses (S1 Fig) were plotted. In general, lower nucleotide coverage was observed at the termini of each gene segment. The SP3 data showed more variation in nucleotide coverage within gene segments compared to SP1 and SP2 data, due to the sequencing of 32 PCR amplicons. The non-normalised number of raw sequence reads and influenza virus reads per virus per SP can be found in the S3 Table.

The differences in nucleotide coverage were visualized for the three different SP raw datasets analysed with the same DPP (Fig 2A). Overall, SP3 data (green lines) showed a lower coverage compared to SP1 (purple) and SP2 data (yellow). The overall coverage for SP1 and SP2 data was similar with small variations for different viruses and DPPs. The shorter read lengths in SP1 virus data did not appear to have influenced the overall nucleotide coverage substantially.

The differences in nucleotide coverage introduced by different alignment procedures were also assessed, by comparing the coverage results for each SP raw dataset analysed with the three different DPPs (Fig 2B). DPP2 (orange lines) generally retained the highest nucleotide coverage for data from the different SPs. However, DPP3 (grey lines) generally also retained high coverage for SP3 data, for which it was optimized. The nucleotide coverage of SP3 data showed larger variation between the three different DPPs, leading to differences in nucleotide coverage up to 50% depending on the DPP, because DPP1 and DPP2 were not optimized for this SP. Data from SP2 were handled very similar by all three DPPs.

In conclusion, both the SP and the DPP influenced the number of reads per nucleotide position. SP3 showed the lowest output in number of reads compared to SP1 and SP2 Illumina data. The influence of the DPP depended highly on the data input, with best DPP performance for the SP dataset for which it was optimized.

#### The mSNV identification

The mSNV identification thresholds were set to ≥1% in all DPPs. Because of the high number of mSNVs identified, the comparison of these mSNVs started with a manually set arbitrary threshold of ≥10% that was subsequently decreased to ≥5%, and ≥1%. A mSNV position was identified when at least 1 of the SP/DPP combinations showed a variant that exceeded the frequency threshold, and when the coverage at that position exceeded the minimum number of reads needed to detect that variant with a 95% probability, as described previously. The presence of mSNV and coverage for all SP/DPP combinations were compared for each of the positions in which a mSNV had been detected in at least one of the combinations. The coverages indicated for those positions where no mSNVs were detected were derived from the alignment files and were not subjected to possible additional read filtering parameters in the mSNV identification process. The average quality (Q-score/phred score) was set to or exceeding 20.

Ten positions across the three virus genomes were identified with mSNVs occurring in ≥10% of reads. Three of the mSNVs (NLCH:PB2 G1879A, NLCH:PB2 G2101A and DETU:HA T963C) were detected in all SP/DPP combinations but with slightly different relative abundance. The other mSNVs were identified in only one (n = 6) or two (n = 1) of the SP/DPP combinations (Table 3).

**Table 3:** The minority variants occurring in at least one of the sequence platform—Data processing pipelines as a ≥5% variant.

Virus	Position	Sequence platform	Data processing pipeline					
			1		2		3	
			Minor variants	Percentage	Minor variants	Percentage	Minor variants	Percentage
**NLCH**	**PB2.1879 G→A**	1	81/1301	6,2%	246/2716	9,1%	112/1203	9,3%
		2	47/956	4,9%	117/1137	10,3%	114/1064	10,7%
		3	49/530	9,2%	131/1341	9,8%	129/1338	9,6%
	**PB2.2101 G→A**	1	53/1118	4,7%	261/2704	9,7%	110/897	12,3%
		2	21/1578	1,3%	125/1875	6,7%	121/1463	8,3%
		3	13/542	2,4%	199/1433	13,9%	199/1435	13,9%
	**PB2.2277 T→G**	1	ND/479	<1%	86/1008	8,5%	33/190	17,4%
		2	ND/557	<1%	ND/623	<1%	ND/534	<1%
		3	ND/680	<1%	ND/1117	<1%	ND/1024	<1%
	**PB1.87 A→G**	1	ND/818	<1%	ND/1754	<1%	ND/1114	<1%
		2	25/230	10,9%	ND/376	<1%	ND/328	<1%
		3	ND/275	<1%	ND/537	<1%	ND/537	<1%
	**PB1.2240 G→C**	1	ND/664	<1%	54/1341	4,0%	38/418	9,1%
		2	ND/1231	<1%	ND/1271	<1%	ND/1233	<1%
		3	ND/161	<1%	ND/277	<1%	ND/276	<1%
	**PB1.2268 A→G**	1	ND/336	<1%	29/641	4,5%	11/176	6,3%
		2	ND/993	<1%	ND/1026	<1%	ND/1002	<1%
		3	ND/53	<1%	ND/159	<1%	ND/148	<1%
	**HA.104 A→G**	1	ND/733	<1%	ND/1761	<1%	ND/1151	<1%
		2	ND/437	<1%	ND/1370	<1%	ND/1156	<1%
		3	ND/1	<1%	ND/105	<1%	12/105	11,4%
	**HA.1689 T→C**	1	ND/390	<1%	ND/694	<1%	11/217	5,1%
		2	ND/2018	<1%	ND/4083	<1%	ND/3979	<1%
		3	ND/937	<1%	ND/1669	<1%	ND/1680	<1%
	**NP.105 A→G**	1	ND/182	<1%	ND/449	<1%	ND/343	<1%
		2	83/1507	5,5%	ND/1890	<1%	ND/1804	<1%
		3	ND/89	<1%	ND/704	<1%	ND/702	<1%
	**NP.1239 A→T**	1	32/2428	1,3%	279/5410	5,2%	ND/3092	<1%
		2	ND/2345	<1%	ND/2643	<1%	ND/2453	<1%
		3	ND/1711	<1%	ND/2111	<1%	ND/2117	<1%
	**NP.1489 G→A**	1	ND/182	<1%	26/336	7,7%	ND/172	<1%
		2	ND/436	<1%	ND/452	<1%	ND/444	<1%
		3	ND/1320	<1%	ND/1799	<1%	ND/1799	<1%
	**NS.833 A→T**	1	ND/187	<1%	ND/287	<1%	5/88	5,7%
		2	ND/1224	<1%	ND/1327	<1%	ND/1284	<1%
		3	ND/1367	<1%	ND/2430	<1%	ND/2333	<1%
**DETU**	**PB2.1054 T→C**	1	69/1369	5,0%	168/2637	6,4%	97/1304	7,4%
		2	60/1477	4,1%	115/1836	6,3%	99/1605	6,2%
		3	6/392	1,5%	94/2038	4,6%	48/1054	4,6%
	**PB2.2257 A→C**	1	ND/867	<1%	ND/1563	<1%	24/463	5,2%
		2	ND/531	<1%	ND/581	<1%	ND/378	<1%
		3	ND/893	<1%	ND/2286	<1%	ND/1346	<1%
	**PB2.2277 T→G**	1	ND/644	<1%	52/1150	4,5%	27/307	8,8%
		2	ND/418	<1%	ND/472	<1%	ND/284	<1%
		3	ND/1208	<1%	ND/1948	<1%	ND/1209	<1%
	**PB1.14 C→T**	1	ND/144	<1%	48/433	11,1%	ND/239	<1%
		2	ND/90	<1%	ND/355	<1%	ND/304	<1%
		3	ND/562	<1%	ND/792	<1%	ND/496	<1%
	**PB1.23 T→G**	1	ND/207	<1%	30/535	5,6%	ND/315	<1%
		2	ND/103	<1%	ND/365	<1%	ND/319	<1%
		3	ND/699	<1%	ND/950	<1%	ND/609	<1%
	**PB1.87 A→G**	1	ND/744	<1%	ND/1644	<1%	ND/1076	<1%
		2	49/365	13,4%	ND/677	<1%	ND/576	<1%
		3	ND/721	<1%	ND/1156	<1%	ND/793	<1%
	**PB1.2240 G→C**	1	ND/757	<1%	23/1517	1,5%	26/515	5,0%
		2	ND/944	<1%	ND/985	<1%	ND/806	<1%
		3	ND/274	<1%	ND/439	<1%	ND/253	<1%
	**PB1.2268 A→G**	1	5/470	1,1%	33/928	3,6%	22/278	7,9%
		2	ND/798	<1%	ND/829	<1%	ND/671	<1%
		3	ND/109	<1%	ND/259	<1%	ND/123	<1%
	**PB1.2271 A→G**	1	12/446	2,7%	59/901	6,5%	16/263	6,1%
		2	ND/729	<1%	47/810	5,8%	40/649	6,2%
		3	1/32	3,1%	ND/123	<1%	2/83	2,4%
	**HA.867 C→T**	1	59/1533	3,8%	206/3183	6,5%	104/1537	6,8%
		2	59/2031	2,9%	150/2525	5,9%	127/2253	5,6%
		3	11/180	6,1%	48/647	7,4%	28/385	7,3%
	**HA.963 T→C**	1	122/1401	8,7%	446/3071	14,5%	189/1419	13,3%
		2	90/1517	5,9%	318/2189	14,5%	247/1828	13,5%
		3	5/69	7,2%	107/606	17,7%	47/293	16,0%
	**NP.1491 C→A**	1	ND/278	<1%	71/583	12,2%	ND/206	<1%
		2	ND/723	<1%	ND/769	<1%	ND/692	<1%
		3	ND/799	<1%	ND/2031	<1%	ND/1206	<1%
	**NA.65 T→C**	1	19/503	3,8%	52/1229	4,2%	16/467	3,4%
		2	20/662	3,0%	50/1104	4,5%	45/992	4,5%
		3	24/557	4,3%	53/1099	4,8%	37/727	5,1%
	**NA.78 T→C**	1	23/599	3,8%	57/1403	4,1%	20/557	3,6%
		2	21/692	3,0%	55/1147	4,8%	50/1033	4,8%
		3	23/580	4,0%	51/1124	4,5%	37/735	5,0%
	**NA.89 T→C**	1	23/713	3,2%	55/1670	3,3%	22/651	3,4%
		2	23/798	2,9%	56/1261	4,4%	50/1134	4,4%
		3	24/580	4,1%	55/1196	4,6%	40/775	5,2%
	**NA.117 T→C**	1	37/908	4,1%	87/2140	4,1%	36/818	4,4%
		2	28/1102	2,5%	67/1631	4,1%	ND/1459	<1%
		3	22/531	4,1%	57/1276	4,5%	42/812	5,2%
	**NA.126 T→C**	1	37/983	3,8%	83/2294	3,6%	36/876	4,1%
		2	31/1126	2,8%	72/1676	4,3%	65/1502	4,3%
		3	26/519	5,0%	62/1395	4,4%	43/812	5,3%
**UKDD**	**PB2.2277 T→G**	1	ND/415	<1%	28/507	5,5%	ND/475	<1%
		2	ND/589	<1%	ND/620	<1%	ND/601	<1%
		3	ND/1140	<1%	ND/1996	<1%	ND/2065	<1%
	**PB1.87 A→G**	1	ND/387	<1%	ND/440	<1%	ND/439	<1%
		2	26/327	8,0%	32/395	8,1%	ND/351	<1%
		3	ND/617	<1%	ND/1133	<1%	ND/1136	<1%
	**PB1.728 C→A**	1	ND/750	<1%	ND/832	<1%	ND/836	<1%
		2	ND/776	<1%	52/928	5,6%	ND/829	<1%
		3	ND/2459	<1%	ND/4290	<1%	ND/4293	<1%
	**PB1.730 C→T**	1	ND/742	<1%	ND/824	<1%	ND/826	<1%
		2	ND/767	<1%	57/1008	5,7%	ND/832	<1%
		3	ND/2339	<1%	ND//4286	<1%	ND/4289	<1%
	**PB1.883 G→C**	1	ND/942	<1%	ND/997	<1%	ND/997	<1%
		2	ND/1689	<1%	ND/1865	<1%	ND/1760	<1%
		3	ND/2479	<1%	47/690	6,8%	ND/3681	<1%
	**PA.49 G→C**	1	ND/103	<1%	6/117	5,1%	ND/115	<1%
		2	ND/337	<1%	ND/435	<1%	ND/392	<1%
		3	ND/111	<1%	ND/207	<1%	ND/204	<1%
	**PA.82 C→T**	1	ND/155	<1%	ND/180	<1%	ND/177	<1%
		2	ND/695	<1%	ND/809	<1%	ND/745	<1%
		3	ND/64	<1%	ND/247	<1%	30/248	12,1%
	**NS.811 G→T**	1	ND/221	<1%	17/270	6,3%	ND/249	<1%
		2	ND/2452	<1%	ND/2725	<1%	ND/2557	<1%
		3	ND/3117	<1%	ND/4125	<1%	ND/4139	<1%

Colours display the variant frequency with ≥10% (green), 5–10% (purple) and <5% (pink). ND: not detected.

Thirty-seven positions were identified with mSNVs occurring in ≥5% of reads. Of those, the same mSNV was identified in all SP/DPP combinations for 9 positions (24,3%), in seven or eight of the SP/DPP combinations for 2 positions (5,4%) and in at least two SP/DPP combinations for 19 positions (51.4%), although not always in a frequency of ≥5%. However, for 18 positions (48.6%) the mSNV was not reproduced at a ≥1% frequency in any of the other SP/DPP combinations (Table 3). Focussing on the separate SP data analysed in the 3 DPPs, most of the identified positions with ≥5% mSNVs in at least 1 SP/DPP combination were identified in SP1 data (47%) followed by SP2 (29%) and SP3 (24%) data.

Looking at the ≥5% mSNV reproducibility per SP dataset in all three DPPs within these thirty-seven positions, forty-eight SP datasets showed a ≥5% mSNV in at least one of the DPP outputs. Additionally, for eleven positions, all in the DETU virus, the variant was reproduced by all DPPs, however at a <5% frequency (for instance SP3 data at PB2.1054, and SP1 and SP2 data at NA.65) In 53% (31/59) of cases the same mSNVs from 1 SP dataset was reproduced in all three DPP’s in at least a ≥1% frequency, in 31% (18/59) of cases the variant was only detected in 1 DPP even though coverage in the other DPPs was theoretically high enough to detect variants at a 1% level.

**Fig 3:**
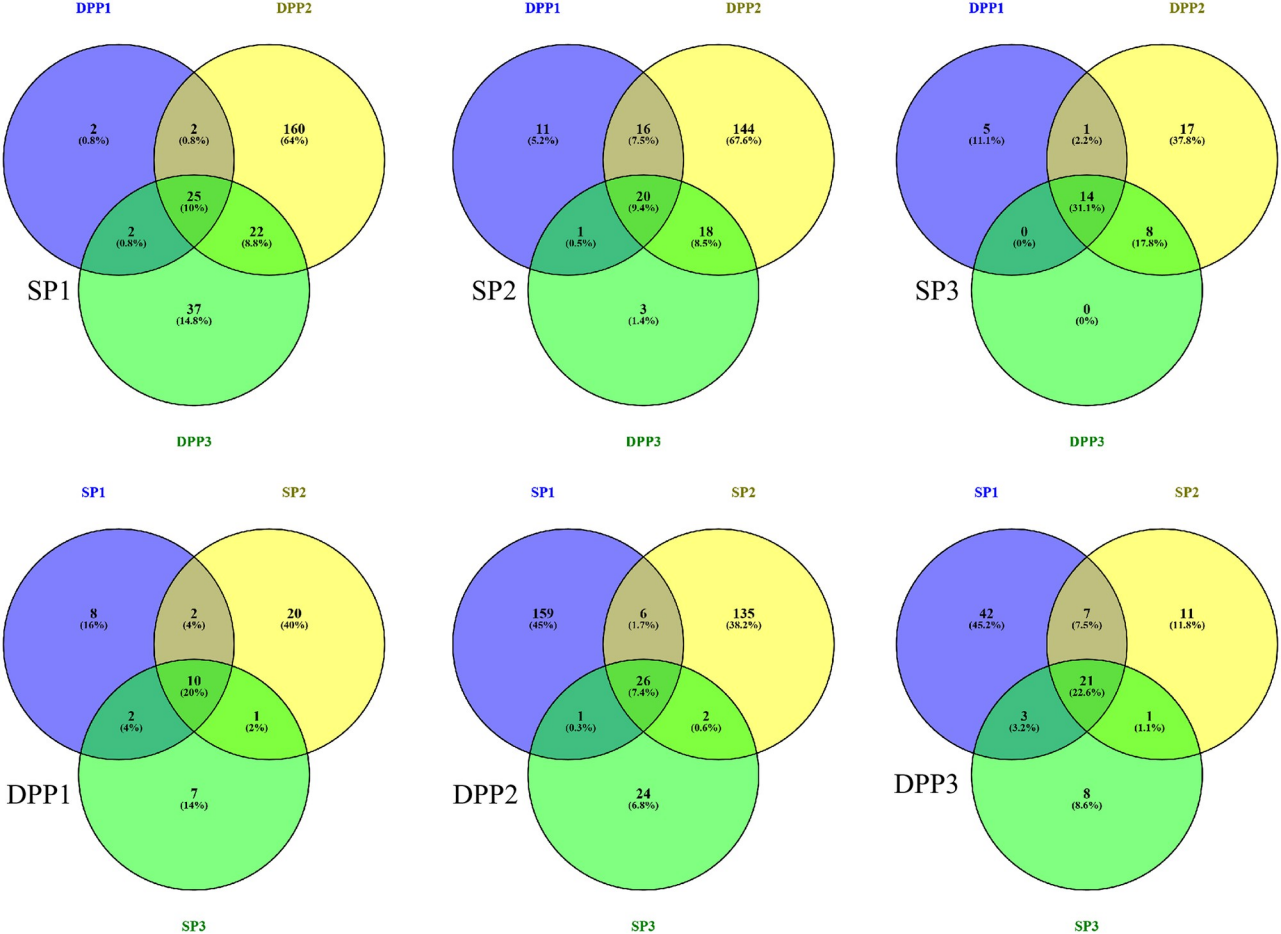
The reproducibility of ≥1% variants with sufficient coverage (>298) for all sequence data combined. Each figure shows the number of ≥1% variants detected per sequence platform (SP, top row) and data processing pipeline (DPP, bottom row) for SP1/DPP1 (left column), SP2/DPP2 (middle column), and SP3/DPP3 (right column). The colours represent the different DPPs and SPs respectively, in which the >1% variants were detected: SP1/DPP1 (purple), SP2/DPP2 (yellow) and SP3/DPP3 (green). Positions with ≥1% variants that were identified in more than one of the SPs or DPPs respectively are displayed in the overlapping coloured areas, the centre part representing the number of ≥1% variants that were detected with all three DPPs (top row) or SPs (bottom row). The total number of positions with ≥1% variants detected was 250in SP1, 213 in SP2, 45 in SP3, and 50 in DPP1, 353 in SP2, and 93 in SP3. This figure was produced using Venny 2.1.

Lowering the threshold value to a mSNV frequency of ≥1% resulted in a large increase in the number of positions identified with mSNVs. To investigate the reproducibility of these mSNVs, the data for all 3 viruses was combined per SP in the three DPPs (influence of DPP), and per DPP analysing data from the three SPs (influence of SP). The genome positions with ≥1% variants were listed per SP/DPP combination and entered in the program Venny 2.1 that calculated the overlapping positions as a fraction of the total number of positions between the SP/DPP combinations as compared to the total number of positions, resulting in Fig 3. It needs to be noted that especially SP3 did not always reach the minimum coverage requirements and may therefore not be suitable to detect low-frequency variants with (see also Table 4). Positions where the coverage in one or more of the nine SP/DPP combinations didn’t meet the minimum required coverage of 298 were not included in the comparison in Fig 3. The reproducibility of ≥1% variants using one SP dataset in all three DPPs was 10%, 9.4% and 31.1% for SP1, SP2 and SP3 sequences, respectively. The reproducibility of ≥1% variants using raw data of a virus sequenced in three different SPs was 20%, 7.4% and 22.6% for DPP1, DPP2 and DPP3 respectively (Fig 3). Most ≥1% variants were not reproduced by any of the other DPPs processing the same SP data (~75%) for SP1 and SP2 data. This was less for SP3 data but this might be due to the fact that many positions identified in SP3 data did not meet the minimum coverage criteria and were therefore discarded.

**Table 4:** The minority variants occurring in at least one of the sequence platform—Data processing pipelines as a ≥1% variant in the HA segment of the DETU sample with a minimum coverage of 298 reads at that position.

Position	Sequence platform	Data processing pipeline					
		1		2		3	
		Minor variants	Percentage	Minor variants	Percentage	Minor variants	Percentage
**HA.170 T→A**	1	ND/935	<1%	ND/2191	<1%	ND/1348	<1%
	2	ND/300	<1%	11/693	1,59%	ND/551	<1%
	3	ND/82*	<1%*	ND/245*	<1%*	ND/210*	<1%*
**HA.170 T→C**	1	ND/935	<1%	ND/2191	<1%	ND/1348	<1%
	2	ND/300	<1%	18/693	2,60%	ND/551	<1%
	3	ND/82*	<1%*	ND/245*	<1%*	ND/210*	<1%*
**HA.171 C→A**	1	ND/931	<1%	ND/2184	<1%	ND/1339	<1%
	2	ND/323	<1%	12/698	1,72%	ND/558	<1%
	3	ND/82*	<1%*	ND/245*	<1%*	ND/210*	<1%*
**HA.194 C→A**	1	ND/991	<1%	ND/2397	<1%	ND/1455	<1%
	2	ND/353	<1%	22/701	3,14%	ND/553	<1%
	3	ND/58*	<1%*	ND/250*	<1%*	ND/212*	<1%*
**HA.195 C→A**	1	ND/995	<1%	ND/2390	<1%	ND/1464	<1%
	2	ND/356	<1%	20/701	2,85%	ND/553	<1%
	3	ND/55*	<1%*	ND/250*	<1%*	ND/212*	<1%*
**HA.268 C→T**	1	ND/1140	<1%	ND/2580	<1%	ND/1626	<1%
	2	ND/1293	<1%	25/1563	1,60%	ND/1338	<1%
	3	ND/88*	<1%*	ND/252*	<1%*	ND/212*	<1%*
**HA.272 A→T**	1	ND/1156	<1%	ND/2593	<1%	ND/1639	<1%
	2	17/1424	1,19%	20/1563	1,28%	ND/1404	<1%
	3	ND/81*	<1%*	ND/253*	<1%*	ND/213*	<1%*
**HA.407 G→T**	1	ND/1144	<1%	ND/2364	<1%	ND/1553	<1%
	2	ND/1773	<1%	31/2121	1,46%	ND/1855	<1%
	3	ND/74*	<1%*	ND/237*	<1%*	ND/212*	<1%*
**HA.407 G→A**	1	ND/1144	<1%	27/2364	1,14%	ND/1553	<1%
	2	ND/1773	<1%	ND/2121	<1%	ND/1856	<1%
	3	ND/74*	<1%*	ND/237*	<1%*	ND/212*	<1%*
**HA.418 A→G**	1	ND/1111	<1%	ND/2319	<1%	ND/1492	<1%
	2	29/2195	1,32%	38/2513	1,51%	ND/2197	<1%
	3	ND/69*	<1%*	ND/237*	<1%*	ND/212*	<1%*
**HA.453 T→G**	1	ND/1339	<1%	29/2736	1,06%	ND/1811	<1%
	2	ND/2342	<1%	ND/2695	<1%	ND/2384	<1%
	3	ND/91*	<1%*	ND/193*	<1%*	ND/179*	<1%*
**HA.560 A→G**	1	43/1587	2,71%	113/3385	3,34%	55/1517	3,63%
	2	56/2397	2,34%	145/2912	4,98%	113/2495	4,53%
	3	21/884	2,38%	72/1754	4,10%	43/1245	3,45%
**HA.715 C→T**	1	ND/1663	<1%	62/3832	1,62%	24/1582	1,52%
	2	26/2283	1,14%	55/2722	2,02%	50/2420	2,07%
	3	ND/531	<1%	20/1883	1,06%	15/1245	1,20%
**HA.867 C→T**	1	59/1533	3,85%	206/3183	6,47%	104/1537	6,77%
	2	59/2031	2,90%	150/2525	5,94%	127/2253	5,64%
	3	11/180	6,11%	48/647	7,42%	28/385	7,27%
**HA.963 T→C**	1	122/1401	8,71%	446/3071	14,52%	189/1419	13,32%
	2	90/1517	5,93%	318/2189	14,53%	247/1828	13,51%
	3	5/69	7,25%	107/606	17,66%	47/293	16,04%
**HA.1000 A→C**	1	ND/1409	<1%	48/2962	1,62%	ND/1873	<1%
	2	ND/1629	<1%	ND/1919	<1%	ND/1645	<1%
	3	ND/84*	<1%*	ND/614	<1%	ND/293*	<1%*
**HA.1177 G→A**	1	ND/1222	<1%	ND/2224	<1%	ND/1597	<1%
	2	ND/1652	<1%	34/1901	1,79%	ND/1724	<1%
	3	ND/289*	<1%*	ND/549	<1%	ND/270	<1%
**HA.1183 A→G**	1	ND/1210	<1%	ND/2226	<1%	ND/1589	<1%
	2	ND/1770	<1%	ND/1892	<1%	ND/1723	<1%
	3	ND/280*	<1%*	6/547	1,10%	ND/268*	<1%*
**HA.1199 T→G**	1	ND/1182	<1%	ND/2124	<1%	ND/1518	<1%
	2	ND/1615	<1%	27/1899	1,42%	ND/1732	<1%
	3	ND/296*	<1%*	ND/545		ND/266*	<1%*
**HA.1263 A→G**	1	16/963	1,66%	57/1841	3,10%	26/954	2,73%
	2	26/1924	1,35%	56/2207	2,54%	41/1967	2,08%
	3	ND/1161	<1%	63/2226	2,83%	33/1350	2,44%
**HA.1430 A→G**	1	ND/1311	<1%	ND/2870	<1%	ND/1827	<1%
	2	ND/1498	<1%	36/1924	1,87%	ND/1659	<1%
	3	ND/955	<1%	ND/2391	<1%	ND/1452	<1%
**HA.1455 C→T**	1	ND/1333	<1%	ND/2753	<1%	14/1233	1,14%
	2	ND/1846	<1%	ND/2242	<1%	ND/1895	<1%
	3	ND/1093	<1%	ND/2373	<1%	ND/1449	<1%
**HA.1543 A→G**	1	25/1209	2,07%	94/2757	3,41%	37/1142	3,24%
	2	ND/1660	<1%	56/1857	3,02%	41/1585	2,59%
	3	ND/1182	<1%	ND/3324	<1%	ND/1972	<1%
**HA.1624 C→A**	1	ND/998	<1%	ND/2174	<1%	ND/1478	<1%
	2	ND/1173	<1%	25/1291	1,94%	ND/1120	<1%
	3	ND/2218	<1%	ND/3654	<1%	ND/2244	<1%
**HA.1634 C→A**	1	ND/930	<1%	ND/2032	<1%	ND/1388	<1%
	2	ND/1091	<1%	16/1218	1,31%	ND/1048	<1%
	3	ND/2616	<1%	ND/3704	<1%	ND/2269	<1%
**HA.1638 C→A**	1	ND/932	<1%	ND/1991	<1%	ND/1368	<1%
	2	ND/1083	<1%	15/1180	1,27%	ND/1010	<1%
	3	ND/2600	<1%	ND/3709	<1%	ND/2276	<1%
**HA.1643 T→A**	1	ND/875	<1%	ND/1892	<1%	ND/1291	<1%
	2	ND/1028	<1%	13/1110	1,17%	ND/944	<1%
	3	ND/2612	<1%	ND/3703	<1%	ND/2278	<1%
**HA.1643 T→G**	1	ND/875	<1%	ND/1892	<1%	ND/1291	<1%
	2	ND/1028	<1%	12/1110	1,08%	ND/944	<1%
	3	ND/2612	<1%	ND/3703	<1%	ND/2278	<1%
**HA.1691 G→A**	1	ND/596	<1%	ND/1110	<1%	7/404	1,73%
	2	ND/767	<1%	ND/873	<1%	ND/696	<1%
	3	ND/2499	<1%	ND/3575	<1%	ND/2222	<1%
**HA.1693 A→T**	1	ND/582	<1%	ND/1081	<1%	7/391	1,79%
	2	ND/751	<1%	ND/864	<1%	ND/690	<1%
	3	ND/2310	<1%	ND/3569	<1%	ND/2219	<1%
**HA.1695 T→C**	1	ND/555	<1%	ND/1030	<1%	7/366	1,91%
	2	ND/779	<1%	ND/3557	<1%	ND/688	<1%
	3	ND/1767	<1%	ND/3557	<1%	ND/2220	<1%
**HA.1698 C→T**	1	ND/537	<1%	ND/977	<1%	ND/601	<1%
	2	ND/758	<1%	11/852	1,29%	ND/681	<1%
	3	ND/2260	<1%	ND/3520	<1%	ND/2113	<1%
**HA.1705 A→G**	1	ND/492	<1%	ND/883	<1%	ND/528	<1%
	2	ND/733	<1%	11/832	1,32%	ND/660	<1%
	3	ND/1709	<1%	ND/3300	<1%	ND/2016	<1%

Positions with a too low coverage (<298 reads/position) to detect ≥1% variants are marked with an asterisk (*). Numbers are displayed as [number of variants]/[number of reads on that position]. ND: not detected.

For brevity, the detailed results for the HA gene segment of the DETU virus are shown in Table 4. This virus segment was chosen because it showed the best reproducibility of results for ≥5% minority variants in all SP/DPP combinations. In the DETU HA segment, 33 positions containing a mSNV occurring in ≥1% of reads with sufficient coverage (≥298 reads) were identified. Only 3 of these positions (9%) were identified in all SP/DPP combinations. The majority of the positions (25/33, 76%) were only identified in one of the nine SP/DPP combinations. However, it needs to be noted that the SP3 data coverage was insufficient in all three DPPs to detect ≥1% variants for 11 of those positions (Table 4).

Although a comparison between the frequencies of the detected mSNVs might be appropriate, based on these results where even absence vs. presence of the mSNVs is poorly comparable further in-depth analyses on these frequencies is not performed because of its limited value.

#### Determining the influence of the minor variant detection method

To isolate the effect of just the mSNV identification step in the DPP, independent of the alignment step, quality-trimmed alignment files (*.bam files) of the data (subdivided per virus, per SP and per DPP) were shared and subjected to the same DPP mSNV detection process (in this case DPP3) and compared to the original outcomes from DPP1 and DPP2 (Table 5). In the majority of positions, the different mSNV identification processes did not influence the results, as 84% (119/142) of the mSNVs were identified regardless of the mSNV identification process. Twenty-three mSNVs that were not reproduced by DPP3 mSNV identification analysis, were reproduced when the ‘Direction and position Filters’ in DPP3 were ignored (Table 5, marked with # of ##). These parameters filter out mSNVs when the set criteria for the read direction (variant must occur in both forward and reverse reads), relative read direction (statistical approach of forward/reverse balance) and read position (removal of systemic errors) are not met. However, DPP1 and DPP2 contain similar quality parameters in their mSNV identification process, indicating that different DPPs deal differently with quality parameters, and data could be excluded or included based on the DPP used. In addition, 9 additional mSNVs were identified in the *.bam files compared to the original mSNV outputs. It needs to be noted that the coverage of SP data analysed by DPP1 for positions identified with mSNVs was considerably lower compared to the coverage at that position in the input *.bam files, suggesting additional quality filtering in the mSNV detection step of DPP1. However, the influence on mSNV identification was limited most likely due to the initial high nucleotide coverage.

**Table 5:** The reproducibility of positions with at least one ≥5% variant when alignment files from the respective DPPs are all uploaded into DPP3 for only the mSNV identification process versus when the mSNV identifications are fully performed by the respective DPPs.

Virus	Position	Sequence platform	Data Processing pipeline						Bam file generating processing pipeline					
			1		2		3		1		2		3	
			Minor variants	Percentage	Minor variants	Percentage	Minor variants	Percentage	Minor variants	Percentage	Minor variants	Percentage	Minor variants	Percentage
**NLCH**	**PB2.1879 G→A**	1	81/1301	6,2%	246/2716	9,1%	112/1203	9,3%	132/1375	9,6%	246/2716	9,1%	121/1301	9,3%
		2	47/956	4,9%	117/1137	10,3%	114/1064	10,7%	119/1122	10,6%	117/1137	10,3%	114/1064	10,7%
		3	49/530	9,2%	131/1341	9,8%	129/1338	9,6%	54/542	10,0%	131//1341	9,8%	129/1338	9,6%
	**PB2.2101 G→A**	1	53/1118	4,7%	261/2704	9,7%	110/897	12,3%	138/1180	11,7%	261/2704	9,7%	121/1086	11,1%
		2	21/1578	1,3%	125/1875	6,7%	121/1463	8,3%	ND/1856##	<1%	ND/1850#	<1%	121/1463	8,3%
		3	13/542	2,4%	199/1433	13,9%	199/1435	13,9%	87/625	13,9%	199/1433	13,9%	199/1435	13,9%
	**PB2.2277 T→G**	1	ND/479	<1%	86/1008	8,5%	33/190	17,4%	ND/849	<1%	ND/1008##	<1%	37/281	13,2%
		2	ND/557	<1%	ND/623	<1%	ND/534	<1%	ND/619	<1%	ND/623	<1%	ND/534	<1%
		3	ND/680	<1%	ND/1117	<1%	ND/1024	<1%	ND/708	<1%	ND/1117	<1%	ND/1027	<1%
	**PB1.87 A→G**	1	ND/818	<1%	ND/1754	<1%	ND/1114	<1%	ND/1264	<1%	ND/1753	<1%	ND/1114	<1%
		2	25/230	10,9%	ND/376	<1%	ND/328	<1%	ND/368##	<1%	ND/376	<1%	ND/328	<1%
		3	ND/275	<1%	ND/537	<1%	ND/537	<1%	ND/278	<1%	ND/537	<1%	ND/537	<1%
	**PB1.2240 G→C**	1	ND/664	<1%	54/1341	4,0%	38/418	9,1%	ND/1004	<1%	ND/1341#	<1%	46/486	9,5%
		2	ND/1231	<1%	ND/1271	<1%	ND/1233	<1%	ND/1277	<1%	ND/1271	<1%	ND/1235	<1%
		3	ND/161	<1%	ND/277	<1%	ND/276	<1%	ND/163	<1%	ND/277	<1%	ND/276	<1%
	**PB1.2268 A→G**	1	ND/336	<1%	29/641	4,5%	11/176	6,3%	15/322*	4,66%*	37/641	5,8%	13/213	6,1%
		2	ND/993	<1%	ND/1026	<1%	ND/1002	<1%	ND/1025	<1%	ND/1026	<1%	ND/1002	<1%
		3	ND/53	<1%	ND/159	<1%	ND/148	<1%	ND/90	<1%	ND/159	<1%	ND/151	<1%
	**PA.2167 T→G**	1	ND/141	<1%	ND/288	<1%	ND/154	<1%	ND/235	<1%	21/288*	7,29%*	ND/154	<1%
		2	ND/757	<1%	ND/807	<1%	ND/773	<1%	ND/812	<1%	ND/807	<1%	ND/733	<1%
		3	ND/704	<1%	ND/1070	<1%	ND/1077	<1%	ND/714	<1%	ND/1070	<1%	ND/1078	<1%
	**HA.104 A→G**	1	ND/733	<1%	ND/1761	<1%	ND/1151	<1%	ND/1175	<1%	ND/1761	<1%	ND/1135	<1%
		2	ND/437	<1%	ND/1370	<1%	ND/1156	<1%	ND/1326	<1%	ND/1369	<1%	ND/1142	<1%
		3	ND/1	<1%	ND/105	<1%	12/105	11,4%	ND/6	<1%	ND/105	<1%	12/105	11,4%
	**HA.1689 T→C**	1	ND/390	<1%	ND/694	<1%	11/217	5,1%	ND/610	<1%	ND/694	<1%	13/260	5,0%
		2	ND/2018	<1%	ND/4083	<1%	ND/3979	<1%	ND/4045	<1%	ND/4081	<1%	ND/3979	<1%
		3	ND/937	<1%	ND/1669	<1%	ND/1680	<1%	ND/1106	<1%	ND/1669	<1%	ND/1680	<1%
	**NA.3 T→C**	1	ND/32	<1%	ND/105	<1%	ND/49	<1%	ND/92	<1%	7/105*	6,67%*	ND/49	<1%
		2	ND/6	<1%	ND/313	<1%	ND/297	<1%	ND/305	<1%	ND/313	<1%	ND/297	<1%
		3	ND/2	<1%	ND/25	<1%	ND/25	<1%	ND/6	<1%	ND/25	<1%	ND/25	<1%
	**NP.105 A→G**	1	ND/182	<1%	ND/449	<1%	ND/343	<1%	ND/374	<1%	6/449*	1,34%*	ND/343	<1%
		2	83/1507	5,5%	ND/1890	<1%	ND/1804	<1%	ND/1866##	<1%	ND/1890	<1%	ND/1805	<1%
		3	ND/89	<1%	ND/704	<1%	ND/702	<1%	ND/246	<1%	ND/704	<1%	ND/703	<1%
	**NP.1239 A→T**	1	32/2428	1,3%	279/5410	5,2%	ND/3092	<1%	ND/3372##	<1%	ND/5410#	<1%	ND/3092	<1%
		2	ND/2345	<1%	ND/2643	<1%	ND/2453	<1%	ND/2626	<1%	ND/2643	<1%	ND/2453	<1%
		3	ND/1711	<1%	ND/2111	<1%	ND/2117	<1%	ND/1712	<1%	ND/2111	<1%	ND/2117	<1%
	**NP.1489 G→A**	1	ND/182	<1%	26/336	7,7%	ND/172	<1%	ND/242	<1%	26/376*	6,9%	ND/172	<1%
		2	ND/436	<1%	ND/452	<1%	ND/444	<1%	ND/451	<1%	ND/451	<1%	ND/444	<1%
		3	ND/1320	<1%	ND/1799	<1%	ND/1799	<1%	ND/1325	<1%	ND/1799	<1%	ND/1799	<1%
	**NS.827 C→T**	1	ND/249	<1%	19/419	4,5%	ND/205	<1%	ND/365	<1%	21/412	5,3%	ND/205	<1%
		2	ND/1316	<1%	ND/1423	<1%	ND/1375	<1%	ND/1427	<1%	ND/1422	<1%	ND/1375	<1%
		3	ND/2091	<1%	ND/2901	<1%	ND/2757	<1%	ND/2293	<1%	ND/2898	<1%	ND/2929	<1%
	**NS829 G→T**	1	ND/221	<1%	19/380	5,0%	ND/179	<1%	ND/328	<1%	19/376	5,4%	ND/179	<1%
		2	ND/1302	<1%	ND/1391	<1%	ND/1341	<1%	ND/1388	<1%	ND/1389	<1%	ND/1341	<1%
		3	ND/2117	<1%	ND/2852	<1%	ND/2727	<1%	ND/2279	<1%	ND/2852	<1%	ND/2880	<1%
	**NS.833 A→T**	1	ND/187	<1%	ND/287	<1%	5/88	5,7%	ND/259	<1%	11/257*	4,28%*	5/96	5,2%
		2	ND/1224	<1%	ND/1327	<1%	ND/1284	<1%	ND/1314	<1%	ND/1322	<1%	ND/1284	<1%
		3	ND/1367	<1%	ND/2430	<1%	ND/2333	<1%	ND/1779	<1%	ND/2430	<1%	ND/2360	<1%
**DETU**	**PB2.900 A→G**	1	38/1335	2,9%	136/2740	5,0%	61/1231	5,0%	68/1328	5,12	136/2740	4,96	65/1322	4,92
		2	35/1645	2,1%	77/1800	4,3%	66/1629	4,1%	70/1775	4,0%	77/1800	4,3%	66/1629	4,1%
		3	30/861	3,5%	86/2308	3,7%	47/1245	3,8%	ND/1001##	<1%	ND/2308#	<1%	47/1245	3,8%
	**PB2.1054 T→C**	1	69/1369	5,0%	168/2637	6,4%	97/1304	7,4%	105/1393	7,5%	168/2637	6,4%	100/1376	7,3%
		2	60/1477	4,1%	115/1836	6,3%	99/1605	6,2%	113/1810	6,2%	115/1836	6,3%	99/1605	6,2%
		3	6/392	1,5%	94/2038	4,6%	48/1054	4,6%	32/524	6,1%	94/2038	4,6%	48/1054	4,6%
	**PB2.2257 A→C**	1	ND/867	<1%	ND/1563	<1%	24/463	5,2%	ND/1447	<1%	ND/1562	<1%	26/472	5,5%
		2	ND/531	<1%	ND/581	<1%	ND/378	<1%	ND/588	<1%	ND/580	<1%	ND/378	<1%
		3	ND/893	<1%	ND/2286	<1%	ND/1346	<1%	ND/1341	<1%	ND/2185	<1%	ND/1347	<1%
	**PB2.2277 T→G**	1	ND/644	<1%	52/1150	4,5%	27/307	8,8%	ND/1062	<1%	ND/1150#	<1%	28/381	7,4%
		2	ND/418	<1%	ND/472	<1%	ND/284	<1%	ND/474	<1%	ND/472	<1%	ND/284	<1%
		3	ND/1208	<1%	ND/1948	<1%	ND/1209	<1%	ND/1251	<1%	ND/1948	<1%	ND/1214	<1%
	**PB1.14 C→T**	1	ND/144	<1%	48/433	11,1%	ND/239	<1%	ND/362	<1%	48/433	11,1%	ND/239	<1%
		2	ND/90	<1%	ND/355	<1%	ND/304	<1%	ND/345	<1%	ND/351	<1%	ND/304	<1%
		3	ND/562	<1%	ND/792	<1%	ND/496	<1%	ND/633	<1%	ND/655	<1%	ND/504	<1%
	**PB1.23 T→G**	1	ND/207	<1%	30/535	5,6%	ND/315	<1%	ND/470	<1%	30/535	5,6%	ND/315	<1%
		2	ND/103	<1%	ND/365	<1%	ND/319	<1%	ND/365	<1%	4/365*	1,96%*	ND/319	<1%
		3	ND/699	<1%	ND/950	<1%	ND/609	<1%	ND/702	<1%	ND/950	<1%	ND/609	<1%
	**PB1.87 A→G**	1	ND/744	<1%	ND/1644	<1%	ND/1076	<1%	ND/1218	<1%	ND/1644	<1%	ND/1076	<1%
		2	49/365	13,4%	ND/677	<1%	ND/576	<1%	13/638	2,0%	ND/674	<1%	ND/576	<1%
		3	ND/721	<1%	ND/1156	<1%	ND/793	<1%	ND/731	<1%	ND/1156	<1%	ND/793	<1%
	**PB1.2240 G→C**	1	ND/757	<1%	23/1517	1,5%	26/515	5,0%	ND/1266	<1%	ND/1515#	<1%	28/631	4,4%
		2	ND/944	<1%	ND/985	<1%	ND/806	<1%	ND/994	<1%	ND/984	<1%	ND/806	<1%
		3	ND/274	<1%	ND/439	<1%	ND/253	<1%	ND/301	<1%	ND/439	<1%	ND/253	<1%
	**PB1.2268 A→G**	1	5/470	1,1%	33/928	3,6%	22/278	7,9%	28/420	6,7%	ND/928##	<1%	23/354	6,5%
		2	ND/798	<1%	ND/829	<1%	ND/671	<1%	ND/839	<1%	ND/829	<1%	ND/671	<1%
		3	ND/109	<1%	ND/259	<1%	ND/123	<1%	ND/193	<1%	ND/259	<1%	ND/126	<1%
	**PB1.2271 A→G**	1	12/446	2,7%	59/901	6,5%	16/263	6,1%	29/413	7,0%	59/901	6,6%	21/336	6,3%
		2	ND/729	<1%	47/810	5,8%	40/649	6,2%	43/750*	5,73%*	47/810	5,8%	40/649	6,2%
		3	1/32	3,1%	ND/123	<1%	2/83	2,4%	5/75	6,7%	5/124*	4,03%*	2/83	2,4%
	**HA.867 C→T**	1	59/1533	3,8%	206/3183	6,5%	104/1537	6,8%	112/1584	7,1%	206/3183	6,5%	109/1573	6,9%
		2	59/2031	2,9%	150/2525	5,9%	127/2253	5,6%	144/2502	5,8%	150/2525	5,9%	127/2253	5,6%
		3	11/180	6,1%	48/647	7,4%	28/385	7,3%	13/182	7,1%	48/647	7,4%	28/385	7,3%
	**HA.963 T→C**	1	122/1401	8,7%	446/3071	14,5%	189/1419	13,3%	200/1468	13,6%	446/3071	14,5%	193/1455	13,3%
		2	90/1517	5,9%	318/2189	14,5%	247/1828	13,5%	308/2165	14,2%	318/2189	14,5%	247/1828	13,5%
		3	5/69	7,2%	107/606	17,7%	47/293	16,0%	12/81	14,8%	107/606	17,7%	47/293	16,0%
	**NP.1491 C→A**	1	ND/278	<1%	71/583	12,2%	ND/206	<1%	ND/390	<1%	ND/579#	<1%	ND/206	<1%
		2	ND/723	<1%	ND/769	<1%	ND/692	<1%	ND/766	<1%	ND/769	<1%	ND/692	<1%
		3	ND/799	<1%	ND/2031	<1%	ND/1206	<1%	ND/858	<1%	ND/2031	<1%	ND/1206	<1%
	**NA.65 T→C**	1	19/503	3,8%	52/1229	4,2%	16/467	3,4%	22/535	4,1%	52/1229	4,2%	20/540	3,7%
		2	20/662	3,0%	50/1104	4,5%	45/992	4,5%	52/1063	4,9%	50/1104	4,5%	45/992	4,5%
		3	24/557	4,3%	53/1099	4,8%	37/727	5,1%	28/584	4,8%	53/1099	4,8%	37/727	5,1%
	**NA.78 T→C**	1	23/599	3,8%	57/1403	4,1%	20/557	3,6%	23/622	3,7%	57/1403	4,1%	24/638	3,8%
		2	21/692	3,0%	55/1147	4,8%	50/1033	4,8%	54/1109	4,9%	55/1147	4,8%	50/1033	4,8%
		3	23/580	4,0%	51/1124	4,5%	37/735	5,0%	27/585	4,6%	ND/1124#	<1%	37/735	5,0%
	**NA.89 T→C**	1	23/713	3,2%	55/1670	3,3%	22/651	3,4%	26/731	3,6%	55/1670	3,3%	26/751	3,5%
		2	23/798	2,9%	56/1261	4,4%	50/1134	4,4%	54/1224	4,4%	56/1261	4,4%	50/1134	4,4%
		3	24/580	4,1%	55/1196	4,6%	40/775	5,2%	28/587	4,8%	55/1196	4,6%	40/775	5,2%
	**NA.117 T→C**	1	37/908	4,1%	87/2140	4,1%	36/818	4,4%	40/914	4,4%	87/2140	4,7%	43/922	4,7%
		2	28/1102	2,5%	67/1631	4,1%	ND/1459	<1%	70/1586	4,4%	67/1631	4,1%	ND/1459	<1%
		3	22/531	4,1%	57/1276	4,5%	42/812	5,2%	28/544	5,2%	ND/1276#	<1%	42/812	5,2%
	**NA.126 T→C**	1	37/983	3,8%	83/2294	3,6%	36/876	4,1%	39/973	4,0%	83/2294	3,6%	43/981	4,4%
		2	31/1126	2,8%	72/1676	4,3%	65/1502	4,3%	75/1616	4,6%	72/1676	4,3%	65/1502	4,3%
		3	26/519	5,0%	62/1395	4,4%	43/812	5,3%	30/537	5,6%	62/1395	4,4%	43/812	5,3%
**UKDD**	**PB2.2277 T→G**	1	ND/415	<1%	28/507	5,5%	ND/475	<1%	ND/503	<1%	ND/507#	<1%	ND/475	<1%
		2	ND/589	<1%	ND/620	<1%	ND/601	<1%	ND/627	<1%	ND/620	<1%	ND/601	<1%
		3	ND/1140	<1%	ND/1996	<1%	ND/2065	<1%	ND/1186	<1%	ND/1996	<1%	ND/2071	<1%
	**PB2.2278 T→G**	1	ND/367	<1%	ND/471	<1%	ND/464##	<1%	ND/465	<1%	ND/471	<1%	17/268	6,3%
		2	ND/581	<1%	ND/613	<1%	ND/581	<1%	ND/621	<1%	ND/588	<1%	ND/581	<1%
		3	ND/1141	<1%	ND/1985	<1%	ND/1993	<1%	ND/1184	<1%	ND/1975	<1%	ND/2004	<1%
	**PB1.87 A→G**	1	ND/387	<1%	ND/440	<1%	ND/439	<1%	ND/451	<1%	ND/417	<1%	ND/439	<1%
		2	26/327	8,0%	32/395	8,1%	ND/351	<1%	33/385	8,6%	ND/395#	<1%	ND/351	<1%
		3	ND/617	<1%	ND/1133	<1%	ND/1136	<1%	ND/622	<1%	ND/1133	<1%	ND/1136	<1%
	**PB1.728 C→A**	1	ND/750	<1%	ND/832	<1%	ND/836	<1%	ND/853	<1%	ND/832	<1%	ND/836	<1%
		2	ND/776	<1%	52/928	5,6%	ND/829	<1%	ND/888	<1%	ND/912##	<1%	ND/829	<1%
		3	ND/2459	<1%	ND/4290	<1%	ND/4293	<1%	ND/2471	<1%	ND/4287	<1%	ND/4292	<1%
	**PB1.730 C→T**	1	ND/742	<1%	ND/824	<1%	ND/826	<1%	ND/844	<1%	ND/824	<1%	ND/826	<1%
		2	ND/767	<1%	57/1008	5,7%	ND/832	<1%	ND/893	<1%	ND/1008#	<1%	ND/832	<1%
		3	ND/2339	<1%	ND//4286	<1%	ND/4289	<1%	ND/2464	<1%	ND/4285	<1%	ND/4284	<1%
	**PB1.883 G→C**	1	ND/942	<1%	ND/997	<1%	ND/997	<1%	ND/1016	<1%	ND/997	<1%	ND/997	<1%
		2	ND/1689	<1%	ND/1856	<1%	ND/1760	<1%	ND/1867	<1%	ND/1856	<1%	ND/1760	<1%
		3	ND/2479	<1%	47/690	6,8%	ND/3681	<1%	ND/2635	<1%	ND/690##	<1%	ND/3697	<1%
	**PA.49 G→C**	1	ND/103	<1%	6/117	5,1%	ND/115	<1%	ND/113	<1%	ND/117#	<1%	ND/115	<1%
		2	ND/337	<1%	ND/435	<1%	ND/392	<1%	ND/441	<1%	ND/434	<1%	ND/392	<1%
		3	ND/111	<1%	ND/207	<1%	ND/204	<1%	ND/113	<1%	ND/206	<1%	ND/206	<1%
	**PA.82 C→T**	1	ND/155	<1%	ND/180	<1%	ND/177	<1%	ND/179	<1%	ND/180	<1%	ND/177	<1%
		2	ND/695	<1%	ND/809	<1%	ND/745	<1%	ND/797	<1%	ND/809	<1%	ND/745	<1%
		3	ND/64	<1%	ND/247	<1%	30/248	12,1%	ND/74	<1%	ND/247	<1%	30/248	12,1%
	**NS.811 G→T**	1	ND/221	<1%	17/270	6,3%	ND/249	<1%	ND/261	<1%	ND/270#	<1%	ND/249	<1%
		2	ND/2452	<1%	ND/2725	<1%	ND/2557	<1%	ND/2742	<1%	ND/2725	<1%	ND/2557	<1%
		3	ND/3117	<1%	ND/4125	<1%	ND/4139	<1%	ND/3188	<1%	ND/4124	<1%	ND/4142	<1%

*Locations containing mSNV detections in the DPP3 mSNV analysis of the bam files but not in the original DPPs; Locations containing ≥1% mSNVs that could be reproduced by deleting DPP3s default ‘Direction and position filters’ with those exactly reproduced (#) and those approximately reproduced but with different coverages and/or variants (##).

To better visualise the differences in coverages and allele counts a graphical display of the data for four positions showing mSNVs in different frequencies for each SP/DPP combination is included in S2 Fig. In general, SNVs were rarely missed due to low coverage, as also high coverage SP/DPP combinations display discrepancies (Tables 3 and 4).

## Discussion

NGS data are used for different applications. Although sequence technologies and the accompanying analysis tools are subjected to rapid development, a lot of follow-up research is based on initial findings. Accuracy and repeatability are key values for proper scientific research but the impact of NGS results also reaches beyond science to clinical settings where important clinical management and treatment decisions are based on such results. In this study the comparability of NGS data analyses were analysed using identical input material per virus but different laboratory workflows from nucleic acid extraction and sequencing to data analysis. In addition, the COMPARE “Data Hub” platform was tested for the purpose of sharing large raw datafiles between institutions in an outbreak situation. Using this platform, raw sequence data files up to the size of 8 Gigabytes, alignment files and metadata files of three influenza A/H5N8 viruses were successfully shared in real-time among 3 institutions to allow independent sequencing and analysis procedures, including mSNV identification, to be performed. The Data Hub is available to all institutions.

The aim of this study was to determine how comparable consensus and minority variant results were between laboratories performing their standard analyses, and whether discrepancies could be attributed to the SP, DPP or a combination of both. With the lack of a ground truth/gold standard, all data obtained were compared amongst each other. Importantly, reliable consensus sequences were generated independently of the SP/DPP combination used, although the well-known artefactual InDels in homopolymer regions in SP3 (Roche 454 genome sequencer) sequence data required manual editing. Such consensus sequences routinely form the basis for a detailed characterization of the influenza strain in an outbreak situation, as they are used for the prediction of pathogenicity and pandemic potential of influenza strains.

In contrast to the reproducible generation of consensus genome sequences, the hypothesis that minority variants could be identified reproducibly has to be rejected. The observed differences were mainly attributed to the alignment processes in the different DPPs. The interpretation of minority variant analysis thus needs a different level of careful standardization and awareness about the possible limitations as shown in this study. Reproducibility of mSNV results appeared to be influenced by both the different SPs (resulting in different sequence depths Fig 2) and DPPs (resulting in differences in alignment and mSNV identification of the same input data, Fig 2 and Table 5). There was limited reproducibility of mSNV identification data, even for relative high frequency mSNVs. As expected, the reproducibility was best (30%) for mSNVs occurring in high frequency (≥10%), and least for the low frequent (≥1%) mSNVs (9.4% to 31.1%). Also, the number of positions with 1–5% mSNVs (with sufficient coverage) was much higher (250 in SP1 data, 213 in SP2 data, and 45 in SP3 data) than the number of positions with >5–10% mSNVs (n = 27) or >10% mSNVs (n = 10).

The set-up of this study allowed many variables to influence the final result. The differences from first laboratory procedures and sample preparations up to the final analysis methods can all have contributed to the observed differences in mSNV identification. At this level, especially with lacking an NGS gold standard, it becomes difficult to determine which identified mSNVs are ‘true variants’ and which could be due to systematic errors introduced by RNA isolation methods, amplification, sequencing or manipulated by data processing pipeline settings. Unsurprisingly, the results of this study imply that the choice of SP influences the final output, but the results from this study also indicate that the DPP, especially the alignment process, influences coverage. The SP and DPP derived differences in coverage are of importance because up to a certain (currently unknown, probably SP/DPP dependent) threshold, a higher coverage will provide a more reliable result about the presence of mSNVs. Although the aim of this study was to explicitly compare the three institutions own standard workflows, some parameters (like the phred score and detection limit) were synchronized between the different DPPs. Moreover, the data from each SP were re-processed in each DPP. However, all DPPs use different underlying algorithms and interpret the set parameters differently which might all contribute to the observed differences. These results are partly in line with previous research that showed the need of NGS result validation and concluded that only those mSNVs with a coverage >100 and a frequency of >40% could be identified by NGS methods without secondary confirmation [32], however, this conclusion was based on using the same sample preparation method within a single laboratory. Another recent study sets the cut-off for intrahost virus diversity at 3% with input of at least 1000 RNA copies and a read depth of at least 400x at each genome position for Illumina sequencing [33].

Although some studies have been published on SP error rates [34–37] and PCR amplification induced variants [38–41], a gold standard system for mSNV analysis is lacking. In addition, the DPPs can alter the data due to elimination or inclusion of certain sequences based on the set quality parameters. Allowing too many low-quality reads or being too stringent on the data will influence the coverage per position and might also influence the accuracy of the mSNV identification rate, especially when the coverage is low [42, 43]. Although a low comparability of mSNVs identified in the different SP and DPP combinations was observed, it can be concluded that 454 (SP3) sequencing has approximately the same accuracy as Illumina (SP1 and 2) sequencing based on the number and percentage of reproducible mSNVs in this dataset when ignoring InDel errors in homopolymer regions. Although, Roche 454 sequencing machines are no longer in production, it added value to include 454 sequencing as an alternative sequence platform with alternative chemistry to Illumina. In addition, because Roche 454 was the first commercially successful next generation sequencing system, it was used in research that served as a fundament for follow-up studies [44]. A comparison of Illumina with newer third or fourth generation sequencing platforms (e.g. Nanopore or Pac Bio) would be interesting in the future. However, the overall error rate remains higher than the shorter read technologies and recent work concludes that these new platforms are currently not suitable for the detection of minor variants [33]. In addition, it would be interesting to compare mSNV results of SPs outputting small sequence reads (like Illumina, 454 and Ion Torrent) to new sequencing techniques that output full-length sequence data (e.g. Nanopore [45]). The latter might be less vulnerable to quality trimming parameters compared to small reads and might provide a more consistent nucleotide coverage over complete gene segment.

For mSNV analyses by different labs, very stringent SP/DPP protocols need to be evaluated, for instance by cross-validating results. To allow a better comparison it would be recommended to create some kind of gold standard by for instance evaluating parameters based on sequencing of technical replicates, and controlled mixes of clones. The mSNV analysis can be valuable for epidemiological tracing, to monitor early evolutionary events, or drug resistance, possibly host adaptation, but this would require reproducibility of study outcomes within and between laboratories. As this is currently not that case, more understanding of biases and errors generated by sample processing (enrichment procedures), sequencing strategy (amplicons, shotgun), sequencing chemistry (each of which have their own internal error rates) and the approach to data processing and analysis is needed. Understanding the parameters and thresholds in the software can be difficult and a systematic study using a pipeline where the effect of changing each of these parameters both individually and in combination is required to determine the optimal settings for minor variant analysis.

As alternate high-throughput sequencing technologies arise there will be a need to understand inherent error profiles and how those are handled in data processing approaches. Cross-validation should be supported by international proficiency tests on NGS techniques including mSNV analyses that would be instrumental in validation of results and may foster the trust in NGS-based diagnostics.

## Supporting information

S1 Table PCR primers used in SP3 to cover the influenza A H5N8 gene segments. (PDF)

S2 Table SP/DPP overarching consensus sequences. (PDF)

S3 Table Number of raw sequences and influenza virus reads per SP per virus. (PDF)

S1 File DPP3 Sequence analysis protocol. (PDF)

S1 Fig Nucleotide coverage. The non-normalised nucleotide coverage displayed as number of nucleotides per position for full genome sequences of the UKDD and DETU virus reads mapped to the corresponding reference sequences. Panel A shows the coverage results for the same SP dataset in the three different DPPs (DPP1: purple; DPP2: orange; DPP3 grey) for each of the SP datasets. Panel B shows the coverage when the same DPP is used to analyse data from the three different SPs (SP1: lilac; SP2: yellow; SP3:green) for each of the DPPs. The X-axis represents the position in the genome, the Y-axis represents the number of sequence reads per position.
(TIF)

S2 Fig Graphical display of the coverage and allele counts for four positions, showing mSNVs in different frequencies for each SP/DPP combination. Arrows indicate the approximate percentages in which the mSNVs were detected; 1–5% (orange), 5–10% (purple) and >10% (green).
(TIF)

## References

[1] Heather J. and Chain B, The sequence of sequencers: The history of sequencing DNA. Genomics, 2016 107(1): p. 1–8. 10.1016/j.ygeno.2015.11.003 26554401 PMC4727787

[2] Van Dijk E, Auger H, Jaszczyszyn Y, Thermes C, Ten years of next-generation sequencing technology. Trends Genet, 2014 30(9): p. 418–26. 10.1016/j.tig.2014.07.001 25108476

[3] Ekblom R. and Galindo J., Applications of next generation sequencing in molecular ecology of non-model organisms. Heredity (Edinb), 2011 107(1): p. 1–15. 21139633 10.1038/hdy.2010.152 PMC3186121

[4] Köser C, Holden M, Ellington M, Cartwright E, Brown N, Ogilvy-Stuart A, et al, Rapid whole-genome sequencing for investigation of a neonatal MRSA outbreak. N Engl J Med, 2012 366(24): p. 2267–75. 10.1056/NEJMoa1109910 22693998 PMC3715836

[5] Mellmann A, Harmsen D, Cummings C, Zentz E, Leopold S, Rico A, et al, Prospective genomic characterization of the German enterohemorrhagic Escherichia coli O104:H4 outbreak by rapid next generation sequencing technology. PLoS One, 2011 6(7): p. e22751 10.1371/journal.pone.0022751 21799941 PMC3140518

[6] Leitner T, Halapi E, Scarlatti G, Rossi P, Albert J, Fenyö E, et al, Analysis of heterogeneous viral populations by direct DNA sequencing. Biotechniques, 1993 15(1): p. 120–7. 8363827

[7] Tsiatis A, Norris-Kirby A, Rich R, Hafez M, Gocke C, Eshleman J, et al, Comparison of Sanger sequencing, pyrosequencing, and melting curve analysis for the detection of KRAS mutations: diagnostic and clinical implications. J Mol Diagn, 2010 12(4): p. 425–32. 10.2353/jmoldx.2010.090188 20431034 PMC2893626

[8] Glenn T. Field guide to next-generation DNA sequencers. Mol Ecol Resour, 2011 11(5): p. 759–69. 10.1111/j.1755-0998.2011.03024.x 21592312

[9] Li Y, Lei K, Kshatriya P, Gu, Jian, Ballesteros-Villagrana, et al., Ion Torrent™ Next Generation Sequencing–Detect 0.1% Low Frequency Somatic Variants and Copy Number Variations simultaneously in Cell-Free DNA. Thermo Fisher Scientific, 2017.

[10] Schirmer M, D’Amore R, Ijaz U, Hall N, Quince C, Illumina error profiles: resolving fine-scale variation in metagenomic sequencing data. BMC Bioinformatics, 2016 17: p. 125 10.1186/s12859-016-0976-y 26968756 PMC4787001

[11] Lou DI, Hussman J, McBee J, Acevedo A, Andino R, Press W, et al, High-throughput DNA sequencing errors are reduced by orders of magnitude using circle sequencing. Proc Natl Acad Sci U S A, 2013 110(49): p. 19872–7. 10.1073/pnas.1319590110 24243955 PMC3856802

[12] World Organisation for Animal Health, O.I.E., Update on highly pathogenic avian influenza in animals (typeH5 and H7). 2014.

[13] World Organisation for Animal Health, O.I.E., Update on highly pathogenic avian influenza in animals (typeH5 and H7). 2015.

[14] Hanna A, Banks J, Marston D, Ellis R, Brookes S, Brown I, Genetic Characterization of Highly Pathogenic Avian Influenza (H5N8) Virus from Domestic Ducks, England, November 2014. Emerg Infect Dis, 2015 21(5): p. 879–82. 10.3201/eid2105.141954 25898126 PMC4412239

[15] Harder T, Maurer-Stroh S, Pohlmann A, Starick E, Höreth-Böntgen D, Albrecht A, et al, Influenza A(H5N8) Virus Similar to Strain in Korea Causing Highly Pathogenic Avian Influenza in Germany. Emerg Infect Dis, 2015 21(5): p. 860–3. 10.3201/eid2105.141897 25897703 PMC4414090

[16] Bouwstra R, Heutink R, Bossers A, Harders F, Koch G, Elbers A, Full-Genome Sequence of Influenza A(H5N8) Virus in Poultry Linked to Sequences of Strains from Asia, the Netherlands, 2014. Emerg Infect Dis, 2015 21(5): p. 872–4. 10.3201/eid2105.141839 25897965 PMC4414092

[17] Verhagen J, Van Der Jeugd H, Nolet B, Slaterus R, Kharitonov S, De Vries P, et al, Wild bird surveillance around outbreaks of highly pathogenic avian influenza A(H5N8) virus in the Netherlands, 2014, within the context of global flyways. Euro Surveill, 2015 20(12). 10.2807/1560-7917.es2015.20.12.21069 25846491

[18] Poen M, Bestebroer T, Vuong O, Scheuer R, Van Der Jeugd H, Kleyheeg E, et al, Local amplification of highly pathogenic avian influenza H5N8 viruses in wild birds in the Netherlands, 2016 to 2017. Euro Surveill, 2018 23(4). 10.2807/1560-7917.ES.2018.23.4.17-00449 PMC5801337 29382414

[19] Global Consortium for, H5N8 and Related Influenza Viruses, Role for migratory wild birds in the global spread of avian influenza H5N8. Science, 2016 354(6309): p. 213–217. 10.1126/science.aaf8852 27738169 PMC5972003

[20] Harrison P, Alako B, Amid C, Cerdeno-Tárraga A, Cleland I, Holt S, et al, The European Nucleotide Archive in 2018. Nucleic Acids Research, 2019 47(D1): p. D84–D88. 10.1093/nar/gky1078 30395270 PMC6323982

[21] Karsch-Mizrachi I, Takagi T, Cochrane G, The international nucleotide sequence database collaboration. Nucleic Acids Research, 2018 46(D1): p. D48–D51. 10.1093/nar/gkx1097 29190397 PMC5753279

[22] Amid C, Pakseresht N, Silvester N, Jayathilaka S, Lund O, Dynocski L, et al., The COMPARE Data Hubs. bioRxiv, 2019: p. 555938. 10.1093/database/baz136 PMC6927095 31868882

[23] Richard M, Herfst S, Van Den Brand J, Lexmond P, Bestebroer T, Rimmelzwaan G, et al, Low Virulence and Lack of Airborne Transmission of the Dutch Highly Pathogenic Avian Influenza Virus H5N8 in Ferrets. PLoS One, 2015 10(6): p. e0129827 10.1371/journal.pone.0129827 26090682 PMC4474857

[24] Linster M, Van Boheemen S, De Graaf M, Schrauwen E, Lexmond P, Mänz B, et al, Identification, characterization, and natural selection of mutations driving airborne transmission of A/H5N1 virus. Cell, 2014 157(2): p. 329–339. 10.1016/j.cell.2014.02.040 24725402 PMC4003409

[25] Li H, Aligning sequence reads, clone sequences and assembly contigs with BWA-MEM. arXiv, 2013.

[26] Li H, Handsaker B, Wysoker A, Fennell T, Ruan J, Homer N, et al, The Sequence Alignment/Map format and SAMtools. Bioinformatics, 2009 25(16): p. 2078–9. 10.1093/bioinformatics/btp352 19505943 PMC2723002

[27] Zerbino D and Birney E, Velvet: algorithms for de novo short read assembly using de Bruijn graphs. Genome Res, 2008 18(5): p. 821–9. 10.1101/gr.074492.107 18349386 PMC2336801

[28] Camacho C, Coulouris G, Avagyan V, Ma N, Papadopoulos J, Bealer K, et al, BLAST+: architecture and applications. BMC Bioinformatics, 2009 10: p. 421 10.1186/1471-2105-10-421 20003500 PMC2803857

[29] Hwang S, Kim E, Lee I, Marcotte E, Systematic comparison of variant calling pipelines using gold standard personal exome variants. Sci Rep, 2015 5: p. 17875 10.1038/srep17875 26639839 PMC4671096

[30] Hall T, BioEdit: a user-friendly biological sequence alignment editor and analysis program for Windows 95/98/NT. Nucleic Acids Symposium Series, 1999 41: p. 95–98.

[31] Dell R, Holleran S, Ramakrishnan R, Sample size determination. ILAR J, 2002 43(4): p. 207–13. 10.1093/ilar.43.4.207 12391396 PMC3275906

[32] Mu W, Lu H, Chen J, Li S, Elliott A, Sanger Confirmation Is Required to Achieve Optimal Sensitivity and Specificity in Next-Generation Sequencing Panel Testing. J Mol Diagn, 2016 18(6): p. 923–932. 10.1016/j.jmoldx.2016.07.006 27720647

[33] Grubaugh N, Gangavarapu K, Quick J, Matteson N, Goes De Jesus J, Main B, et al, An amplicon-based sequencing framework for accurately measuring intrahost virus diversity using PrimalSeq and iVar. Genome Biol, 2019 20(1): p. 8 10.1186/s13059-018-1618-7 30621750 PMC6325816

[34] Golan D and Medvedev P, Using state machines to model the Ion Torrent sequencing process and to improve read error rates. Bioinformatics, 2013 29(13): p. i344–51. 10.1093/bioinformatics/btt212 23813003 PMC3694666

[35] Manley L, Ma D, and Levine S, Monitoring Error Rates In Illumina Sequencing. J Biomol Tech, 2016 27(4): p. 125–128. 10.7171/jbt.16-2704-002 27672352 PMC5026502

[36] Nakamura K, Oshima T, Morimoto T, Ikeda S, Yoshikawa H, Shiwa Y, et al, Sequence-specific error profile of Illumina sequencers. Nucleic Acids Res, 2011 39(13): p. e90 10.1093/nar/gkr344 21576222 PMC3141275

[37] Shao W, Boltz V, Spindler J, Kearney M, Maldarelli F, Mellors J, et al, Analysis of 454 sequencing error rate, error sources, and artifact recombination for detection of Low-frequency drug resistance mutations in HIV-1 DNA. Retrovirology, 2013 10: p. 18 10.1186/1742-4690-10-18 23402264 PMC3599717

[38] Acinas S, Sarma-Rupavtarm R, Klepac-Ceraj V, Poltz M, PCR-induced sequence artifacts and bias: insights from comparison of two 16S rRNA clone libraries constructed from the same sample. Appl Environ Microbiol, 2005 71(12): p. 8966–9. 10.1128/AEM.71.12.8966-8969.2005 16332901 PMC1317340

[39] Gorzer I, Guelly C, Trajanoski S, Puchhammer-Stöckl E, The impact of PCR-generated recombination on diversity estimation of mixed viral populations by deep sequencing. J Virol Methods, 2010 169(1): p. 248–52. 10.1016/j.jviromet.2010.07.040 20691210

[40] Judo M, Wedel A, and Wilson C, Stimulation and suppression of PCR-mediated recombination. Nucleic Acids Res, 1998 26(7): p. 1819–25. 10.1093/nar/26.7.1819 9512558 PMC147471

[41] Meyerhans A, Vartanian J, and Wain-Hobson S, DNA recombination during PCR. Nucleic Acids Res, 1990 18(7): p. 1687–91. 10.1093/nar/18.7.1687 2186361 PMC330584

[42] Quail M, Smith M, Coupland P, Otto T, Harris S, Connor T, et al, A tale of three next generation sequencing platforms: comparison of Ion Torrent, Pacific Biosciences and Illumina MiSeq sequencers. BMC Genomics, 2012 13: p. 341 10.1186/1471-2164-13-341 22827831 PMC3431227

[43] Sims D, Sudbery I, Ilott N, Heger A, Ponting C, Sequencing depth and coverage: key considerations in genomic analyses. Nat Rev Genet, 2014 15(2): p. 121–32. 10.1038/nrg3642 24434847

[44] Liu L, Li Y, Li S, Hu N, He Y, Pong R, et al, Comparison of next-generation sequencing systems. J Biomed Biotechnol, 2012 2012: p. 251364 10.1155/2012/251364 22829749 PMC3398667

[45] Keller M, Rambo-Martin B, Wilson M, Ridenour C, Shepard S, Start T, et al, Direct RNA Sequencing of the Coding Complete Influenza A Virus Genome. Sci Rep, 2018 8(1): p. 14408 10.1038/s41598-018-32615-8 30258076 PMC6158192

