# Supplementary material for: Comparison of sequencing methods and data processing pipelines for whole genome sequencing and minority single nucleotide variant (mSNV) analysis during an influenza A/H5N8 outbreak
Source: PLoS One. 2020 Feb 20;15(2):e0229326. doi: 10.1371/journal.pone.0229326 (PMC7032710; doi:10.1371/journal.pone.0229326)
Supplement: S1 Table — (PDF) [file pone.0229326.s001.pdf]

Gene segment	Set	Sense	Primer Sequence
PB2	1	3-Forward	CGAAAGCAGGTCAAATATATTC
		521-Reverse	TCCATGATGACATCTTGTGCTTC
	2	428-Forward	CATGGAACCTTCGGTCCCGTTCA
		931-Reverse	ATCCACAGCTTGTTCCTCAGTTGG
	3	855-Forward	AGCAACGGTATCAGCGGATCCA
		1403-Reverse	CCATGACATTATCAATGGGTTC
	4	1315-Forward	CCCATGCATCAACTCCTGAGACA
		1820-Reverse	GTTCTCACAAATCCACTGTATTG
	5	1759-Forward	GAACCGTTCCAATCCTTGGTACCT
		2341-Reverse	AGTAGAAACAAGGTCGTTT
PB1	1	3-Forward	RAAAGCAGGCAAACCAYTTGAATG
		538-Reverse	CCATCACATCCTTGAGGAAATC
	2	445-Forward	ACYGCTTTGGCCAACACTATAGA
		944-Reverse	GTATTGTCYCCATGAATTGTAAAGG
	3	877-Forward	GTCCTCAGGAACATGATGACTAACTCAC
		1403-Reverse	ATTCCCTCATGATTCGGTGC
	4	1319-Forward	CCAAAACCACATATTGGTGGGACGG
		1892-Reverse	CTGCCCTGGTARTCTTCATCCATC
	5	1782-Forward	GGCAGGACTGTTGGTTTCAGATGG
		2326-Reverse	TTTTTTCAYGAAGGACAAGC
PA	1	3-Forward	CRAAAGCAGGTACTGATYC
		607-Reverse	CGGATTGACGAAAGGAATCCCA
	2	452-Forward	CACACATTCACATATTCTCATTCAC
		897-Reverse	GCTTAATTTAAGYGCATCCATTCAC
	3	731-Forward	GAGGGCAAGCTTTCTCAAATGTC
		1305-Reverse	TTCATCAAGTTCAATCCAACTGA
	4	1168-Forward	GAGGACTGCAAAGATGTTAGCGA
		1646-Reverse	CAGTACTTTTCCCACTTGTGTGG
	5	1490-Forward	GCAGAACCAAAGAAGGAAGACGG
		2072-Reverse	GATCGAAGGTCCCAGGTTCCAGG
	6	1816-Forward	GCCGAGTCTTCTGTCAAAGAGAA
		2233-Reverse	AGTAGAAACAAGGTACYTTTT
HA	1	5-Forward	AAAGCAGGGGTHYDATCTGTC
		570-Reverse	TTGTARCTYCTCTTTATBGTBGG
	2	465-Forward	GRGTRAGCKCAGCATGTCC
		917-Reverse	GDGTTTGRCACTTGGTGTTGC
	3	803-Forward	AGTAATGGRAATTTCATTGCYCC
		1378-Reverse	ATTYTCCATKAGAACYAGRAGTTC
	4	1247-Forward	ACTCARTTTGARGCHGTTGG

		1789-Reverse	AGTAGAAACAAGGGTGTTTT
NP	1	1-Forward	AGCRAAAGCAGGGTDKATA
		482-Reverse	GCATCATTYAGRTTKGAATGCC
	2	239-Forward	GAATGGTNCTCTCTGCVTTTG
		838-Reverse	TGAGTGCAGACCGHGCCAG
	3	729-Forward	RAAATTYCAAACAGCAGCAC
		1266-Reverse	CTKATYTGYCCTGCVGATGC
	4	1132-Forward	GTTCAAATTGCTTCAAATG
		1565-Reverse	AGTAGAAACAAGGGTATTTT
NA	1	3-Forward	CRAAAGCAGGAGTTYAAAATG
		531-Reverse	GGCTTGATATACATTGGGTGATTG
	2	400-Forward	TGCAGGACTTTCTTCCTCACTCA
		900-Reverse	GTTGTCTCTACACACGCATTCCAC
	3	731-Forward	ATTGGGTAATGACTGACGGTCC
		1237-Reverse	AAGACCCACTGTATCCCGACCA
	4	1103-Forward	GGACAATTAGTCGAACCTCCAGA
		1460-Reverse	AGTAGAAACAAGGAGTTTTT
MA	1	5-Forward	AAAGCAGKTAGATRTTGAAARATG
		564-Reverse	ACCATTCTGTTYTCATGYCTG
	2	461-Forward	TAKTRTGTGCCACTTGTGAGC
		1023-Reverse	AGTAGAAACAAGGTARKTTTT
NS	1	3-Forward	CRAAAGCAGGGTGACAAAVAC
		547-Reverse	CCAATTGCAWTYTTGACATCCTC
	2	453-Forward	AGAGCTTTCACRGAAGAAGGAGCA
		888-Reverse	AGTAGAAMCAAGGGTGTTTT
