# Supplementary material for: Comparison of sequencing methods and data processing pipelines for whole genome sequencing and minority single nucleotide variant (mSNV) analysis during an influenza A/H5N8 outbreak
Source: PLoS One. 2020 Feb 20;15(2):e0229326. doi: 10.1371/journal.pone.0229326 (PMC7032710; doi:10.1371/journal.pone.0229326)
Supplement: S2 Table — (PDF) [file pone.0229326.s002.pdf]

Virus	Segment	Covering positions from start codon ATG	Consensus sequence
NLCH	PB2	1-2280	**ATG**GAGAGAATAAAAGAACTAAGAGATCTAATGTCTCAATCCCGC<br>ACTCGCGAGATACTAACAAAAACCACTGTGGACCATATGGCCATA<br>ATCAAGAAATACACATCAGGAAGACAAGAGAAGAACCCTGCTCTC<br>AGAATGAAATGGATGATGGCAATGAAATATCCAATCACAGCAGAC<br>AAGAGAATAATGGAAATGATTCCTGAAAGAAATGAACAAGGCCA<br>GACGCTTTGGAGCAAGACAAATGATGCTGGATCAGACAGAGTGAT<br>GGTGTCTCCCCTAGCTGTAACTTGGTGGAATAGAAATGGACCGAC<br>AGCAAGTACAGTCCATTATCCAAAGGTCTACAAAACATACTTTGA<br>GAAGGTTGAAAGGTTAAAGCATGGAACCTTCGGTCCCGTTCACTTC<br>CGAAACCAAATTAAAATACGCCGCCGAGTTGACATAAACCCAGGC<br>CACGCAGATCTCAGTGCCAAAGAAGCACAAGATGTCATCATGGAG<br>GTCGTTTTCCCAAATGAAGTGGGAGCTAGAATATTGACATCAGAG<br>TCACAATTGACAATAACGAAAGAGAAAAAAGAAGAACTCCAGGA<br>TTGCAAGATTGCTCCTTTAATGGTGGCATACATGTTGGAAAGAGAA<br>CTGGTCCGCAAAACCAGATTCCTACCAGTAGCAGGTGGGACAAGC<br>AGTGTGTACATTGAGGTACTGCACCTGACCCAAGGGACCTGCTGG<br>GAACAGATGTACACTCCAGGCGGAGAAGTGAGAAATGACGATGTT<br>GACCAGAGTTTGATCATCGCGGCCAGAAACATTGTTAGGAGAGCA<br>ACGGTATCAGCGGATCCACTGGCATCATTATTGGAGATGTGCCAC<br>AGCACACAAATTGGTGGGACAAGGATGGTGGATATCCTTAGGCAA<br>AATCCAACTGAGGAACAAGCTGTGGATATATGCAAAGCAGCAATG<br>GGTTTAAGGATTAGTTCATCCTTTAGCTTTGGAGGATTCACCTTCA<br>AAAGAACTAGTGGTTCATCCATTAGAAAGGAAGAGGAAGTGCTTA<br>CAGGCAACCTCCAAACATTGAAAATAAGAGTACATGAGGGGTAT<br>GAGGAGTTCACAATGGTTGGGCGAAGAGCAACAGCCATTCTAAGG<br>AAAGCAACTAGAAGGCTGATTCAGTTGATAGTAAGTGGAAGAGAC<br>GAACAATCAATCGCTGAAGCAATCATCGTAGCCATGGTGTTCTCAC<br>AGGAGGATTGCATGATAAAGGCAGTCCGAGGCGATCTAAATTTT<br>GTGAACAGAGCAAACCAAAGATTGAACCCCATGCATCAACTCCTG<br>AGACACTTCCAAAAAGATGCAAAAGTGCTGTTTCAAAATTGGGGG<br>ATCGAACCCATTGATAATGTCATGGGGATGATTGGAATATTGCCTG<br>ACATGACTCCAAGCACAGAGATGTCACTAAGAGGAGTAAGAGTT<br>AGTAAAATGGGAGTAGATGAATATTCCAGCACTGAGAGAGTGGTT<br>GTAAGCATTGACCGTTTCTTGCGGGTTCGAGATCAGCAGGGGAAC<br>GTACTCCTATCTCCCGAAGAAGTCAGCGAAACACTGGGAACAGAA<br>AAATTAACAATAACATATTCATCATCAATGATGTGGGAAATCAAT<br>GGTCCTGAGTCAGTGCTGGTCAACACCTATCAATGGATCATCAGA<br>AATTGGGAGATTGTGAAGATTCAATGGTCTCAAGACCCCACGATG<br>CTGTACAATAAGGTGGAGTTTGAACCGTTCCAATCCTTGGTACCTA<br>AAGCTGCCAGAGGCCAATACAGTGGATTTGTGAGAACACTGTTC<br>CAACAAATGCGTGACGTATTGGGGACATTTGATACTATTCAGATA<br>ATAAAGCTGTTACCGTTTGCAGCAGCCCCACCGGAGCATAGCAGA<br>ATGCAATTTTCTTCCCTGACCGTGAATGTAAGGGGCTCGGGAATGA<br>GAATACTCGTAAGGGGTAACTCCCCTGTGTTCAACTACAATAAG<br>GCAACCAAAAGGCTTGCCGTCCTTGGAAAGGACGCAGGTGCATTA<br>ACAGAGGATCCAGATGAGGGGACAACAGGAGTGGAATCTGCAGT<br>GCTGAGGGGGTTCCTAATTCTGGGCAGGGAGGACAGAAGATATGG<br>ACCAGCACTAAGCATCAATGAACTGAGCAATCTTGCGAAAGGGGA<br>GAAAGCCAATGTGCTGATAGGGCAAGGAGACGTGGTGCTGGTAAT<br>GAAACGGAAACGGGACTCTAGCATACTTACTGACAGCCAGACAGC<br>GACCAAAAGAATTCGGATGGTCATCAATTAG
NLCH	PB1	1-2277	**ATG**GATGTCAACCCGACTCTACTCTTCTTGAAAGTGCCAGCGCAA

			AATGCTATAAGTACCACATTCCCCTATACTGGAGATCCTCCATACA<br>GCCATGGAACAGGAACAGGATACACCATGGACACAGTCAACAGA<br>ACGCATCAATACTCAGAAAAGGGAAAGTGGACAAAAAACACCGA<br>GACTGGAGCACCCCAACTCAACCCAATTGATGGACCATTACCTGA<br>GGATAACGAGCCAAGCGGATATGCACAAACGGATTGTGTGTTGGA<br>AGCAATGGCTTTCCTTGAAGAGTCCCACCCAGGGATCTTTGAAAAC<br>TCATGTCTTGAAACAATGGAAATTGTTCAACAAACAAGAGTGGAC<br>AAACTGACCCAAGGTCGTCAGACCTATGACTGGACATTGAATAGA<br>AACCAGCCGGCTGCAACTGCTTTAGCCAACACTATAGAAGTCTTCA<br>GATCGAACGGTCTAACAGCCAATGAGTCAGGGAGACTGATAGATT<br>TCCTCAAAGATGTGATGGAGTCAATGGACAAAGAAGAAATGGAA<br>ATAACAACACATTTCCAAAGAAAGAGAAGAGTAAGAGACAATAT<br>GACCAAGAAAATGGTCACACAAAGAACAATAGGGAAGAAAAAAC<br>AGAGACTGAACAAGAAGAACTACTTGGTAAGGGCACTGACACTGA<br>ACACAATGACAAAAGATGCAGAAAGAGGCAAGTTGAAGAGGCGG<br>GCAATTGCAACACCCGGGATGCAAATCAGAGGGTTCGTGTACTTT<br>GTCGAAACATTAGCGAGGAGCATCTGCGAGAAACTTGAGCAATCT<br>GGGCTCCCTGTTGGAGGAAATGAAAAAAAGGCTAAGTTGGCAAAT<br>GTCGTGAGAAAGATGATGACTAACTCACAAGACACAGAGCTATCC<br>TTTACAATTACTGGAGACAATACCAAGTGGAACGAGAATCAGAAT<br>CCTCGGATTTTTTTGGCAATGATAACATATATCACAAGAAATCAAC<br>CTGAGTGGTTTAGAAATGTGTTAAGTATTGCCCCTATAATGTTCTC<br>AAACAAAATGGCAAGGTTAGGGAAAGGATACATGTTCGAAAGTA<br>AGAGCATGAAGCTACGGACACAAATACCAGCAGAAATGCTTGCAA<br>CCATTGACCTGAAATATTTCAACGAATCGACAAGAAAGAAAATTG<br>AGAAAATAAGGCCTCTCCTAATAGAAGGGACAGCCTCGTTGAGTC<br>CTGGAATGATGATGGGCATGTTCAACATGCTGAGTACAGTCTTGG<br>GAGTATCAATTCTAAATCTTGGCCAAAAGAGGTACACCAAAACCA<br>CATACTGGTGGGACGGACTCCAATCCTCTGATGATTTCGCTCTCAT<br>AGTAAATGCACCGAATCATGAGGGAATACAGGCAGGAGTGGACA<br>GGTTCTATAGGACTTGTAAATTGGTTGGGATCAATATGAGTAAAA<br>AGAAATCCTATATAAATCGGACAGGAACATTTGAATTCACAAGCT<br>TTTTCTACCGTTATGGGTTTGTAGCCAACTTCAGCATGGAGCTGCC<br>CAGCTTTGGAGTTTCTGGGATCAATGAATCGGCTGACATGAGCATT<br>GGAGTTACAGTAATAAAGAATAACATGATAAACAACGATCTTGGA<br>CCAGCAACAGCTCAAATGGCTCTTCAGCTATTTATCAAGGACTACA<br>GATATACATATCGATGCCACAGGGGTGATACACAAATACAAACAA<br>GGAGATCATTCGAGCTAAAGAAGCTGTGGGAGCAGACCCGTTCAA<br>AGGCAGGACTGTTGGTTTCAGATGGAGGCCCAAACTTATACAATA<br>TACGGAATCTCCACATCCCAGAGGTCTGCTTGAAGTGGGAACTGA<br>TGGATGAAGATTACCAGGGTAGACTTTGTAATCCCCTGAACCCCTT<br>TGTCAGTCATAAGGAAATTGAATCCGTAAACAATGCTGTAGTGAT<br>GCCAGCCCATGGTCCGGCCAAAAGCATGGAATATGATGCTGTTGC<br>GACCACACACTCATGGGTCCCTAAGAGGAACCGTTCCATTCTGAAT<br>ACCAGTCAAAGAGGAATCCTTGAGGATGAACAGATGTATCAGAAG<br>TGCTGCAATCTATTTGAAAAATTCTTCCCTAGTAGCTCATACAGGA<br>GGCCAGTTGGAATCTCCAGTATGGTGGAGGCCATGGTGTCTAGGG<br>CCCGAATTGATGCACGGATTGACTTCGAGTCTGGTAGGATTAAGA<br>AGGAAGAGTTTGCTGAGATCATGAAGATCTGTTCCACCATTGAAG<br>AGATCAGACGGCAAAAACAGTGA
NLCH	PA	-6-2190	TCCAAA**ATG**GAAGACTTTGTGCGACAATGCTTCAATCCAATGATC<br>ATCGAGCTTGCGGAAAAGACAATGAAAGAATATGGGGAAAATCC<br>AAAAATCGAAACGAACAAATTCGCTGCAATATGCACTCACTTAGA<br>GGTCTGTTTCATGTATTCGGATTTCCACTTTATTGATGAACGTGGT<br>AAATCAATAATTGTAGAATCTGGCGATCCGAATGCATTATTGAAA<br>CACCGATTTGAGATAATTGAAGGGAGGGACCGAACGATGGCTTGG

			ACAGTGGTAAATAGTATCTGCAACACCACAGGAGTCGATAAGCCT<br>AAATTCCTCCCAGATTTGTATGATTACAAGGAGAACCGATTCATT<br>GAAATTGGAGTGACAAGGAGGGAAGTTCACACATACTACCTAGAA<br>AAGGCAAATAAGATAAAATCAGAGAAGACACACATTCACATATTC<br>TCATTCACTGGGGAGGAGATGGCCACCAAAGCTGATTATATCCTTG<br>ATGAAGAGAGCAGGGCAAGGATCAAAACCAGGTTGTTCACTATC<br>AGGCAAGAAATGGCCAATAGGGGTCTGTGGGATTCCTTTCGTCAA<br>TCTGAGAGAGGCGAAGAGACAATTGAAGAAAGGTTTGAAATCACA<br>GGAACCATGCGCAGGCTTGCCGACCAAAGTCTCCCACCGAATTTCT<br>CCAGCCTTGAAAATTTTAGAGCCTATGTGGATGGATTCAAACCG<br>AACGGCTGCCTTGAGGGCAAGCTTTCTCAAATGTCAAAAGAAGTG<br>AACGCCAGAATTGAGCCATTCATGAAGAAAACACCACGCCCTCTC<br>AGATTACCTGATGGTCCTCCTTGCTCTCAGCGGTCGAAATTCTTAC<br>TGATGGATGCTCTTAAATTGAGCATCGAAGACCCAAGCCATGAG<br>GGAGAAGGTATACCGCTATATGATGCAATCAAATGCATGAAGACG<br>TTTTTTGGTTGGAAAGAGCCCAACATTGTAAAACCACATGTAAAA<br>GGCATAAATCCCAACTATCTCTTGGCTTGGAAGCAGGTGCTGGTAG<br>AACTCCAAGACATTGAAAATGAAGAGAAAATCCCAAAAACAAAA<br>AACATGAAGAAAACAAGCCAACTAAAATGGGCACTCGGTGAGAA<br>TATGGCACCTGAAAAAGTGGACTTTGAGGACTGCAAAGATGTTAG<br>CGATCTAAGACAGTATGACAGTGATGAACCAGAGCCCAGATCATT<br>ATCAAGCTGGATCCAGAGCGAATTCAACAAAGCATGCGAATTGAC<br>AGATTCGAGTTGGATTGAACTTGATGAAATAGGAGAAGATGTTGC<br>TCCAATTGAGCACATTGCGAGTATGAGAAGAAACTACTTCACAGC<br>GGAAGTGTCTCATTGCAGGGCTACTGAATATATAATGAAAGGAGT<br>TTATATAAATACAGCCCTGTTGAATTCATCCTGTGCAGCCATGGAT<br>GACTTCCAATTGATTCCAATGATAAGCAAGTGCAGAACCAAAGAA<br>GGAAGACGGAAGACAAATCTATATGGGTTCATTATAAAAGGAAGA<br>TCCCATTTGAGGAATGATACCGATGTGGTAAATTTTGTGAGCATGG<br>AGTTCTCTCTTACTGACCCGAGGCTGGAACCACACAAGTGGGAA<br>AAGTACTGTGTTCTCGAAATAGGAGACATGCTCCTACGAACTGCA<br>ATAGGCCAAGTATCAAGACCCATGTTTCTTTATGTAAGGACCAATG<br>GGACTTCCAAGATCAAGATGAAATGGGGCATGGAGATGAGGCGAT<br>GCCTTCTTCAATCCCTCCAACAAATTGAGAGCATGATTGAGGCA<br>GAGTCTTCTGTCAAAGAGAAGGACATGACCAAGGAATTCTTTGAA<br>AATAAATCAGAAACGTGGCCAATTGGGGAATCACCTAAGGGGGTG<br>GAGGAAAGCTCTATTGGGAAAGTGTGTAGAACATTACTAGCAAAA<br>TCTGTATTCAACAGCCTATATGCATCTCCACAACTTGAGGGGTTT<br>TCAGCTGAGTCGAGAAAGTTACTTCTCATTGTTCAGGCATTTAGGG<br>ACAACCTGGAACCTGGGACCTTCGATCTTGGGGGGCTATATGAAG<br>CAATTGAGGAGTGCCTGATTAATGATCCCTGGGTTTTGCTTAATGC<br>ATCTTGGTTCAACTCCTTCCTTACACATGCACTGAAATAGTTG<br>TGGCAATGCTACTATTTGCTATCCATACTGTCCAAA
NLCH	HA	7-1704	AAAATAGTGCTTCTTCTTGCAGTGGTTAGCCTTGTTAAAAGTGATC<br>AGATTTGCATTGGTTACCATGCAAACAACTCAACAAAACAGGTTG<br>ACACAATAATGGAAAAAAACGTCACTGTTACACATGCCCAAGACA<br>TACTGGAAAAGACACACAACGGGAAGCTCTGCGATCTTAATGGA<br>GTGAAGCCCCTGATTCTAAAGGATTGTAGCGTAGCTGGGTGGCTCC<br>TTGGAAATCCAATGTGCGACGAGTTCATCAGGGTGCCGGAATGGT<br>CTTACATCGTGGAGAGGGCTAACCCAGCCAACGACCTCTGTTACCC<br>AGGGACCCTCAATGACTATGAGGAACTGAAACACCTACTGAGC<br>AGAATAAATCATTTTGAGAAAACTCTGATCATCCCCAAGAGTTCTT<br>GGCCCAATCATGAAACATCATTAGGGGTGAGCGCAGCATGTCCAT<br>ACCAGGGAGCATCCTCATTTTTCAGAAATGTGGTATGGCTCATCAA<br>AAAGAACGATGCATACCCGACAATAAAGATAAGCTACAATAAT<br>ACCAATCGGGAAGATCTTTTGATACTGTGGGGGATTCATCATCCCA

			ACAATGCAGAAGAGCAGACAAATCTCTATAAAAACCCAGACACTT<br>ATGTTTCCGTTGGGACATCAACATTAAACCAGAGATTGGTGCCAA<br>AAATAGCTACTAGATCCCAAGTAAACGGGCAACGTGGAAGAATG<br>GATTTCTTCTGGACAATTTTAAAACCGAATGATGCAATCCACTTTG<br>AGAGTAATGGAAATTTCATTGCTCCAGAATATGCCTACAAAATTGT<br>CAAGAAAGGGGACTCAACAATTATGAAAAGTGAAGTGGAGTATG<br>GCCACTGCAACACCAAATGTCAAACCCCAATAGGGGCGATAAAC<br>TCTAGCATGCCATTCCACAATATACACCCTCTCACCATCGGGGAAT<br>GCCCCAAATACGTGAAGTCAAACAAATTAGTCCTTGCGACTGGGC<br>TCAGAAATAGTCCTCTAAGGGAAAGAAGAAGAAAAAGAGGACTA<br>TTTGGAGCTATAGCAGGGTTTATAGAGGGAGGATGGCAGGGAATG<br>GTAGACGGTTGGTATGGGTACCACCATAGCAATGAGCAGGGGAGT<br>GGGTACGCTGCAGACAAAGAATCCACCCAAAAGGCAGTAGATGG<br>AGTTACCAATAAGGTCAACTCAATCATTGACAAAATGAACACTCA<br>ATTTGAGGCCGTTGGAAGGGAATTTAATAATTTAGAAAGGAGAAT<br>AGAGAATCTAAACAAGAAAATGGAAGACGGATTCCTAGATGTCTG<br>GACTTATAATGCTGAACTTTTAGTTCTCATGGAAAATGAGAGAACT<br>CTAGATTTCCATGACTCAAATGTCAAGAACCTTTACGACAAAGTCC<br>GACTACAGCTTAGGGATAATGCAAAGGAGCTGGGTAATGGTTGT<br>TTCGAGTTCTATCACAAATGTGATAACGAATGTATGGGAAGCGTA<br>AGAAATGGGACGTATGACTACCCTAAGTATTCAGAAGAAGCAAGG<br>TTAAAAAGAGAAGAAATAAGCGGAGTGAAATTAGAATCAATAGG<br>AACTTACCAAATACTGTCAATTTATTCAACAGTGGCGAGTTCCCTA<br>GCACTGGCAATCATAGTGGCTGGTCTATCTTTATGGATGTGCTCTA<br>ATGGGTCGCTACAATGCAGAATTTGCATCTAA
NLCH	NP	1-1497	**ATG**GCGTCTCAAGGCACCAAACGATCTTATGAACAGATGGAAACT<br>GGTGGAGAACGCCAGAATGCCACTGAAATCAGAGCATCTGTTGGA<br>AGAATGGTTGGTGGAATTGGAAGGTTTTATATACAGATGTGCACT<br>GAACTCAAACTCAGCAATTATGAGGGGAGACTGATCCAGAACAGC<br>ATAACAATAGAAAGAATGGTTCTCTCTGCATTTGATGAAAGGAGG<br>AACAAGTACCTGGAAGAACATCCCAGTGCGGGGAAGGACCCAAA<br>GAAAACTGGAGGTCCAATCTACAGAAGAAGAGACGGAAAGTGGA<br>TGAGGGAGCTGATTCTGTATGACAAAGAAGAGATCAGAAGGATCT<br>GGCGTCAAGCAAATAATGGAGAAGATGCAACTGCTGGTCTCACCC<br>ATCTGATGATCTGGCACTCCAACCTGAATGATGCCACATATCAGAG<br>GACAAGGGCTCTCGTGCGCACTGGAATGGATCCCAGAATGTGCTC<br>TCTGATGCAAGGATCAACTCTCCCAAGAAGGTCTGGAGCTGCTGG<br>TGCAGCAGTAAAAGGGGTCGGAACAATGGTAATGGAATTGATTCG<br>AATGATAAAGCGAGGGATTAATGATCGGAATTTCTGGAGAGGCGA<br>AAATGGAAGAAGGACAAGGATTGCCTATGAGAGAATGTGCAACAT<br>CCTCAAAGGGAAATTTCAAACAGCAGCACAAAGAGCAATGATGGA<br>TCAAGTGCGAGAAAGCAGGAATCCTGGGAATGCTGAAATTGAAGA<br>TCTCATTTTTCTGGCACGGTCTGCACTCATCCTGAGAGGATCAGTG<br>GCCCACAAGTCTTGTCTGCCTGCTTGTGTTTACGGACTTGCTGTGG<br>CCAGTGGATATGACTTTGAGAGAGAAGGATACTCTCTGGTTGGA<br>ATAGACCCTTTCCGTCTGCTTCAAAACAGCCAGGTCTTCAGTCTCA<br>TTAGACCAAATGAAAACCCAGCACATAAAAGTCAGTTGGTATGGA<br>TGGCATGCCATTCAGCAGCGTTTGAGGACCTGAGAGTATCAAGTTT<br>CATCAGAGGGACAAGAGTGGTCCCAAGAGGACAACTATCCACCAG<br>AGGAGTTCAAATTGCATCAAATGAAAACATGGAAACAATGGACTC<br>CAGCACTCTTGAATTGAGAAGCAGATACTGGGCTATAAGAACCAG<br>GAGTGGAGGAAACACCAACCAACAGAGAGCTTCTGCAGGACAAA<br>TCAGCGTACAACCCACCTTCTCAGTACAGAGAAATCTTCCCTTT<br>GAAAGAACGACCATCATGGCGGCATTTACAGGGAACACTGAAGGC<br>AGGACCTCTGACATGAGGACTGAGATCATAAGAATGATGGAAAGT<br>GCCAAACCAGAAGATGTGTCCTTCCAGGGGCGGGGAGTCTTCGAG

			CTCTCGGACGAAAAGGCAACGAACCCGATCGTGCCTTCCTTTGAC<br>ATGAGCAACGAAGGATCTTATTTCTTCGGAGACAGTGCAGAGGAG<br>TATGACAATTAA
NLCH	NA	4-1419	AATCCAAATCAGAAAATAGTAACCATTGGCTCCATTTCATTAGGGT<br>TGGTTGTATTCAATGTTCTACTGCATGCTGTGAGCATCATATTAAC<br>AGTGTTAGCCCTGGGGAAGAGTGAAAACAATGGAATCTGCAATGG<br>AACTGTAGTGAGAGAATACAATGAAACAGTTAGAATAGAGAAA<br>GTGACTCAATGGTACAATACTAGCGTAGTCGAATATGTACCGCATT<br>GGAATGAGGGCACTTATATAAATAACACCGAACCAATATGTGATG<br>TCAAGGGCTTTGCACCTTTTTCCAAGGACAACGGGATAAGAGTTG<br>GCTCCAGGGGACATATTTTTGTCATAAGAGAGCCTTTCGTCTCT<br>TGTTCACCTGTAGAGTGCAGGACTTTCTTCCTCACTCAGGGATCTC<br>TACTCAATGACAAACACTCAAATGGAACAATGAAGGATAGAAGCC<br>CATTCAGAACTCTCATGAGTGTCGAAGTGGGCCAATCACCCAATGT<br>ATATCAAGCCAGGTTTGAAGCTGTGGCATGGTCAGCAACAGCC<br>TGTCATGATGGTAAGAAGTGGATGACGATTGGTGTAACAGGGCCA<br>GATTCTAAAGCAGTAGCAGTAGTTCATTACGGAGGGGTGCCTACT<br>GACGTTGTTAACTCCTGGGCAGGAGATATATTAAGAACTCAGGAG<br>TCATCTTGTACTTGCATTCAAGGTAATTGTTATTGGGTAATGACT<br>GACGGTCCTGCCAATAGACAGGCGCAGTATAGAATATACAAAGCA<br>AATCAAGGCAAAATAATTGGCCGAACAGATGTTAGCTTTAGTGGA<br>GGACATATTGAGGAATGTTCTTGTTATCCAAATGATGGTAAAGTGG<br>AATGCGTGTGTAGAGACAACTGGACGGGAACTAACAGGCCTGTA<br>CTAATTATTTCGCCTGATCTCTCTTACAGGGTTGGGTATTTATGTGC<br>AGGGTTGCCCAGTGACACTCCAAGAGGGGAAGATACTCAATTTGT<br>CGGTTCATGCACTAGTCCCATGGGAAATCAGGGATATGGCGTAAA<br>AGGGTTCGGGTTTCGACAGGGAACTGATGTGTGGGTGGGGCGG<br>ACAATTAGTCGAACCTCCAGATCAGGATTTGAAATAATAAGGATA<br>AAGAATGGTTGGACGCAAACAAGCAAAGAACAGATTAGAAGACA<br>GGTGGTTGTTGATAACTCGAATTGGTCGGGATACAGTGGGTCTTTC<br>ACTTTACCAGTAGAATTGTCTGGGAGGGAATGTTTGGTTCCCTGT<br>TTTTGGGTCGAAATGATCAGAGGTAGGCCAGAAGAGAGAACAATC<br>TGGACCTCTAGTAGCTCCATTGTAATGTGTGGAGTTGATTATGAAA<br>TTGCCGATTGGTCATGGCACGATGGAGCTATTCTTCCCTTTGACAT<br>CGATAAGACGTAATTTACG
NLCH	MP	-5-982	GAAAG**ATG**AGTCTTCTAACCGAGGTCGAAACGTACGTTCTCTCTAT<br>CATCCCGTCAGGCCCCCTCAAAGCCGAGATCGCGCAGAGACTTGA<br>AGATGTCTTTGCAGGGAAAAACACCGATCTCGAGGCTCTCATGGA<br>GTGGCTAAAGACAAGACCAATCCTGTCACCTCTGACTAAAGGGA<br>TTTTGGGATTTGTGTTCACGCTCACCGTGCCCAGTGAGCGAGGACT<br>GCAGCGTAGACGCTTCGTCCAGAATGCCCTAAATGGAAACGGGGA<br>TCCAAATAATATGGATAAGGCAGTTAAGCTATATAAGAAGCTGAA<br>AAGAGAGATAACATTCCATGGGGCTAAGGAGGTCGCACTTAGCT<br>ACTCAACCGGTGCACTTGCCAGCTGCATGGGTCTCATATACAACAG<br>GATGGGAACGGTGACTACAGAAGTGGCTTTTGGCCTAGTGTGTGC<br>CACTTGTGAGCAGATTGCAGATTCACAGCATCGGTCCCACAGACA<br>GATGGCAACCATCACCAACCCATTAATCAGACATGAGAACAGAA<br>TGGTGCTGGCCAGCACTACAGCTAAGGCCATGGAGCAGATGGCAG<br>GATCAAGCGAGCAGGCATCAGAAGCCATGGAGGTTGCTAATCAGG<br>CCAGGCAGATGGTACAGGCAATGAGGACAATTGGGACTCATCCTA<br>ACTCTAGTGCTGGTCTGAGAGATAATCTTCTTGAAAATTTGCAGG<br>CCTACCAGAACCGAATGGGAGTGCAGATGCAGCGATTCAAGTGAT<br>CCTCTTGTTGTTGCCGCAAATATCATTGGGATCCTGCACTTGATATT<br>GTGGATCCTTGATCGTCTTTTCTTCAAATGCATTTATCGTCGCCTTA<br>AATACGGTTTGAAAATAGGGCCTTCTACGGAAGGGGTACCT<br>GAGTCTATGAGGGAAGAGTACCGGCAGGAACAGCAGAGTGCTGTG

			GATGTTGACGATGGTCATTTTGTCAACATAGAATTGGAGTAA
NLCH	NS	1-838	**ATG**GACTCCAACACTGTGTCAAGCTTTCAGGTAGACTGCTTTCTTT<br>GGCATGTCCGCAAACGATTTGCAGACCAAGAACTGGGTGATGCCC<br>CATTCCTTGACCGGCTTCGCCGAGACCAGAAGTCCCTAAGAGGAA<br>GAGGCAGCACTCTTGGTCTGGACATCGAGACAGCTACTCGTGCG<br>GGAAAGCAAATATTGGAGCGGATTCTGGGGGAAGAATCTGATGAA<br>GCACTTAAAATGAATATTGCTTCTGTACCGACTTCACGCTACCTAA<br>CTGACATGACTCTTGAAGAAATGTCAAGAGACTGGTTCATGCTCAT<br>GCCCAAGCAGAAAGTAGCAGGTTCTCTCTGCATCAAAATGGACCA<br>GGCAATAATGGATAAAACCATCATACTGAAAGCAAACTTCAGTGT<br>GATTTTTGATCGGCTGGAAACCCTAATATTACTTAGAGCTTTCACA<br>GAAGAAGGAGCAATTGTGGGAGAAATCTCACCATTACCTTCTCTTC<br>CAGGACATACTGATGAGGATGTCAAAATTGCAATTGGGGTCCTCA<br>TCGGAGGGCTTGAATGGAATGATAACACAGTTCGAGTCTCTGAAA<br>CTCTACAGAGATTCACTTGGAGAAGCAGTAATGAGGATGGGAGAC<br>CTTCACTCCCTTCAAAACAGAAACGGAAAATGGCGAGAACAATTG<br>AGTCAGAAGTTCGAGGAAATAAGATGGCTGATTGAGGAAATGCGA<br>CATAGATTGAAGATCACAGAGAACAGCTTCGAACAAATAACGTTT<br>ATGCAAGCTTTACAACTATTGCTTGAAGTGGAGCAAGAGATAAGA<br>ACCTTCTCGTTTCAGCTTATTTAA
DETU	PB2	1-2287	**ATG**GAGAGAATAAAAGAACTAAGAGATCTAATGTCTCAATCCCGC<br>ACTCGCGAGATACTAACAAAAACCACTGTGGACCATATGGCCATA<br>ATCAAGAAATACACATCAGGAAGACAAGAGAAGAACCCTGCTCTC<br>AGAATGAAATGGATGATGGCAATGAAATATCCAATCACAGCAGAC<br>AAGAGAATAATGGAAATGATTCCTGAAAGAAATGAACAAGGCCA<br>GACGCTTTGGAGCAAGACAAATGATGCTGGATCAGACAGAGTGAT<br>GGTGTCTCCCCTAGCTGTAACTTGGTGGAATAGAAATGGACCGAC<br>AGCAAGTACAGTCCATTATCCAAAGGTCTACAAAACATACTTTGA<br>GAAGGTTGAAAGGTTAAAGCATGGAACCTTCGGTCCCGTTCACTTC<br>CGAAACCAAATTAAAATACGCCGCCGAGTTGACATAAACCCAGGC<br>CACGCAGATCTCAGTGCCAAAGAAGCACAAGATGTCATCATGGAG<br>GTCGTTTTCCCAAATGAAGTGGGAGCTAGAATATTGACATCAGAG<br>TCACAATTGACAATAACGAAAGAGAAAAAAGAAGAACTCCAGGA<br>TTGCAAGATTGCTCCTTTAATGGTGGCATACATGTTGGAAAGAGAA<br>CTGGTCCGCAAAACCAGATTCCTACCAGTAGCAGGTGGGACAAGC<br>AGTGTGTACATTGAGGTACTGCACCTGACCCAAGGGACCTGCTGG<br>GAACAGATGTACACTCCAGGCGGAGAAGTGAGAAATGACGATGTT<br>GACCAGAGTTTGATCATCGCGGCCAGAAACATTGTTAGGAGAGCA<br>ACGGTATCAGCGGATCCACTGGCATCATTATTGGAGATGTGCCAC<br>AGCACACAAATTGGTGGGACAAGGATGGTGGATATCCTTAGGCAA<br>AATCCAACTGAGGAACAAGCTGTGGATATATGCAAAGCAGCAATG<br>GGTTTAAGGATTAGTTCATCCTTTAGCTTTGGAGGATTCACCTTCA<br>AAAGAACAAGTGGTTCATCCATTAGAAAGGAAGAGGAAGTGCTTA<br>CAGGCAACCTCCAAACATTGAAAATAAGAGTACATGAGGGGTAT<br>GAGGAGTTCACAATGGTTGGGCGAAGAGCAACAGCCATTCTAAGG<br>AAAGCAACTAGAAGGCTGATTCAGTTGATAGTAAGTGGAAGAGAC<br>GAACAATCAATCGCTGAAGCAATCATCGTAGCCATGGTGTTCTCAC<br>AGGAGGATTGCATGATAAAGGCAGTCCGAGGCGATCTAAATTTT<br>GTGAACAGAGCAAACCAAAGATTGAACCCCATGCATCAACTCCTG<br>AGACACTTCCAAAAAGATGCAAAAGTGCTGTTTCAAAATTGGGGG<br>ATCGAACCCATTGATAATGTCATGGGGATGATTGGAATATTGCCTG<br>ACATGACTCCAAGCACAGAGATGTCACTAAGAGGAGTAAGAGTT<br>AGTAAAATGGGAGTAGATGAATATTCCAGCACTGAGAGAGTGGTT<br>GTAAGCATTGACCGTTTCTTGCGGGTTCGAGATCAGCAGGGGAAC<br>GTACTCCTATCTCCCGAAGAAGTCAGCGAAACACTGGGAACAGAA<br>AAATTAACAATAACATATTCATCATCAATGATGTGGGAAATCAAT

			GGTCCTGAGTCAGTGCTGGTCAACACCTATCAATGGATCATCAGA AATTGGGAGATTGTGAAGATTCAATGGTCTCAAGACCCCACGATG CTGTACAATAAGGTGGAGTTTGAACCGTTCCAATCCTTGGTACCTA AAGCTGCCAGAGGCCAATACAGTGGATTTGTGAGAACACTGTTC CAACAAATGCGTGACGTATTGGGGACATTTGATACTATTCAGATA ATAAAGCTGTTACCGTTTGCAGCAGCCCCACCGGAGCATAGCAGA ATGCAATTTTCTTCCCTGACCGTGAACGTAAGAGGCTCGGGAATGA GAATACTCGTAAGGGGTAACTCCCCTGTGTTCAACTACAATAAG GCAACCAAAAGGCTTGCCGTCCTTGGAAAGGACGCAGGTGCATTA ACAGAGGATCCAGATGAGGGGACAACAGGAGTGGAATCTGCAGT GCTGAGGGGGTTCCTAATTCTGGGCAGGGAGGACAGAAGATATGG ACCAGCACTAAGCATCAATGAACTGAGCAATCTTGCGAAAGGGGA GAAAGCCAATGTGCTGATAGGGCAAGGAGACGTGGTGCTGGTAAT GAAACGGAAACGGGACTCTAGCATACTTACTGACAGCCAGACAGC GACCAAAAGAATTCGGATGGTCATCAATTAGTATCGAG
DETU	PB1	1-2277	**ATG**GATGTCAACCCGACTTTACTCTTCTTGAAAGTGCCAGCGCAAA ATGCTATAAGTACCACATTCCCTTATACTGGAGATCCTCCATACAG CCATGGAACAGGAACAGGATACACCATGGACACAGTCAACAGAA CGCATCAATACTCAGAAAAGGGAAAGTGGACAAAAAACACCGAG ACTGGAGCACCCCAACTCAACCCAATTGATGGACCATTACCTGAG GATAACGAGCCAAGCGGATATGCACAAACGGATTGTGTGTTGGAA GCAATGGCTTTCCTTGAAGAGTCCCACCCAGGGATCTTTGAAAACT CATGTCTTGAAACAATGGAAATTGTTCAACAAACAAGAGTGGAC AAACTGACCCAAGGTCGTCAGACCTATGACTGGACATTGAATAGA AACCAGCCGGCTGCAACTGCTTTAGCCAACACTATAGAAGTCTTCA GATCGAACGGTCTAACAGCCAATGAGTCAGGGAGACTGATAGATT TCCTCAAAGATGTGATGGAGTCAATGGACAAAGAAGAAATGGAA ATAACAACACATTTCCAAAGAAAGAGAAGAGTAAGAGACAATAT GACCAAGAAAATGGTCACACAAAGAACAATAGGGAAGAAAAAAC AGAGACTGAACAAGAAGAACTACTTGGTAAGGGCACTGACACTGA ACACAATGACAAAAGATGCAGAAAGAGGCAAGTTGAAGAGGCGG GCAATTGCAACACCCGGGATGCAAATCAGAGGGTTCGTGTACTTT GTCGAAACATTAGCGAGGAGCATCTGCGAGAAACTTGAGCAATCT GGGCTCCCTGTTGGAGGAAATGAAAAAAAGGCTAAGTTGGCAAAT GTCGTGAGAAAGATGATGACTAACTCACAAGACACAGAGCTATCC TTTACAATTACTGGAGACAATACCAAGTGGAACGAGAATCAGAAT CCTCGGATTTTTTTGGCAATGATAACATATATCACAAGAAATCAAC CTGAGTGGTTTAGAAATGTGTTAAGTATTGCCCCTATAATGTTCTC AAACAAAATGGCAAGATTAGGGAAAGGATACATGTTCGAAAGTA AGAGCATGAAGCTACGGACACAAATACCAGCAGAAATGCTTGCAA CCATTGACCTGAAATATTTCAACGAATCGACAAGAAAGAAAATTG AGAAAATAAGGCCTCTCCTAATAGAAGGGACAGCCTCGTTGAGTC CTGGAATGATGATGGGCATGTTCAACATGCTGAGTACAGTCTTGG GAGTATCAATTCTAAATCTTGGCCAAAAGAGGTACACCAAAACCA CATACTGGTGGGACGGACTCCAATCCTCTGATGATTTCGCTCTCAT AGTAAATGCACCGAATCATGAGGGAATACAGGCAGGAGTGGACA GGTTCTATAGGACTTGTAAATTGGTTGGGATCAATATGAGTAAAA AGAAATCCTATATAAATCGGACAGGAACATTTGAATTCACAAGCT TTTTCTACCGTTATGGGTTTGTAGCCAACTTCAGCATGGAGCTGCC CAGCTTTGGAGTTTCTGGGATTAATGAATCGGCTGACATGAGCATT GGAGTTACAGTAATAAAGAATAACATGATAAACAACGATCTTGGA CCAGCAACAGCTCAAATGGCTCTTCAGCTATTTATCAAGGACTACA GATATACATATCGATGCCACAGGGGTGATACACAAATACAAACAA GGAGATCATTCGAGCTAAAGAAGCTGTGGGAGCAGACCCGTTCAA AGGCAGGACTGTTGGTTTCAGATGGAGGCCCAAACTTATACAAT ATACGGAATCTCCACATCCCAGAGGTCTGCTTGAAGTGGGAACTG

			ATGGATGAAGATTACCAGGGTAGACTTTGTAATCCCCTGAACCCCT<br>TTGTCAGTCATAAGGAAATTGAATCCGTAAACAATGCTGTAGTGAT<br>GCCAGCCCATGGTCCGGCCAAAAGCATGGAATATGATGCTGTT<br>GCGACCACACACTCATGGGTCCCTAAGAGGAACCGTTCCATTCTG<br>AATACCAGTCAAAGAGGAATCCTTGAGGATGAACAGATGTATCAG<br>AAGTGCTGCAATCTATTTGAAAAATTCTTCCCTAGTAGCTCATACA<br>GGAGGCCAGTTGGAATCTCCAGTATGGTGGAGGCCATGGTGTCT<br>AGGGCCCGAATTGATGCACGGATTGACTTCGAGTCTGGTAGGATT<br>AAGAAGGAAGAGTTTGCTGAGATCATGAAGATCTGTTCCACCATT<br>GAAGAGATCAGACGGCAAAAACAGTGA
DETU	PA	7-2190	GACTTTGTGCGACAATGCTTCAATCCAATGATCGTCGAGCTTGCGG<br>AAAAGACAATGAAAGAATATGGGGAAAATCCAAAAATCGAAACG<br>AACAAATTCGCTGCAATATGCACTCACTTAGAGGTCTGTTTCATGT<br>ATTCGGATTTCCACTTTATTGATGAACGAGGTAAATCAATAATTGT<br>AGAATCTGGCGATCCGAATGCATTATTGAAACACCGATTTGAGAT<br>AATTGAAGGGAGGGACCGAACGATGGCTTGGACAGTGGTAAATAG<br>TATCTGCAACACCACAGGAGTCGATAAGCCTAAATTCCTCCCAGAT<br>TTGTATGATTACAAGGAGAACCGATTCATTGAAATTGGAGTGACA<br>AGGAGGGAAGTTCACACATACTACCTAGAAAAGGCAAATAAGATA<br>AAATCAGAGAAGACACACATTCACATATTCTCATTCACTGGGGAG<br>GAGATGGCCACCAAAGCTGATTATATCCTTGATGAAGAGAGCAGA<br>GCAAGGATCAAAACCAGGTTGTTCACTATCAGGCAAGAAATGGCC<br>AATAGGGGTCTGTGGGATTCCTTTCGTCAATCTGAGAGAGGCGAA<br>GAGACAATTGAAGAAAGGTTTGAAATCACAGGAACCATGCGCAGG<br>CTTGCCGACCAAAGTCTCCCACCGAATTTCTCCAGCCTTGAAAATT<br>TTAGAGCCTATGTGGATGGATTCAAACCGAACGGCTGCCTTGAGG<br>GCAAGCTTTCTCAAATGTCAAAAGAAGTGAACGCCAGAATTGAGC<br>CATTCATGAAGACAACACCACGCCCTCTCAGATTACCTGATGGTCC<br>TCCTTGCTCTCAGCGGTCGAAATTCTTACTGATGGATGCTCTTAAA<br>TTGAGCATCGAAGACCCAAGCCATGAGGGAGAAGGTATACCGCTA<br>TATGATGCAATCAAATGCATGAAGACGTTTTTTGGTTGGAAAGAG<br>CCCAACATTGTAAAACCACATGTAAAAGGCATAAATCCCAACTAT<br>CTCTTGGCTTGGAAGCAGGTGCTGGTAGAACTCCAAGACATTGAA<br>AATGAAGAGAAAATCCCAAAAACAAAAAACATGAAGAAAACAAG<br>CCAACTAAAGTGGGCACTCGGTGAGAATATGGCACCTGAAAAAGT<br>GGACTTTGAGGACTGCAAAGATGTTAGCGATCTAAGACAGTATGA<br>CAGTGATGAACCAGAGCCCAGATCATTATCAAGCTGGATCCAGAG<br>CGAATTCAACAAAGCATGCGAATTGACAGATTCGAGTTGGATTGA<br>ACTTGATGAAATAGGAGAAGATGTTGCTCCAATTGAGCACATTGC<br>GAGTATGAGAAGAAACTACTTCACAGCGGAAGTGTCTCATTGCAG<br>GGCTACTGAATATATAATGAAAGGAGTTTATATAAATACAGCCCT<br>GTTGAATTCATCCTGTGCAGCCATGGATGACTTCCAATTGATTCCA<br>ATGATAAGCAAGTGCAGAACCAAAGAAGGAAGACGGAAGACAAA<br>TCTATATGGGTTCATTATAAAAGGAAGATCCCATTTGAGGAATGAT<br>ACCGATGTGGTAAATTTTGTGAGCATGGAGTTCTCTCTTACTGACC<br>CGAGGCTGGAACCACACAAGTGGGAAAAGTACTGTGTTCTCGAAA<br>TAGGAGACATGCTCCTACGAACTGCAATAGGCCAAGTATCAAGAC<br>CCATGTTTCTTTATGTAAGGACCAATGGGACTTCCAAGATCAAGAT<br>GAAATGGGGCATGGAGATGAGGCGATGCCTTCTTCAATCCCTCCA<br>ACAAATTGAGAGCATGATTGAGGCAGAATCTTCTGTCAAAGAGAA<br>GGACATGACCAAGGAATTCTTTGAAAATAAATCAGAAACGTGGCC<br>AATTGGGGAATCACCTAAGGGGGTGGAGGAAAGCTCTATTGGGAA<br>AGTGTGTAGAACATTACTAGCAAAATCTGTATTCAACAGCCTATAT<br>GCATCTCCACAACTTGAGGGGTTTTCAGCTGAGTCGAGAAAGTTAC<br>TTCTCATTGTTCAGGCATTTAGGGACAACCTGGAACCTGGGACCTT<br>CGATCTTGGGGGGCTATATGAAGCAATTGAGGAGTGCCTGATTAA

			TGATCCCTGGGTTTTGCTTAATGCATCTTGGTTCAACTCCTTCCTTA<br>CACATGCACTGAAATAGTTGTGGCAATGCTACTATTTGCTATCCAT<br>ACTGTCCAA
DETU	HA	1-1728	**ATG**GAGAAAATAGTGCTTCTTCTTGCAGTGGTTAGCCTTGTTAAAA<br>GTGATCAGATTTGCATTGGTTACCATGCAAACAACTCAACAAAAC<br>AGGTTGACACAATAATGGAAAAAAACGTCACTGTTACACATGCCC<br>AAGACATACTGGAAAAGACACACAACGGGAAGCTCTGCGATCTTA<br>ATGGAGTGAAGCCCCTGATTCTAAAGGATTGTAGCGTAGCTGGGT<br>GGCTCCTTGGAAATCCAATGTGCGACGAGTTTATCAGGGTGCCGG<br>AATGGTCTTACATCGTGGAGAGGGCTAACCCAGCCAACGACCTCT<br>GTTACCCAGGGACCCTCAATGACTATGAGGAACTGAAACACCTA<br>CTGAGCAGAATAAATCATTTTGAGAAAACTCTGATCATCCCCAAG<br>AGTTCTTGGCCCAATCATGAAACATCATTAGGGGTGAGCGCAGCA<br>TGTCCATACCAGGGAGCATCCTCATTTTTCAGAAATGTGGTATGGC<br>TCATCAAAAAGAACGATGCATACCCGACAATAAAGATAAGCTAC<br>AATAATACCAATCGGGAAGATCTTTTGATACTGTGGGGGATTCATC<br>ATCCCAACAATGCAGAAGAGCAGACAAATCTCTATAAAAACCCAG<br>ACACTTATGTTTCCGTTGGGACATCAACATTAAACCAGAGATTGGT<br>GCCAAAAATAGCTACTAGATCCCAAGTAAACGGGCAACGTGGAAG<br>AATGGATTTCTTCTGGACAATTTTAAAACCGAATGATGCAATCCAC<br>TTTGAGAGTAATGGAAATTTCATTGCTCCAGAATATGCCTACAAAA<br>TTGTCAAGAAAGGGGACTCAACAATTATGAAAAGTGAAGTGGAGT<br>ATGGCCACTGCAACACCAAATGTCAAACCCCAATAGGGGCGATAA<br>ACTCTAGCATGCCATTCCACAATATACACCCTCTCACCATCGGGGA<br>ATGCCCCAAATATGTGAAGTCAAACAAATTAGTCCTTGCGACTGG<br>GCTCAGAAATAGTCCTCTAAGGGAAAGAAGAAGAAAAAGAGGAC<br>TATTTGGAGCTATAGCAGGGTTTATAGAGGGAGGATGGCAGGGAA<br>TGGTAGACGGTTGGTATGGGTACCACCATAGCAATGAGCAGGGGA<br>GTGGGTACGCTGCAGACAAAGAATCCACCCAAAAGGCAGTAGATG<br>GAGTTACCAATAAGGTCAACTCAATCATTGACAAAATGAACACTC<br>AATTTGAGGCCGTTGGAAGGGAATTTAATAACTTAGAAAGGAGAA<br>TAGAGAATTTAAACAAGAAAATGGAAGACGGATTCCTAGATGTCT<br>GGACTTATAATGCTGAACTTTTAGTTCTTATGGAAAATGAGAGAAC<br>TCTAGATTTCCATGACTCAAATGTCAAGAACCTTTACGACAAAGTC<br>CGACTACAGCTTAGGGATAATGCAAAGGAGCTGGGTAATGGTTGT<br>TTCGAGTTCTATCACAAATGTGATAACGAATGTATGGAAAGCGTA<br>AGAAATGGGACGTATGACTACCCTAAGTATTCAGAAGAAGCAAGA<br>TTAAAAAGAGAAGAAATAAGCGGAGTGAAATTAGAATCAATAGG<br>AACTTACCAAATACTGTCAATTTATTCAACAGTGGCGAGTTCCCTA<br>GCACTGGCAATCATAGTGGCTGGTCTATCTTTATGGATGTGCTCTA<br>ATGGGTCGCTACAATGCAGAATTTGCATCTAAATTTGTGAGCTCAG<br>ATTGTAATTA
DETU	NP	1-1497	**ATG**GCGTCTCAAGGCACCAAACGATCTTATGAACAGATGGAAACT<br>GGTGGAGAACGCCAGAATGCCACTGAAATCAGAGCATCTGTTGGA<br>AGAATGGTTGGTGGAATTGGAAGGTTTTATATACAGATGTGCACT<br>GAACTCAAACTCAGCAATTATGAGGGGAGACTGATCCAGAACAGC<br>ATAACAATAGAAAGAATGGTTCTCTCTGCATTTGATGAAAGGAGG<br>AACAAGTACCTGGAAGAACATCCCAGTGCGGGGAAGGACCCAAA<br>GAAAACTGGAGGTCCAATCTACAGAAGAAGAGACGGAAAGTGGA<br>TGAGGGAGCTGATTCTGTATGACAAAGAAGAGATCAGAAGGATCT<br>GGCGTCAAGCAAATAATGGAGAAGATGCAACTGCTGGTCTCACCC<br>ATCTGATGATCTGGCACTCCAACCTGAATGATGCCACATATCAGAG<br>GACAAGGGCTCTCGTGCGCACTGGAATGGATCCCAGAATGTGCTC<br>TCTGATGCAAGGATCAACTCTCCCAAGAAGGTCTGGAGCTGCTGG<br>TGCAGCAGTAAAAGGGGTCGGAACAATGGTAATGGAATTGATTCG<br>AATGATAAAGCGAGGGATTAATGATCGGAATTTCTGGAGAGGCGA

			AAATGGAAGAAGGACAAGGATTGCCTATGAGAGAATGTGCAACAT<br>CCTCAAAGGGAAATTTCAAACAGCAGCACAAAGAGCAATGATGGA<br>TCAAGTGCGAGAAAGCAGGAATCCTGGGAATGCTGAAATTGAAGA<br>TCTCATTTTTCTGGCACGGTCTGCACTCATCCTGAGAGGATCAGTG<br>GCCCACAAGTCTTGTCTGCCTGCTTGTGTTTACGGACTTGCTGTGG<br>CCAGTGGATATGACTTTGAGAGAGAAGGATACTCTCTGGTTGGA<br>ATAGACCCTTTCCGTCTGCTTCAAAACAGCCAGGTCTTCAGTCTCA<br>TTAGACCAAATGAAAACCCAGCACATAAAAGTCAGTTGGTATGGA<br>TGGCATGCCATTCAGCAGCGTTTGAGGACCTGAGGGTATCAAGTTT<br>CATCAGAGGGACAAGAGTGGTCCCAAGAGGACAACTATCCACCAG<br>AGGAGTTCAAATTGCATCAAATGAAAACATGGAAACAATGGACTC<br>CAGCACTCTTGAATTGAGGAGCAGATACTGGGCTATAAGAACCAG<br>GAGTGGAGGAAACACCAACCAACAGAGAGCTTCTGCAGGACAAA<br>TCAGCGTACAACCCACCTTCTCAGTACAGAGAAATCTTCCCTTTGA<br>AAGAGCGACCATCATGGCGGCATTTACAGGGAACACTGAAGGCAG<br>GACCTCTGACATGAGGACTGAGATCATAAGAATGATGGAAAGTGC<br>CAAACCAGAAGATGTGTCCTTCCAGGGGCGGGGAGTCTTCGAGCT<br>CTCGGACGAAAAGGCAACGAACCCGATCGTGCCTTCCTTTGACAT<br>GAGCAACGAAGGATCTTATTTCTTCGGAGACAGTGCAGAGGAGTA<br>TGACAATTAA
DETU	NA	1-1413	**ATG**AATCCAAATCAGAAAATAGTAACCATTGGCTCCATTTCATTA<br>GGGTTGGTTGTATTCAATGTTCTACTGCATGCTGTGAGCATCATAT<br>TAACAGTGTTAGCCCTGGGGAAGAGTGAAAACAATGGAATCTGCA<br>ATGGAACTGTAGTGAGGGAATACAATGAAACAGTTAGAATAGAG<br>AAAGTGACTCAATGGTACAATACTAGCGTAGTCGAATATGTACCG<br>CATTGGAATGAGGGCACTTATATAAATAACACCGAACCAATATGT<br>GATGTCAAGGGCTTTGCACCTTTTTCCAAGGACAACGGGATAAGA<br>GTTGGCTCCAGGGGACATATTTTTGTCATAAGAGAGCCTTTCGTC<br>TCTTGTTCACCTGTAGAGTGCAGGACTTTCTTCCTCACTCAGGGAT<br>CTCTACTCAATGACAAACACTCAAATGGAACAGTGAAGGATAGAA<br>GCCCATTCAGAACTCTCATGAGTGTCGAAGTGGGCCAATCACCCA<br>ATGTATATCAAGCCAGGTTTGAAGCTGTGGCATGGTCAGCAACA<br>GCCTGTCATGATGGTAAGAAGTGGATGACGATTGGTGTAACAGGG<br>CCAGATTCTAAAGCAGTAGCAGTAGTTCATTACGGAGGGGTGCCT<br>ACTGACGTTGTTAACTCCTGGGCAGGAGATATATTAAGAACTCAG<br>GAGTCATCTTGTACTTGCATTCAAGGTAATTGTTATTGGGTAATG<br>ACTGACGGTCCTGCCAATAGACAGGCGCAGTATAGAATATACAAA<br>GCAAATCAAGGCAAAATAATTGGCCGAACAGATGTTAGCTTTAGT<br>GGAGGACATATTGAGGAATGTTCTTGTTATCCAAATGATGGTAAA<br>GTGGAATGCGTGTGTAGAGACAACTGGACGGGAACTAACAGGCCT<br>GTACTAATTATTTCGCCTGATCTCTCTTACAGGGTTGGGTATTTATG<br>TGCAGGGTTGCCCAGTGACACTCCAAGAGGGGAAGATACTCAATT<br>TGTCGGTTCATGCACTAGTCCCATGGGAAATCAGGGATATGGCGT<br>AAAAGGGTTCGGGTTTCGACAGGGAACTGATGTGTGGGTGGGG<br>CGGACAATTAGTCGAACCTCCAGATCAGGATTTGAAATAATAAGG<br>ATAAAGAATGGTTGGACGCAAACAAGCAAAGAACAGATTAGAAG<br>ACAGGTGGTTGTTGATAACTCGAATTGGTCGGGATACAGTGGGTCT<br>TTCACTTTACCAGTAGAATTGTCTGGGAGGGAATGTTTGGTTCCC<br>TGTTTTTGGGTCGAAATGATCAGAGGTAGGCCAGAAGAGAGAACA<br>ATCTGGACCTCTAGTAGCTCCATTGTAATGTGTGGAGTTGATTATG<br>AAATTGCCGATTGGTCATGGCACGATGGAGCTATTCTTCCCTTTGA<br>CATCGATAAGACGTAA
DETU	MP	-1-982	G**ATG**AGTCTTCTAACCGAGGTCGAAACGTACGTTCTCTCTATCATC<br>CCGTCAGGCCCCCTCAAAGCCGAGATCGCGCAGAGACTTGAAGAT<br>GTCTTTGCAGGGAAAAACACCGATCTCGAGGCTCTCATGGAGTGG<br>CTAAAGACAAGACCAATCCTGTCACCTCTGACTAAAGGGATTTTG

			GGATTTGTGTTCACGCTCACCGTGCCCAGTGAGCGAGGACTGCAG<br>CGTAGACGCTTCGTCCAGAATGCCCTAAATGGAAACGGGGATCCA<br>AATAATATGGATAAGGCAGTTAAGCTATATAAGAAGCTGAAAAGA<br>GAGATAACATTCCATGGGGCTAAGGAGGTCGCACTTAGCTACTCA<br>ACCGGTGCACTTGCCAGCTGCATGGGTCTCATATACAACAGGATG<br>GGAACGGTGACTACAGAAGTGGCTTTTGGCCTAGTGTGTGCCACTT<br>GTGAGCAGATTGCAGATTCACAGCATCGGTCCCACAGACAGATGG<br>CAACCATCACCAACCCATTAATCAGACATGAGAACAGAATGGTGC<br>TGGCCAGCACTACAGCTAAGGCCATGGAGCAGATGGCAGGATCAA<br>GCGAGCAGGCATCAGAAGCCATGGAGGTTGCTAATCAGGCCAGGC<br>AGATGGTACAGGCAATGAGGACAATTGGGACTCATCCTAACTCTA<br>GTGCTGGTCTGAGAGATAATCTTCTTGAAAATTTGCAGGCCTACCA<br>GAACCGAATGGGAGTGCAGATGCAGCGATTCAAGTGATCCTCTTG<br>TTGTTGCCGCAAATATCATTGGGATCCTGCACTTGATATTGTGGAT<br>CCTTGATCGTCTTTTCTTCAAATGCATTTATCGTCGCCTTAAATACG<br>GTTTGAAAATAGGGCCTTCTACGGAAGGGGTACCTGAGTCTATGA<br>GGGAAGAGTACCGGCAGGAACAGCAGAGTGCTGTGGATGTTGACG<br>ATGGTCATTTTGTCAACATAGAATTGGAGTAA
DETU	NS	2-838	**TG**GACTCCAACACTGTGTCAAGCTTTCAGGTAGACTGCTTTCTTTG<br>GCATGTCCGCAAACGATTTGCAGACCAAGAACTGGGTGATGCCCC<br>ATTCCTTGACCGGCTTCGCCGAGACCAGAAGTCCCTAAGAGGAAG<br>AGGCAGCACTCTTGGTCTGGACATCGAGACAGCTACTCGTGCGGG<br>AAAGCAAATATTGGAGCGGATTCTGGGGGAAGAATCTGATGAAGC<br>ACTTAAAATGAATATTGCTTCTGTACCGACTTCACGCTACCTAACT<br>GACATGACTCTTGAAGAAATGTCAAGAGACTGGTTCATGCTCATG<br>CCCAAGCAGAAAGTAGCAGGTTCTCTCTGCATCAAAATGGACCAG<br>GCAATAATGGATAAAACCATCATACTGAAAGCAAACTTCAGTGTG<br>ATTTTTGATCGGCTGGAAACCCTAATATTACTTAGAGCTTTCACAG<br>AAGAAGGAGCAATTGTGGGAGAAATCTCACCATTACCTTCTCTTCC<br>AGGACATACTGATGAGGATGTCAAAATTGCAATTGGGGTCCTCAT<br>CGGAGGGCTTGAATGGAATGATAACACAGTTCGAGTCTCTGAAAC<br>TCTACAGAGATTCACTTGGAGAAGCAGTAATGAGGATGGGAGACC<br>TTCACTCCCTTCAAAACAGAAACGGAAAATGGCGAGAACAATTGA<br>GTCAGAAGTTCGAGGAAATAAGATGGCTGATTGAGGAAATGCGAC<br>ATAGATTGAAGATCACAGAGAACAGCTTCGAACAAATAACGTTTA<br>TGCAAGCTTTACAACTATTGCTTGAAGTGGAGCAAGAGATAAGAA<br>CCTTCTCGTTTCAGCTTATTTAA
UKDD	PB2	1-2298	**ATG**GAGAGAATAAAAGAACTAAGAGATCTAATGTCTCAATCCCGC<br>ACTCGCGAGATACTAACAAAAACCACTGTGGACCATATGGCCATA<br>ATCAAGAAATACACATCAGGAAGACAAGAGAAGAACCCTGCTCTC<br>AGAATGAAATGGATGATGGCAATGAAATATCCAATCACAGCAGAC<br>AAGAGAATAATGGAAATGATTCCTGAAAGAAATGAACAAGGCCA<br>GACGCTTTGGAGTAAGACAAATGATGCTGGATCAGACAGAGTGAT<br>GGTGTCTCCCCTAGCTGTAACTTGGTGGAATAGAAATGGACCGAC<br>AGCAAGTACAGTCCATTATCCAAAGGTCTACAAAACATACTTTGA<br>GAAGGTTGAAAGGTTAAAGCATGGAACCTTCGGTCCCGTTCACTTC<br>CGAAACCAAATTAAAATACGCCGCCGAGTTGACATAAACCCAGGC<br>CACGCAGATCTCAGTGCCAAAGAAGCACAAGATGTCATCATGGAG<br>GTCGTTTTCCCAAATGAAGTGGGAGCTAGAATATTGACATCAGAG<br>TCACAATTGACAATAACGAAAGAGAAAAAAGAAGAACTCCAGGA<br>TTGCAAGATTGCTCCTTTAATGGTGGCATACATGTTGGAAAGAGAA<br>CTGGTCCGCAAAACCAGATTCCTACCAGTAGCAGGTGGGACAAGC<br>AGTGTGTACATTGAGGTACTGCACTTGACCCAAGGGACCTGCTGG<br>GAACAGATGTACACTCCAGGCGGAGAAGTGAGAAATGACGATGTT<br>GACCAGAGTTTGATCATCGCGGCCAGAAACATTGTTAGGAGAGCA<br>ACGGTATCAGCGGATCCACTGGCATCATTATTGGAGATGTGCCAC

			AGCACACAAATTGGTGGGACAAGGATGGTGGATATCCTTAGGCAA<br>AATCCAACTGAGGAACAAGCTGTGGATATATGCAAAGCAGCAATG<br>GGTTTAAGGATTAGTTCATCCTTTAGCTTTGGAGGATTCACCTTCA<br>AAAGAACAAGTGGTTCATCCATTAGAAAGGAAGAGGAAGTGCTTA<br>CAGGCAACCTCCAAACATTGAAAATAAGAGTACATGAGGGGTATG<br>AGGAGTTCACAATGGTTGGGCGAAGAGCAACAGCCATTCTAAGGA<br>AAGCAACTAGAAGGCTGATTCAGTTGATAGTAAGTGGAAGAGACG<br>AACAATCAATCGCTGAAGCAATCATCGTAGCCATGGTGTTCTCACA<br>GGAGGATTGCATGATAAAGGCAGTCCGAGGCGATCTAAATTTTGT<br>GAACAGAGCAAACCAAAGATTGAACCCCATGCATCAACTCCTGAG<br>ACACTTCCAAAAAGATGCAAAAGTGCTGTTTCAAAATTGGGGGAT<br>TGAACCCATTGATAATGTCATGGGGATGATTGGAATATTGCCTGAC<br>ATGACTCCAAGCACAGAGATGTCACTAAGAGGAGTAAGAGTTAGT<br>AAAATGGGAGTAGATGAATATTCCAGCACTGAGAGAGTGGTTGTA<br>AGCATTGACCGTTTCTTGCGGGTTCGAGATCAGCAGGGGAACTTAC<br>TCCTATCTCCCGAAGAAGTCAGCGAAACACTGGGAACAGAAAAGT<br>TAACAATAACATATTCATCATCAATGATGTGGGAAATCAATGGTCC<br>TGAGTCAGTGCTGGTCAACACCTATCAATGGATCATCAGAAATTG<br>GGAGATTGTGAAGATTCAATGGTCTCAAGACCCCACGATGCTGTA<br>CAATAAGGTGGAGTTTGAACCGTTCCAATCCTTGGTACCTAAAGCT<br>GCCAGAGGCCAATACAGTGGATTTGTGAGAACACTGTTCCAACAA<br>ATGCGTGACGTATTGGGGACATTTGATACTATTCAGATAATAAAGC<br>TGTTACCGTTTGCAGCAGCCCCACCGGAGCATAGCAGAATGCAAT<br>TTTCTTCCCTGACCGTGAATGTAAGAGGCTCGGGAATGAGAATACT<br>CGTAAGGGGTAACTCCCCTGTGTTCAACTACAATAAGGCAACCAA<br>AAGGCTTGCCGTCCTTGGAAAGGACGCAGGTGCATTAACAGAGGA<br>TCCAGATGAGGGGACAACAGGAGTGGAATCTGCAGTGCTGAGGGG<br>GTTCCTAATTCTGGGCAGGGAGGACAGAAGATATGGACCAGCACT<br>AAGCATCAATGAACTGAGCAATCTTGCGAAAGGGGAGAAAGCCA<br>ATGTGCTGATAGGGCAAGGAGACGTGGTGCTGGTAATGAAACGGA<br>AACGGGACTCTAGCATACTTACTGACAGCCAGACAGCGACCAAAA<br>GAATTCGGATGGTCATCAATTAGTATCGAGTTGTTTAAAAA
UKDD	PB1	1-2277	**ATG**GATGTCAACCCGACTTTACTCTTCTTGAAAGTGCCAGCGCAAA<br>ATGCTATAAGTACCACATTCCCTTATACTGGAGATCCTCCATACAG<br>CCATGGAACAGGAACAGGATACACCATGGACACAGTCAACAGAA<br>CGCATCAATACTCAGAAAAGGGAAAGTGGACAAAAAACACCGAG<br>ACTGGAGCACCCCAACTCAACCCAATTGATGGACCATTACCTGAG<br>GATAACGAGCCAAGCGGATATGCACAAACGGATTGTGTGTTGGAA<br>GCAATGGCTTTCCTTGAAGAGTCCCACCCAGGGATCTTTGAAAACT<br>CATGTCTTGAAACAATGGAAATTGTTCAACAAACAAGAGTGGACA<br>AACTGACCCAAGGTCGTCAGACCTATGACTGGACATTGAATAGAA<br>ACCAGCCGGCTGCAACTGCTTTAGCCAACACTATAGAAGTCTTCAG<br>ATCGAACGGTCTAACAGCCAATGAGTCAGGGAGACTGATAGATTT<br>CCTCAAAGATGTGATGGAGTCAATGGACAAAGAAGAAATGGAAAT<br>AACAACACATTTCCAAAGAAAGAGAAGAGTAAGAGACAATATGA<br>CCAAGAAAATGGTCACACAAAGAACAATAGGGAAGAAAAAACAG<br>AGACTGAACAAGAAGAACTACTTGGTAAGGGCACTGACACTGAAC<br>ACAATGACAAAAGATGCAGAAAGAGGCAAGTTGAAGAGGCGGGC<br>AATTGCAACACCCGGGATGCAAATCAGAGGGTTCGTGTACTTTGTC<br>GAAACATTAGCGAGGAGCATCTGCGAGAAACTTGAGCAATCTGGG<br>CTCCCTGTTGGAGGAAATGAAAAAAAGGCTAAGTTGGCAAATGTC<br>GTGAGAAAGATGATGACTAACTCACAAGACACAGAGCTATCCTTT<br>ACAATTACTGGAGACAATACCAAGTGGAACGAGAATCAGAATCCT<br>CGGATTTTTTTGGCAATGATAACATATATCACAAGAAATCAACCTG<br>AGTGGTTTAGAAATGTGTTAAGTATTGCCCCTATAATGTTCTCAAA<br>CAAAATGGCAAGATTAGGGAAAGGATACATGTTCGAAAGTAAG

			AGCATGAAGCTACGGACACAAATACCAGCAGAAATGCTTGCAACC<br>ATTGACCTGAAATATTTCAACGAATCGACAAGAAAGAAAATTGAG<br>AAAATAAGGCCTCTCCTAATAGAAGGAACAGCCTCGTTGAGTCCT<br>GGAATGATGATGGGCATGTTCAACATGCTGAGTACAGTCTTGGGA<br>GTATCAATTCTAAATCTTGGCCAAAAGAGGTACACCAAAACCACA<br>TACTGGTGGGACGGACTCCAATCCTCTGATGATTTCGCTCTCATAG<br>TAAATGCACCGAATCATGAGGGAATACAGGCAGGAGTGGACAGGT<br>TCTATAGGACTTGTAAATTGGTTGGGATCAATATGAGTAAAAAG<br>AAATCCTATATAAATCGGACAGGAACATTTGAATTCACAAGCTTTT<br>TCTACCGTTATGGGTTTGTAGCCAACTTCAGCATGGAGCTGCCCAG<br>CTTTGGAGTTTCTGGGATTAATGAATCGGCTGACATGAGCATTGGA<br>GTTACAGTAATAAAGAATAACATGATAAACAACGATCTTGGACCA<br>GCAACAGCTCAAATGGCTCTTCAGCTATTTATCAAGGACTACAGAT<br>ATACATATCGATGCCACAGGGGTGATACACAAATACAAACAAGGA<br>GATCATTCGAGCTAAAGAAGCTGTGGGAGCAGACCCGTTCAAAGG<br>CAGGACTGTTGGTTTCAGATGGAGGCCCAAACTTATACAATATAC<br>GGAATCTCCACATCCCAGAGGTCTGCTTGAAGTGGGAACTGATGG<br>ATGAAGATTACCAGGGTAGACTTTGTAATCCCCTGAACCCCTTTGT<br>CAGTCATAAGGAAATTGAATCCGTAAACAATGCTGTAGTGATGCC<br>AGCCCATGGTCCGGCCAAAAGCATGGAATATGATGCTGTTGCGAC<br>CACACACTCATGGGTCCCTAAGAGGAACCGTTCCATTCTGAATACC<br>AGTCAAAGAGGAATCCTTGAGGATGAACAGATGTATCAGAAGTGC<br>TGCAATCTATTTGAAAAATTCTTCCCTAGTAGCTCATACAGGAGGC<br>CAGTTGGAATCTCCAGTATGGTGGAGGCCATGGTGTCTAGGGCCC<br>GAATTGATGCACGGATTGACTTCGAGTCTGGTAGGATTAAGAAGG<br>AAGAGTTTGCTGAGATCATGAAGATCTGTTCCACCATTGAAGAGA<br>TCAGACGGCAAAAACAGTGA
UKDD	PA	1-2151	**ATG**GAAGACTTTGTGCGACAATGCTTCAATCCAATGATCGTCGAG<br>CTTGCGGAAAAGACAATGAAAGAATATGGGGAAAATCCAAAAAT<br>CGAAACGAACAAATTCGCTGCAATATGCACTCACTTAGAGGTCTG<br>TTTCATGTATTCGGATTTCCACTTTATTGATGAACGAGGTAAATCA<br>ATAATTGTAGAATCTGGCGATCCGAATGCATTATTGAAACACCGAT<br>TTGAGATAATTGAAGGGAGAGACCGAACGATGGCTTGGACAGTGG<br>TAAATAGTATCTGCAACACCACAGGAGTCGATAAGCCTAAATTCC<br>TCCCAGATTTGTATGATTACAAGGAGAACCGATTCATTGAAATTGG<br>AGTGACAAGGAGGGAAGTTCACACATACTACCTAGAAAAGGCAA<br>ATAAGATAAAATCAGAGAAGACACACATTCACATATTCTCATTCA<br>CTGGGGAGGAGATGGCCACCAAAGCTGATTATATCCTTGATGAAG<br>AGAGCAGAGCAAGGATCAAAACCAGGTTGTTCACTATCAGGCAA<br>GAAATGGCCAATAGGGGTCTGTGGGATTCCTTTCGTCAATCTGAGA<br>GAGGCGAAGAGACAATTGAAGAAAGGTTTGAAATCACAGGAACC<br>ATGCGCAGGCTTGCCGACCAAAGTCTCCCACCGAATTTCTCCAGCC<br>TTGAAAATTTTAGAGCCTATGTGGATGGATTCAAACCGAACGGC<br>TGCCTTGAGGGCAAGCTTTCTCAAATGTCAAAAGAAGTGAACGCC<br>AGAATTGAGCCATTCATGAAGACAACACCACGCCCTCTCAGATTA<br>CCTGATGGTCCTCCTTGCTCTCAGCGGTCGAAATTCTTACTGATGG<br>ATGCCCTTAAATTGAGCATCGAAGACCCAAGCCATGAGGGAGAAG<br>GTATACCGCTATATGATGCAATCAAATGCATGAAGACGTTTTTTGG<br>TTGGAAAGAGCCCAACATTGTAAAACCACATGTAAAAGGCATAAA<br>TCCCAACTATCTCTTGGCTTGGAAGCAGGTGCTGGTAGAACTCCAA<br>GACATTGAAAATGAAGAGAAAATCCCAAAAACAAAAAACATGAA<br>GAAAACAAGCCAACTAAAGTGGGCACTCGGTGAGAATATGGCACC<br>TGAAAAAGTGGACTTTGAGGACTGCAAAGATGTTAGCGATCTAAG<br>ACAGTATGACAGTGATGAACCAGAGCCCAGATCATTATCAAGCTG<br>GATCCAGAGCGAATTCAACAAAGCATGCGAATTGACAGATTCGAG<br>TTGGATTGAACTTGATGAAATAGGAGAAGATGTTGCTCCAATTGA

			GCACATTGCGAGTATGAGAAGAAACTACTTCACAGCGGAAGTGTC<br>TCATTGCAGGGCTACTGAATATATAATGAAAGGAGTTTATATAAAT<br>ACAGCCCTGTTGAATTCATCCTGTGCAGCCATGGATGACTTCCAAT<br>TGATTCCAATGATAAGCAAGTGCAGAACCAAAGAAGGAAGACGG<br>AAGACAAATCTATATGGGTTCATTATAAAAGGAAGATCCCATTTG<br>AGGAATGATACCGATGTGGTAAATTTTGTGAGCATGGAGTTCTCTC<br>TTACTGACCCGAGGCTGGAACCACACAAGTGGGAAAAGTACTGTG<br>TTCTCGAAATAGGAGACATGCTCCTACGAACTGCAATAGGCCAAG<br>TATCAAGACCCATGTTTCTTTATGTAAGGACCAATGGGACTTCCAA<br>GATCAAGATGAAATGGGGCATGGAGATGAGGCGATGCCTTCTTCA<br>ATCCCTCCAACAAATTGAGAGCATGATTGAGGCAGAGTCTTCTGTC<br>AAAGAGAAGGACATGACCAAGGAATTCTTTGAAAATAAATCAGAA<br>ACGTGGCCAATTGGGGAATCACCTAAGGGGGTGGAGGAAAGCTCT<br>ATTGGGAAAGTGTGTAGAACATTACTAGCAAAATCTGTATTCAAC<br>AGCCTATATGCATCTCCACAACTTGAGGGGTTTTCAGCTGAGTCGA<br>GAAAGTTACTTCTCATTGTTCAGGCATTTAGGGACAACCTGGAACC<br>TGGGACCTTCGATCTTGGGGGGCTATATGAAGCAATTGAGGAGTG<br>CCTGATTAATGATCCCTGGGTTTTGCTTAATGCATCTTGGTTCAACT<br>CCTTCCTTACACATGCACTGAAATAG
UKDD	HA	1-1704	**ATG**GAGAAAATAGTGCTTCTTCTTGCAGTGGTTAGCCTTGTTAAAA<br>GTGATCAGATTTGCATTGGTTACCATGCAAACAACTCAACAAAAC<br>AGGTTGACACAATAATGGAAAAAAACGTCACTGTTACACATGCCC<br>AAGACATACTGGAAAAGACACACAACGGGAAGCTCTGCGATCTTA<br>ATGGAGTGAAGCCCCTGATTCTAAAGGATTGTAGCGTAGCTGGGT<br>GGCTCCTTGGAAATCCAATGTGCGACGAGTTCATCAGGGTGCCGG<br>AATGGTCTTACATCGTGGAGAGGGCTAACCCAGCCAACGACCTCT<br>GTTACCCAGGGACCCTCAATGACTATGAGGAACTGAAACACCTAC<br>TGAGCAGAATAAATCATTTTGAGAAAACTCTGATCATCCCCAAGA<br>GTTCTTGGCCCAATCATGAAACATCATTAGGGGTGAGCGCAGCAT<br>GTCCATACCAGGGAGCATCCTCATTTTTCAGAAATGTGGTATGGCT<br>CATCAAAAAGAACGATGCATACCCGACAATAAAGATAAGCTACAA<br>TAATACCAATCGGGAAGATCTTTTGATACTGTGGGGGATTCATCAT<br>CCCAACAATGCAGAAGAGCAGACAAATCTCTATAAAAACCCAGAC<br>ACTTATGTTTCCGTTGGGACATCAACATTAAACCAGAGATTGGTGC<br>CAAAAATAGCTACTAGATCCCAAGTAAACGGGCAACGTGGAAGAA<br>TGGATTTCTTCTGGACAATTTTAAAACCGAATGATGCAATCCACTT<br>TGAGAGTAATGGAAATTTCATTGCTCCAGAATATGCCTACAAAATT<br>GTCAAGAAAGGGGACTCAACAATTATGAAAAGTGAAGTGGAGTAT<br>GGCTACTGCAACACCAAATGTCAAACCCCAATAGGGGCGATAAAC<br>TCTAGCATGCCATTCCACAATATACACCCTCTCACCATCGGGGAAT<br>GCCCCAAATACGTGAAGTCAAACAAATTAGTCCTTGCGACTGGGC<br>TCAGAAATAGTCCTCTAAGGGAAAGAAGAAAAAGAGGACTA<br>TTTGGAGCTATAGCAGGGTTTATAGAGGGAGGATGGCAGGGAATG<br>GTAGACGGTTGGTATGGGTACCACCATAGCAATGAGCAGGGGAGT<br>GGGTACGCTGCAGACAAAGAATCCACCCAAAAGGCAGTAGATGG<br>AGTTACCAATAAGGTCAACTCAATCATTGACAAAATGAACACTCA<br>ATTTGAGGCCGTTGGAAGGGAATTTAATAACTTAGAAAGGAGAAT<br>AGAGAATTTAAACAAGAAAATGGAAGACGGATTCCTAGATGTCTG<br>GACTTATAATGCTGAACTTTTAGTTCTCATGGAAAATGAGAGAACT<br>CTAGATTTCCATGACTCAAATGTCAAGAACCTTTACGACAAAGTCC<br>GACTACAGCTTAGGGATAATGCAAAGGAGCTGGGTAATGGTTGTT<br>TCGAGTTCTATCACAAATGTGATAACGAATGTATGGAAAGCGTAA<br>GAAATGGGACGTATGACTACCCTAAGTATTCAGAAGAAGCAAGAT<br>TAAAAAGAGAAGAAATAAGCGGAGTGAAATTAGAATCAATAGGA<br>ACTTACCAAATACTGTCAATTTATTCAACAGTGGCGAGTTCCCTAG<br>CACTGGCAATCATAGTGGCTGGTCTATCTTTATGGATGTGCTCTAA

			TGGGTCGCTACAATGCAGAATTTGCATCTAA
UKDD	NP	1-1497	**ATG**GCGTCTCAAGGCACCAAACGATCTTATGAACAGATGGAAACT<br>GGTGGAGAACGCCAGAATGCCACTGAAATCAGAGCATCTGTTGGA<br>AGAATGGTTGGTGGAATTGGAAGGTTTTATATACAGATGTGCACT<br>GAACTCAAACTCAGCAATTATGAGGGGAGACTGATCCAGAACAGC<br>ATAACAATAGAAAGAATGGTTCTCTCTGCATTTGATGAAAGGAGG<br>AACAAGTACCTGGAAGAACATCCCAGTGCGGGGAAGGACCCAAA<br>GAAAACTGGAGGTCCAATCTACAGAAGAAGAGACGGAAAGTGGA<br>TGAGGGAGCTGATTCTGTATGACAAAGAAGAGATCAGAAGGATCT<br>GGCGTCAAGCAAATAATGGAGAAGATGCAACTGCTGGTCTCACCC<br>ATCTGATGATCTGGCACTCCAACCTGAATGATGCCACATATCAGAG<br>GACAAGGGCTCTCGTGCGCACTGGAATGGATCCCAGAATGTGCTC<br>TCTGATGCAAGGATCAACTCTCCCAAGAAGGTCTGGAGCTGCTGG<br>TGCAGCAGTAAAAGGGGTCGGAACAATGGTAATGGAATTGATTCG<br>AATGATAAAGCGAGGGATTAATGATCGGAATTTCTGGAGAGGCGA<br>AAATGGAAGAAGGACAAGGATTGCCTATGAGAGAATGTGCAACAT<br>CCTCAAAGGGAAATTTCAAACAGCAGCACAAAGAGCAATGATGGA<br>TCAAGTGCGAGAAAGCAGGAATCCTGGGAATGCTGAAATTGAAGA<br>TCTCATTTTTCTGGCACGTTCTGCACTCATCCTGAGAGGATCAGTG<br>GCCCACAAGTCTTGTCTGCCTGCTTGTGTTTACGGACTTGCTGTGG<br>CCAGTGGATATGACTTTGAGAGAGAAGGATACTCTCTGGTTGGA<br>ATAGACCCTTTCCGTCTGCTTCAAAACAGCCAAGTCTTCAGTCTCA<br>TTAGACCAAATGAAAACCCAGCACATAAAAGTCAGTTGGTATGGA<br>TGGCATGCCATTCAGCAGCGTTTGAGGACCTGAGGGTATCAAGTTT<br>CATCAGAGGGACAAGAGTGGTCCCAAGAGGACAACTATCCACCAG<br>AGGAGTTCAAATTGCATCAAATGAAAACATGGAAACAATGGACTC<br>CAGCACTCTTGAATTGAGAAGCAGATACTGGGCTATAAGAACCAG<br>GAGTGGAGGAAACACCAACCAACAGAGAGCTTCTGCAGGACAAA<br>TCAGCGTACAACCCACCTTCTCAGTACAGAGAAATCTTCCCTTTGA<br>AAGAGCGACCATCATGGCGGCATTTACAGGGAACACTGAAGGCAG<br>GACCTCTGACATGAGGACTGAGATCATAAGAATGATGGAAAGTGC<br>CAAACCAGAAGATGTGTCTTTCCAGGGGCGGGGAGTCTTCGAGCT<br>CTCGGACGAAAAGGCAACGAACCCGATCGTGCCTTCCTTTGAC<br>ATGAGCAACGAAGGATCTTATTTCTTCGGAGACAGTGCAGAGGAG<br>TATGACAATTAA
UKDD	NA	4-1420	AATCCAAATCAGAAAATAGTAACCATTGGCTCCATTTCATTAGGGT<br>TGGTTGTATTCAATGTTCTACTGCATGCTGTGAGCATCATATTAAC<br>AGTGTTAGCCCTGGGGAAGAGTGAAAACAATGGAATCTGCAATGG<br>AACTGTAGTGAGGGAATACAATGAAACAGTTAGAATAGAGAAA<br>GTGACTCAATGGTACAATACTAGCGTAGTCGAATATGTACCGCATT<br>GGAATGAGGGCACTTATATAAATAACACCGAACCAATATGTGATG<br>TCAAGGGCTTTGCACCTTTTTCCAAGGACAATGGGATAAGAGTTGG<br>CTCCAGGGGACATATTTTTGTCATAAGAGAGCCTTTCGTCTCTTGT<br>TCACCTGTAGAGTGCAGGACTTTCTTCCTCACTCAGGGATCTCTAC<br>TCAATGACAAACACTCAAATGGAACAGTGAAGGATAGAAGCCCAT<br>TCAGAACTCTCATGAGTGTCGAAGTGGGCCAACCACCCAGTGTAT<br>ATCAAGCCAGGTTTGAAGCTGTGGCATGGTCAGCAACAGCCTGTC<br>ATGATGGTAATAAGTGGATGACGATTGGTGTAACAGGGCCAGATT<br>CTAAAGCAGTAGCAGTAGTTCATTACGGAGGGGTGCCTACTGACG<br>TTGTTAACTCCTGGGCAGGAGATATATTAAGAACTCAGGAGTCATC<br>TTGTACTTGCATTCAAGGTAATTGTTATTGGGTAATGACTGACGGT<br>CCTGCCAATAGACAGGCGCAGTATAGAATATACAAAGCAAATCAA<br>GGCAAAATAATTGGCCGAACAGATGTTAGCTTTAGTGGAGGACAT<br>ATTGAGGAATGTTCTTGTTATCCAAATGATGGTAAAGTGGAATGCG<br>TGTGTAGAGACAACTGGACGGGAACTAACAGGCCTGTACTAATTA<br>TTTCGCCTGATCTCTCTTACAGGGTTGGGTATTTATGTGCAGGGTT

			GCCCAGTGACACTCCAAGAGGGGAAGATACTCAATTTGTCGGTTC<br>ATGCACTAGTCCCATGGGAAATCAGGGATATGGCGTAAAAGGGTT<br>CGGGTTTCGACAGGGAACTGATGTGTGGGTGGGGCGGACAATTAG<br>TCGAACCTCCAGATCAGGATTTGAAATAATAAGGATAAAGAATGG<br>TTGGACGCAAACAAGCAAAGAACAGATTAGAAGACAGGTGGTTGT<br>TGATAACTCGAATTGGTCGGGATACAGTGGGTCTTTCACTTTACCA<br>GTAGAATTGTCTGGGAGGGAATGTTTGGTTCCCTGTTTTTGGGTCG<br>AAATGATCAGAGGTAGGCCAGAAGAGAGAACAATCTGGACCTCTA<br>GTAGCTCCATTGTAATGTGTGGAGTTGATTATGAAATTGCCGATTG<br>GTCATGGCACGATGGAGCTATTCTTCCCTTTGACATCGATAAGACG<br>TAATTTACGA
UKDD	MP	-5-982	GAAAG**ATG**AGTCTTCTAACCGAGGTCGAAACGTACGTTCTCTCTAT<br>CATCCCGTCAGGCCCCCTCAAAGCCGAGATCGCGCAGAGACTTGA<br>AGATGTCTTTGCAGGGAAAAACACCGATCTCGAGGCTCTCATGGA<br>GTGGCTAAAGACAAGACCAATCCTGTCACCTCTGACTAAAGGGAT<br>TTTGGGATTTGTGTTCACGCTCACCGTGCCCAGTGAGCGAGGACTG<br>CAGCGTAGACGCTTCGTCCAGAATGCCCTAAATGGGAACGGGGAT<br>CCAAATAATATGGATAAGGCAGTTAAGCTATATAAGAAGCTGAAA<br>AGAGAGATAACATTCCATGGGGCTAAGGAGGTCGCACTTAGCTAC<br>TCAACCGGTGCACTTGCCAGCTGCATGGGTCTCATATACAACAGG<br>ATGGGAACGGTGACTACAGAAGTGGCTTTTGGCCTAGTGTGTGCC<br>ACTTGTGAGCAGATTGCAGATTCACAGCATCGGTCCCACAGACAG<br>ATGGCAACCATCACCAACCCATTAATCAGACATGAGAACAGAATG<br>GTGCTGGCCAGCACTACAGCTAAGGCCATGGAGCAGATGGCAGGA<br>TCAAGCGAGCAGGCATCAGAAGCCATGGAGGTTGCTAATCAGGCC<br>AGGCAGATGGTACAGGCAATGAGGACAATTGGGACTCATCCTAAC<br>TCTAGTGCTGGTCTGAGAGATAATCTTCTTGAAAATTTGCAGGCCT<br>ACCAGAACCGAATGGGAGTGCAGATGCAGCGATTCAAGTGATCCT<br>CTTGTTGTTGCCGCAAATATCATTGGGATCCTGCACTTGATATTGT<br>GGATCCTTGATCGTCTTTTCTTCAAATGCATTTATCGTCGCCTTAAA<br>TACGGTTTGAAAATAGGGCCTTCTACGGAAGGGGTACCTGAGTCT<br>ATGAGGGAAGAGTACCGGCAGGAACAGCAGAGTGCTGTGGATGTT<br>GACGATGGTCATTTTGTCAACATAGAATTGGAGTAA
UKDD	NS	-5-849	ACATA**ATG**GACTCCAACACTGTGTCAAGCTTTCAGGTAGACTGCTT<br>TCTTTGGCATGTCCGCAAACGATTTGCAGACCAAGAACTGGGTGAT<br>GCCCCATTCCTTGACCGGCTTCGCCGAGACCAGAAGTCCCTAAGA<br>GGAAGAGGCAGCACTCTTGGTCTGGACATCGAGACAGCTACTCGT<br>GCGGGAAAGCAAATATTGGAGCGGATTCTGGGGGAAGAATCTGAT<br>GAAACACTTAAAATGAATATTGCTTCTGTACCGACTTCACGCTACC<br>TAACTGACATGACTCTTGAAGAAATGTCAAGAGACTGGTTCATGCT<br>CATGCCCAAGCAGAAAGTAGCAGGTTCTCTCTGCATCAAAATGGA<br>CCAGGCAATAATGGATAAAACCATCATACTGAAAGCAAACTTCAG<br>TGTGATTTTTGATCGGCTGGAAACCCTAATATTACTTAGAGCTTTC<br>ACAGAAGAAGGAGCAATTGTGGGAGAAATCTCACCATTACCTTCT<br>CTTCCAGGACATACTGATGAGGATGTCAAAATTGCAATTGGGGTC<br>CTCATCGGAGGGCTTGAATGGAATGATAACACAGTTCGAGTCTCT<br>GAAACTCTACAGAGATTCACTTGGAGAAGCAGTAATGAGGATGGG<br>AGACCTTCACTCCCTTCAAAACAGAAACGGAAAATGGCGAGAACA<br>ATTGAGTCAGAAGTTCGAGGAAATAAGATGGCTGATTGAGGAAAT<br>GCGACATAGATTGAAGATCACAGAGAACAGCTTCGAACAAATAAC<br>GTTTATGCAAGCTTTACAACTATTGCTTGAAGTGGAGCAAGAGATA<br>AGAACCTTCTCGTTTCAGCTTATTTAATGATAA
