# Supplementary material for: Comparison of sequencing methods and data processing pipelines for whole genome sequencing and minority single nucleotide variant (mSNV) analysis during an influenza A/H5N8 outbreak
Source: PLoS One. 2020 Feb 20;15(2):e0229326. doi: 10.1371/journal.pone.0229326 (PMC7032710; doi:10.1371/journal.pone.0229326)
Supplement: S3 Table — (PDF) [file pone.0229326.s003.pdf]

Sample	SP	Platform	Method	Reads	Nucleotides	Influenza reads	Influenza Nucleotides
**DETU**	1	Illumina MiSeq	RNA-Seq+PCR	35,397,942	4,768,436,983	ca. 21,238,765	ca 2,861,062,190
	3	454	Amplicon	78,028	25,829,288	75,913	25,692,541
	2	Illumina MiSeq	RNA Shot gun	1,394,424	417,805,080	1,062,401	318,461,282
**NLCH**	1	Illumina MiSeq	RNA-Seq+PCR	45,091,902	6,487,449,580	1,454,528	203,647,299
	3	454	Amplicon	32,661	12,458,090	32,661	12,458,090
	2	Illumina MiSeq	RNA Shot gun	1,148,978	344,137,436	373,742	112,011,370
**UKDD**	1	Illumina MiSeq	RNA-Seq+PCR	10,214,524	768,562,277	867,355	64,794,700
	3	454	Amplicon	49,993	18,897,160	48,769	18,821,757
	2	Illumina MiSeq	RNA Shot gun	1,512,512	421,870,650	1,039,962	294,863,446
