# Supplementary material for: Comparison of sequencing methods and data processing pipelines for whole genome sequencing and minority single nucleotide variant (mSNV) analysis during an influenza A/H5N8 outbreak
Source: PLoS One. 2020 Feb 20;15(2):e0229326. doi: 10.1371/journal.pone.0229326 (PMC7032710; doi:10.1371/journal.pone.0229326)
Supplement: S1 File — (PDF) [file pone.0229326.s004.pdf]

Software: CLC Genomics Workbench 8

Black = applied for SP1,SP2 and SP3 data
Blue = applied for SP3 data
Green = applied for SP1 and SP2 data

1. **Demultiplex**

   File: JGJ0HAZ01.sff
   Define tags:
   - Barcode length: 11
   - Sequence: 1-1000

   Set barcode options:
   - Search both strands = yes
   - Barcodes: MID sequences Roche (1-6)

   Result handling:
   - Create list of reads without barcode = yes
   - Create report = no
   - Save = yes

1. **Workflow Map reads to reference beta**
   a. **Trim sequences**
   Quality trimming:
   - Ambiguous trim = Yes
   - Ambiguous limit = 2
   - Quality trim = Yes
   - Quality limit = 0,05 = phred score = 20

   Adapter trimming:
   - Trim adapter list = NA Trim adapter library 2
   - Use colorspace = No
   - Search on both strands: Yes

   Sequence filtering:
   - Remove 5' terminal nucleotides = No Yes
   - Number of 5' terminal nucleotides = NA 30
   - Remove 3' terminal nucleotides = No Yes
   - Number of 3' terminal nucleotides = NA 30
   - Discard short reads = Yes
   - Minimum number of nucleotides in reads = 15
   - Discard long reads = Yes
   - Maximum number of nucleotides in reads = 1.000

   Result handling:
   - Save discarded sequences = Yes
   - Save broken pairs = No
   - Create report = Yes
   - Result handling: Save

➔ SP3 data files saved as *.fastq and shared via DataHub

   b. **Map reads to reference data**

Select sequencing reads

Trimmed reads. For 454 data enter both MID's.

References

Use reference files mentioned above. Consensus sequences per sample derived from the consensus sequences of the different institutions

Masking mode = No masking

Exclude annotated = NA

Include annotated only = NA

Mapping options:

Mismatch cost = 2

Cost of insertions and deletions = Affine gap cost

Insertion open cost = 7

Insertion extend cost = 2

Deletion open cost = 7

Deletion extend cost = 2

Length fraction = 0,7

Similarity fraction = 0,9

Global alignment = No

Non-specific match handling = Map randomly

Results handling:

Output mode = Create stand-alone read mappings

Create report = Yes

Collect un-mapped reads = Yes

Save = yes

2. Workflow Realign and detect variants

Local realignment

Realignment settings:

Realign unaligned ends = Yes

Multi-pass realignment = 2

Guidance-variant track = Not set

Result handling:

Output mode = Create reads track

Output track of realigned regions = No

Indels and Structural variants

Select read mappings

Locally realigned file

Select settings

P-Value threshold = 0,0001

Maximum number of mismatches = 3

Filter variants = Yes

Minimum number of reads = 2

Reference masking: NA

Result handling:

Create report = No

Create breakpoints = No

Create InDel variants = Yes

Create structural variations = No

Save = yes

Local realignment

Realignment settings:
Realign unaligned ends = Yes
Multi-pass realignment = 2
Guidance-variant track = Locally realigned (InDel)-file
Force realignment to guidance-variants = No
Result handling:
Output mode = Create reads track
Output track of realigned regions = No

Low frequency variant detection
Select read mappings
Locally realigned – locally realigned files
Low frequency variant parameters
Required significance (%) = 1,0
General filters
Ignore positions with coverage above = 100.000
Restrict calling to target regions = Not set
Ignore broken pairs = Yes
Ignore non-specific matches = Reads
Minimum coverage = 2
Minimum count = 2
Minimum frequency (%) = 1,0
Noise filters
Base quality filter = Yes
Neighborhood radius = 5
Minimum central quality = 0
Minimum neighborhood quality = 0
Read direction filter = Yes
Direction frequency (%) = 5,0
Relative read direction filter = Yes
Significance (%) = 1,0
Read position filter = Yes
Significance (%) = 1,0
Remove pyro-error variants = No (454 data checked with and without, no difference for mSNV identification)
Result handling:
Create track = Yes
Create annotated table = Yes
Create report = No
