# Supplementary figures and images for: Comparison of sequencing methods and data processing pipelines for whole genome sequencing and minority single nucleotide variant (mSNV) analysis during an influenza A/H5N8 outbreak
Source: PLoS One. 2020 Feb 20;15(2):e0229326. doi: 10.1371/journal.pone.0229326 (PMC7032710; doi:10.1371/journal.pone.0229326)

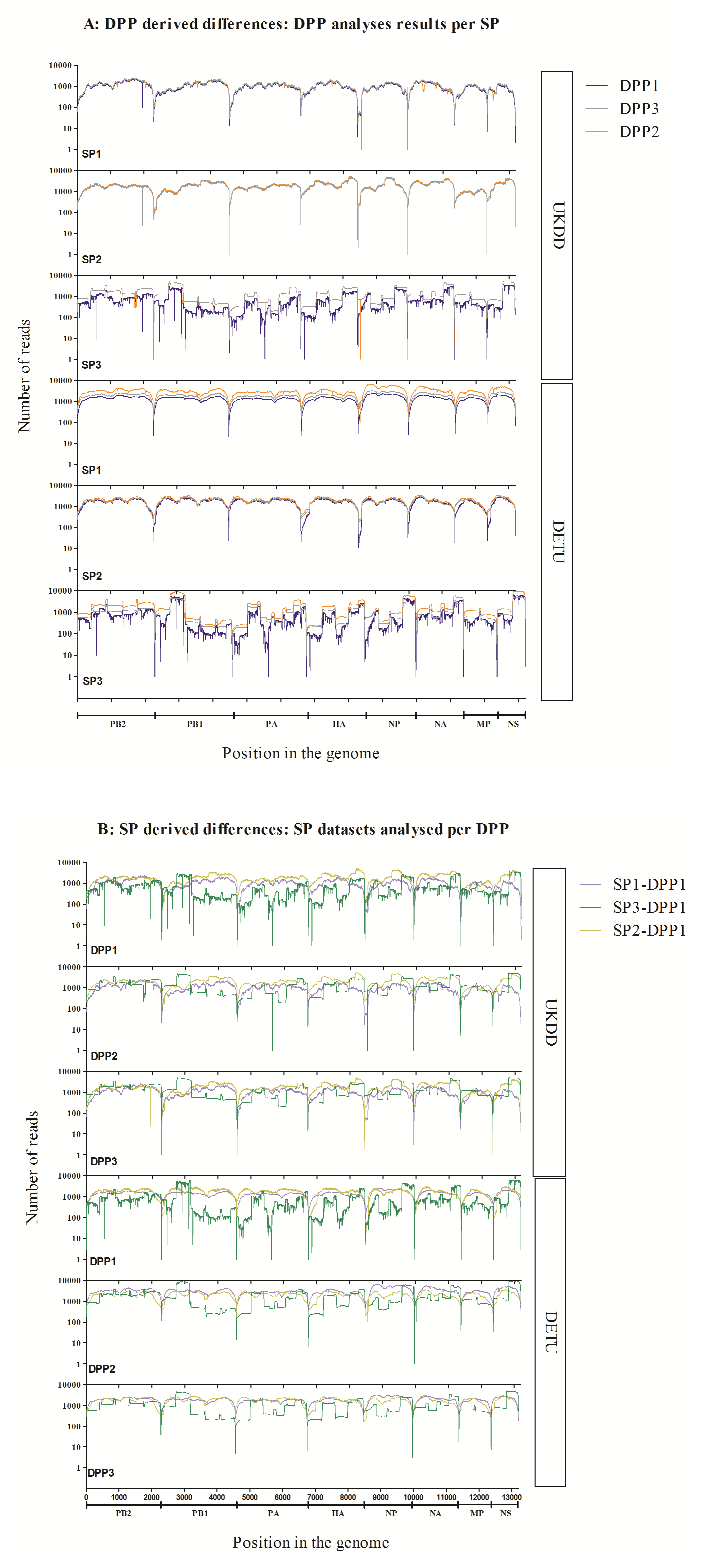

Supplement: S1 Fig — The non-normalised nucleotide coverage displayed as number of nucleotides per position for full genome sequences of the UKDD and DETU virus reads mapped to the corresponding reference sequences. Panel A shows the coverage results for the same SP dataset in the three different DPPs (DPP1: purple; DPP2: orange; DPP3 grey) for each of the SP datasets. Panel B shows the coverage when the same DPP is used to analyse data from the three different SPs (SP1: lilac; SP2: yellow; SP3:green) for each of the DPPs. The X-axis represents the position in the genome, the Y-axis represents the number of sequence reads per position. (TIF) [file pone.0229326.s005.tif]
